# Topoisomerase inhibitors promote cancer cell motility via ROS-mediated activation of JAK2-STAT1-CXCL1 pathway

**DOI:** 10.1186/s13046-019-1353-2

**Published:** 2019-08-22

**Authors:** Jiafei Liu, Like Qu, Lin Meng, Chengchao Shou

**Affiliations:** Key laboratory of Carcinogenesis and Translational Research (Ministry of Education/Beijing), Department of Biochemistry and Molecular Biology, Peking University Cancer Hospital and Institute, 52 Fucheng Road, Beijing, 100142 China

**Keywords:** Topoisomerase inhibitors, Motility, CXCL1, JAK2-STAT1, Reactive oxygen species, PTP1B

## Abstract

**Background:**

Topoisomerase inhibitors (TI) can inhibit cell proliferation by preventing DNA replication, stimulating DNA damage and inducing cell cycle arrest. Although these agents have been commonly used in the chemotherapy for the anti-proliferative effect, their impacts on the metastasis of cancer cells remain obscure.

**Methods:**

We used the transwell chamber assay to test effects of Topoisomerase inhibitors Etoposide (VP-16), Adriamycin (ADM) and Irinotecan (CPT-11) on the migration and invasion of cancer cells. Conditioned medium (CM) from TI-treated cells was subjected to Mass spectrometry screening. Gene silencing, neutralizing antibody, and specific chemical inhibitors were used to validate the roles of signaling molecules.

**Results:**

Our studies disclosed that TI could promote the migration and invasion of a subset of cancer cells, which were dependent on chemokine (C-X-C motif) ligand 1 (CXCL1). Further studies disclosed that TI enhanced phosphorylation of Janus kinase 2 (JAK2) and Signal transducers and activators of transcription 1 (STAT1). Silencing or chemical inhibition of JAK2 or STAT1 abrogated TI-induced CXCL1 expression and cell motility. Moreover, TI increased cellular levels of reactive oxygen species (ROS) and promoted oxidation of Protein Tyrosine Phosphatase 1B (PTP1B), while reduced glutathione (GSH) reversed TI-induced JAK2-STAT1 activation, CXCL1 expression, and cell motility.

**Conclusions:**

Our study demonstrates that TI can promote the expression and secretion of CXCL1 by elevating ROS, inactivating PTP1B, and activating JAK2-STAT1 signaling pathway, thereby promoting the motility of cancer cells.

**Electronic supplementary material:**

The online version of this article (10.1186/s13046-019-1353-2) contains supplementary material, which is available to authorized users.

## Background

Many cancer-related deaths are due to metastatic spread of cancer cells [1]. The clinical benefit of chemotherapy on the survival and quality of life has been demonstrated in several types of cancer [2–4]. However, chemotherapy-induced metastasis has also been noticed. Cyclophosphamide could enhance fibrosarcoma metastasis to lung in mice [5, 6]. Besides, cyclophosphamide induces metastasis in the peripheral vessels of fibrosarcoma [7]. Another chemotherapeutic agent, Carboplatin, could increase metastasis of melanoma to lung in mice [8]. Metastasis of breast cancer cells in lung has been found to be exacerbated by treatment with Paclitaxel [9–11], ADM [12], or 5-fluorouracil [13]. ADM treatment induces a stem-like phenotype and promotes metastatic potential of osteosarcoma cells [14]. Moreover, pretreatment with cisplatin and paclitaxel significantly enhances colon carcinoma and melanoma metastasis to lung [15].

Several mechanisms have been proposed to explain chemotherapy-induced metastasis. The metastatic potential of cancer cells depends on its interaction with the homeostatic factors that promote cancer cell growth, survival, angiogenesis, invasion and metastasis [16]. The density of pre-metastatic micro-environment is increased by paclitaxel in mice [9]. Paclitaxel drives metastasis in mouse models of breast cancer, which is dependent on stress-inducible gene Atf3 of non-cancer host cells [10]. In response to paclitaxel, increased annexin-6 secretion through tumor-derived exosomes could create a favorable environment for metastasis [11]. Pretreatment with cisplatin and paclitaxel significantly enhances the expression of VEGF receptor 1 on endothelial cells in vitro and in vivo, thereby enhancing the homing and retention of cancer cells within the metastatic niche [15]. In addition, plasma from paclitaxel-treated mice promotes metastasis of bone marrow-derived cells in lung by inducing matrix metalloproteinase-9 and epithelial mesenchymal transition [17]. Paclitaxel also promotes breast cancer metastasis in a TLR4-dependent manner [18]. Exposure of colon cancer cells to VP-16 at non-lethal concentrations induces Caveolin-1-dependent migration and metastasis [19]. Notably, almost all the chemotherapeutic agents could elicit DNA damage response and DNA damage has been demonstrated to induce the release of pro-survival cytokines via IL-6-Timp-1-p38 pathway [20]. Moreover, DNA damage response is involved in leptomeningeal metastasis of non-small cell lung cancer [21]. DNA damage also activates metastasis-related gene through EPC1/E2F1 pathway [22]. Telomeric DNA damage signaling regulates cancer stem cell evolution, epithelial mesenchymal transition, and metastasis [23]. ATM activates JAK/STAT3 signaling in cisplatin-resistant lung cancer cells, while inhibition of ATM inhibits invasion and metastasis [24]. Recent studies highlight DNA damage-activated cGAS-cGAMP-STING pathway in stimulating the inflammatory response as well as metastasis [25]. On the other hand, cGAMP transfer via carcinoma-astrocyte gap junctions activates STAT1 and NF-κB pathways, thereby promoting brain metastasis of breast and lung cancer cells [26].

TI represents one of the major classes of anticancer agents, since rapidly dividing cancer cells need to replicate DNA continuously and topoisomerases are essential enzymes for DNA replication. There are two classes of topoisomerases, topoisomerase I and II. Topoisomerase I inhibitor CPT-11, Topoisomerase II inhibitors VP-16 and ADM have broad spectrum of anticancer activities [27–29]. CPT-11 and VP-16 are used for the chemotherapy of small cell lung cancer [30]. CPT-11 is utilized in the treatment of colorectal cancer, especially for the metastatic colorectal cancer [31].

Although VP-16 was found to promote the motility of HT29 colon cancer cells [19], the signaling events dictating this effect are largely unknown. Furthermore, impact of other clinically used TI on the motility of cancer cells remains to be determined. Herein, we addressed these issues by showing the pro-invasive effect of VP-16, ADM and CPT-11. We further demonstrated that TI-promoted cell motility is regulated by JAK2-STAT1-CXCL1 pathway, which is associated with ROS-induced PTP1B oxidization. Our findings uncovered a novel mechanism whereby chemotherapy agents-stimulated cancer cell motility.

## Methods

### Cell lines and cell culture

The human colorectal cancer cell lines LoVo, SW480, SW620, gastric cancer cell line AGS and small cell lung cancer cell line NCI-H446 were obtained from ATCC. Cell lines were maintained in RPMI-1640 medium (Invitrogen, Carlsbad, CA, USA) supplemented with 10% fetal calf serum (Invitrogen). Cells were cultured at 37 °C with 5% CO_2_.

### RNA interference

Small interfering RNAs (siRNAs) were provided by GenePharma (Shanghai, China) and transfected into cells with siRNA Mate (GenePharma) following the provider’s instructions.

Interference sequences used were:

CXCL1 #1 -sense:

5′- CUCCAGUCAUUAUGUUAAUTT -3′;

CXCL1 #1 -antisense:

5′- AUUAACAUAAUGACUGGAGTT -3′;

CXCL1 #2 -sense:

5′-GCGGAAAGCUUGCCUCAAUTT -3′;

CXCL1 #2 -antisense:

5′-AUUGAGGCAAGCUUUCCGCTT -3′;

STAT1 #1 -sense:

5′-GCUGGAUGAUCAAUAUAGUTT-3′;

STAT1 #1 -antisense:

5′ -ACUAUAUUGAUCAUCCAGCTT -3′;

STAT1 #2 -sense:

5′- GUGGCAAAGAGUGAUCAGATT-3′;

STAT1 #2 -antisense:

5′ - UCUGAUCACUCUUUGCCACTT-3′;

STAT1 #3 -sense:

5′-GACCAUGCCUUUGGAAAGUTT -3′;

STAT1 #3 -antisense:

5′ -ACUUUCCAAAGGCAUGGUCTT-3′;

JAK2 #1 -sense:

5′-GGAUGGCAGUGUUAGAUAUTT -3′;

JAK2 #1 -antisense:

5′ -AUAUCUAACACUGCCAUCCTT -3′;

JAK2 #2 -sense:

5′-CCACCUGAAUGCAUUGAAATT -3′;

JAK2 #2 -antisense:

5′ -UUUCAAUGCAUUCAGGUGGTT -3′;

JAK2 #3 -sense:

5′- CCUGGUGAAAGUCCCAUAUTT-3′;

JAK2 #3 -antisense:

5′ -AUAUGGGACUUUCACCAGGTT − 3′;

Negative control -sense:

5′- UUCUCCGAACGUGUCACGUTT − 3′;

Negative control -antisense:

5′ -ACGUGACACGUUCGGAGAATT − 3′.

### Proliferation assay

Cells were cultured in triplicate wells of 24-well plate (1.5 × 10^4^ cells/well). The confluences were quantified by the CloneSelect Imager (Molecular Devices, Sunnyvale, CA, USA) every 24 h.

### Cell migration and invasion assays

Cell migration assay was performed in 24-well CIM plates (BD Biosciences, CA, USA). Briefly, 1–3 × 10^4^ cells per well were seeded in serum-free medium plus indicated drugs in the upper compartment of the CIM plates. Serum-complemented medium was added to the lower compartment of the chamber. After 24 h incubation, cells that passed through the septum were fixed with cold methanol and stained with crystal violet. The average number of migrated cells in four random microscopic fields was counted. Cell invasion assay was performed in 24-well CIM plates coated with matrigel. Other steps were identical to those of cell migration assay. VP-16 was provided by Hengrui Medicine (Jiangsu, China), ADM and CPT-11 were provided by Pfizer (New York, NY, USA). Fludarabine (HY-B0069), Bay11–7082 (HY-13453), C-176 (HY-112906), KU55933 (HY-12016) and AG490 (HY-12000) were purchased from MCE (Middlesex County, NJ, USA). GSH was purchased from Beyotime (Beijing, China).

### Western blot analysis

Cells were homogenized in loading buffer (0.1 M Tris-HCl, pH 6.8, 1% SDS, 10% β-mercaptoethanol, 11% glycerol), separated by SDS-PAGE and electro-blotted to the nitrocellulose membranes, then blocked with 5% non-fat milk in TBS for 1.5 h at room temperature. The membranes were incubated with the indicated primary antibodies at 4 °C overnight. After washing three times with TBST, membranes were probed by horseradish peroxidase-labeled secondary antibodies for 45 min at room temperature. Protein bands were visualized by enhanced chemiluminescence. Antibodies against p-STAT1 (Tyr701) (#9167), p-JAK2 (T1007/1008) (#3776S) and JAK2 (#3230S) were from Cell Signaling (Boston, MA, USA). Anti-STAT1 (ab2415) and HRP-Protein A (ab7456) were from Abcam (Cambridge, UK). Anti-CXCL1 (100288-T36) was from Sino Biological (Beijing, China). Anti-oxPTP (MAB2844) was from R&D systems (Minneapolis, MN, USA). Anti-PTP1B (MABS197) was from EMD Millopore (Temecula, CA, USA). Anti-GAPDH (60004) was from Proteintech (Chicago, IL, USA).

### RNA extraction and quantitative real-time PCR

Total RNA was extracted from cell pellets using Trizol reagent (Invitrogen) according to the manufacturer’s instructions. The RNA samples with an OD260/OD280 ratio between 1.9 and 2.0 were used for cDNA synthesis using High Capacity RNA to cDNA kits (Promega, Madison, WI, USA). Quantitative PCR was performed using SYBR Green master mix (Applied Biosystems) with the housekeeping gene GAPDH as the internal control. The relative expression of CXCL1 was calculated using the comparative Ct method. The primers of CXCL1 and GAPDH were as follows:

CXCL1 forward, 5′- AGCTTTGTTTAAACATGGCCCGCGCTGCTCTC-3′,

CXCL1 reverse, 5′- AGCTTTGTTTAAACCCCTTCTGGTCAGTTGGATTTG-3′;

GAPDH forward, 5′- GGAGCGAGATCCCTCCAAAAT-3′.

GAPDH reverse, 5′- GGCTGTTGTCATACTTCTCATGG-3′.

### In vivo metastasis experiments

Animal study was approved by the independent ethics committee of Peking University Cancer Hospital. NOD/SCID mice (HFK Bio-Technology, Beijing, China) were maintained in accordance with the ethics standards of the World Medical Association (Declaration of Helsinki). TI-treated cells were injected to the caudal vein of 16–19 g female NOD/SCID mice as a 100 μL suspension (5 × 10^5^ cells). After 56 days, mice were sacrificed and lungs were stripped for analysis.

### Hematoxylin-eosin staining

Lungs were immersed in 4% paraformaldehyde for 24 h and transferred to 60% ethanol. Individual lobes of lung biopsy material were placed in processing cassettes, dehydrated through the serial alcohol gradients, and embedded in paraffin. Before immunostaining, 5-μm thick lung tissue sections were dewaxed in xylene, rehydrated through decreasing concentrations of ethanol, and washed in PBS. Then sections were stained with hematoxylin and eosin. After staining, sections were dehydrated through increasing concentrations of ethanol and xylene.

### Mass spectrometric detection

Cancer cells grown in 100 mm-cell-culture dishes (80–90% confluence) were treated with VP-16 (20 μM) or DMSO in complete medium for 0.5 h. After removing culture medium and washing with PBS twice to clean the residual drug, cells were cultured in serum-free culture medium for 4 h. For each sample, conditioned medium from 12 dishes (totally 100 ml) was subjected to Mass spectrometric detection by PTM BIO (Hangzhou, China). Samples were sonicated three times on ice using a high intensity ultrasonic processor (Scientz) in lysis buffer (8 M urea plus 1% Protease Inhibitor Cocktail), followed by centrifugation at 12,000 g at 4 °C for 10 min. The protein concentration was determined with BCA kit according to the manufacturer’s instructions. For digestion, the samples were reduced with 5 mM DTT for 30 min at 56 °C and alkylated with 11 mM iodoacetamide for 15 min at room temperature in darkness. The samples were then diluted by adding 100 mM Triethylamonium bicarbonat (TEAB) to urea concentration less than 2 M. Finally, trypsin was added at 1:50 trypsin-to-protein mass ratio for the first digestion overnight and 1:100 trypsin-to-protein mass ratio for a second 4-h digestion. Next, peptides were desalted by Strata X C18 SPE column (Phenomenex) and vacuum-dried. Peptides were reconstituted in 0.5 M TEAB and processed with Tandem Mass Tag (TMT) kit. Briefly, one unit of TMT reagent was thawed and reconstituted in acetonitrile. The peptides were then incubated for 2 h at room temperature and pooled, desalted and dried by vacuum centrifugation. The peptides were subjected to nanospray ionization source followed by tandem mass spectrometry (MS/MS) in Q Exactive Plus (Thermo) coupled online to the UPLC. The electrospray voltage applied was 2.0 kV. The m/z scan range was 350 to 1800 for full scan, and intact peptides were detected in the Orbitrap at a resolution of 70,000. Peptides were then selected for MS/MS using normalized collision energy setting as 28 and the fragments were detected in the Orbitrap at a resolution of 17,500. A data-dependent procedure that alternated between one MS scan followed by 20 MS/MS scans with 15.0 s dynamic exclusion. Automatic gain control was set at 5E4. Fixed first mass was set as 100 m/z.

### Measurements of ROS

Intracellular ROS levels were evaluated using the Reactive Oxygen Species Assay Kit (Beyotime) as per manufacturer’s instruction. Briefly, cells were pulsed with 2,7-Dichlorodi -hydrofluorescein diacetate (DCFH-DA) probe (10 μM) in serum-free medium for 20 min. After washing three times with serum-free culture medium, the cells were treated with indicated agents or Rosup (positive control, 50 μg/ml) for 0.5 h. A Zeiss LSM780 confocal microscope (Carl-Zeiss, Oberkochen, Germany) was used to acquire images and the fluorescence intensity of DCFH-DA was quantified by ImageJ software.

### Statistical analysis

Statistical analysis was performed with SPSS 20.0 or GraphPad Prism version 6.0. All values were represented as the Mean ± S.D. of three to four independent experiments with triplicate wells. The unpaired two-tailed *t*-test was used for in vitro study and one-way ANOVA was used for in vivo study. A two-sided *p* < 0.05 was considered statistically significant. ** *p* < 0.01; * *p* < 0.05; N.S. *p* > 0.05.

## Results

### Topoisomerase inhibitors VP-16, ADM and CPT-11 promote cancer cell motility

**Fig. 1:**
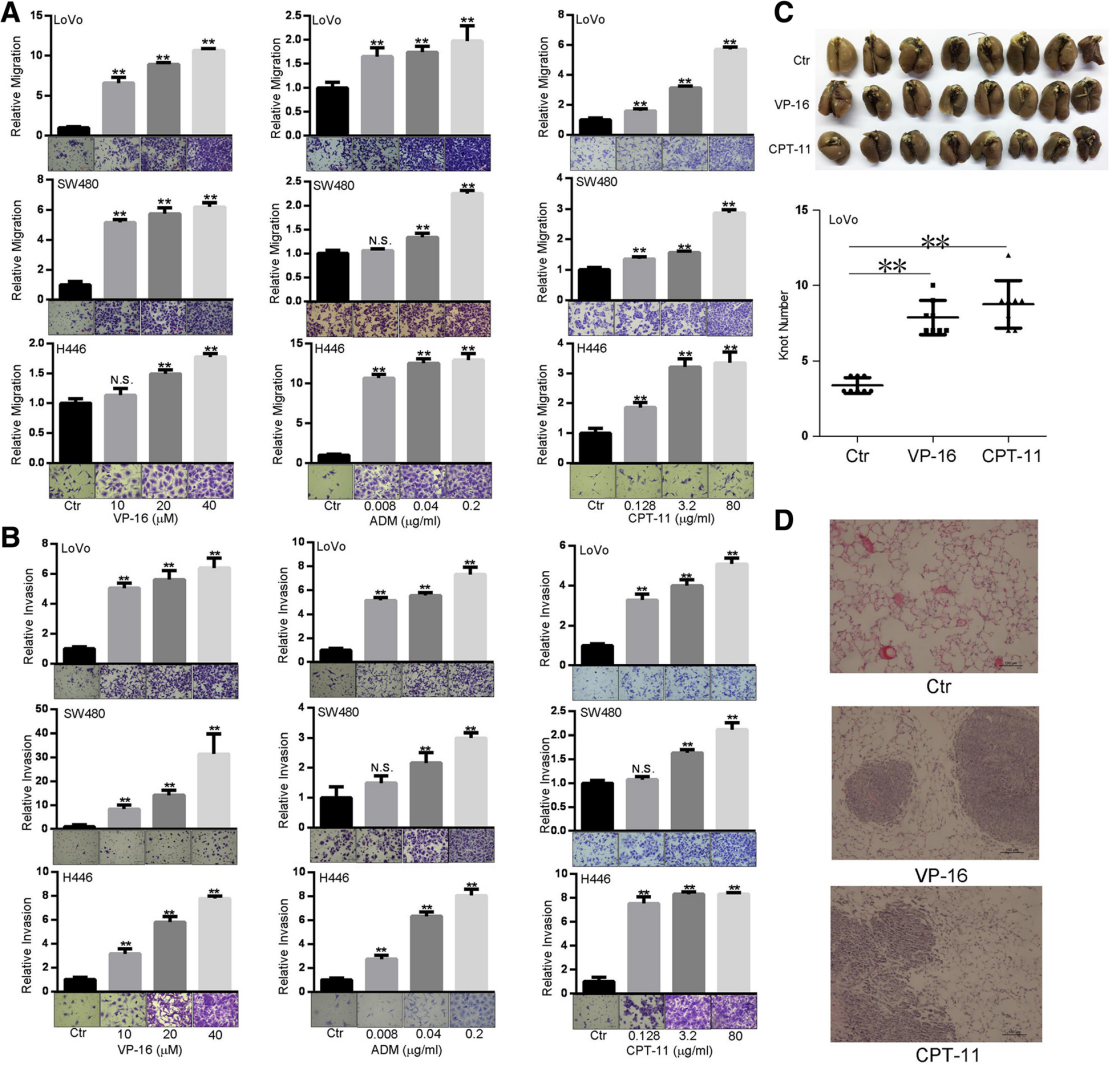
Effects of topoisomerase inhibitors on cell motility in vitro and in vivo. **a**, **b** VP-16, ADM and CPT-11 promote the migration (**a**) and invasion (**b**) of LoVo, SW480 and H446 cells. Cells were subjected to transwell chamber assays for 24 h in the presence of indicated concentrations of agents. **c** VP-16 and CPT-11 promote the metastasis in vivo. After treatment with VP-16 (20 μM) or CPT-11 (80 μg/ml) for 0.5 h, LoVo cells (5 × 10^5^ per animal) were injected to the caudal vein of NOD/SCID mice (*n* = 8 per group). After 56 days, lungs were stripped for counting nodules. Upper, macroscopic observation of lungs of mice. Lower, graph summarizing the number of metastasis nodules. **d** Hematoxylin-eosin staining of the lung tissues of mice

We firstly treated five cancer cell lines (LoVo, SW480, SW620, AGS, H446) with topoisomerase II inhibitors VP-16, ADM and topoisomerase I inhibitor CPT-11. Consistent with the anti-proliferative capacity [27–29], all these agents inhibited cell growth (Additional file 1: Figure S1). Subsequently, we performed migration and invasion assays. Although three TI inhibited the motility of AGS cells (Additional file 1: Figure S2A), the motility of SW620 cells was not affected (Additional file 1: Figure S2B). Interestingly, these TI promoted migration and invasion of LoVo, SW480, and H446 cells in a dose-dependent manner (Fig. 1a, b). To evaluate the influence of TI on cancer cell motility in vivo, we injected VP-16 or CPT-11-treated LoVo cells into caudal vein of NOD/SCID mice. We noticed that TI-treated LoVo cells had stronger ability to form metastatic nodules on the surface of lungs (Fig. 1c). The Hematoxylin-eosin staining of sectioned lung tissues also showed that TI-treated groups had more metastases (Fig. 1d). These results suggest that VP-16, ADM and CPT-11 promote motility of a subset of cancer cells in vitro and in vivo.

### TI-promoted motility is associated with increased expression and secretion of CXCL1

VP-16, ADM and CPT-11 are potent genotoxic stress inducers and DNA damage partially contributes to the anti-proliferative effect of TI [32]. Recently, ATM, and NF-κB, and cGAS-cGAMP-STING pathways were implicated in DNA damage-promoted cell motility and metastasis [25, 33, 34]. To evaluate these pathways’ roles in TI-induced cancer motility, we utilized specific chemical inhibitors. We noticed that pretreatment with inhibitors to ATM, NF-κB, or STING did not prevent VP-16 or CPT-11-promoted migration (Additional file 1: Figure S3A-C), suggesting that TI-promoted motility is independent of the activation of ATM, and NF-κB, or cGAS-cGAMP-STING.

**Fig. 2:**
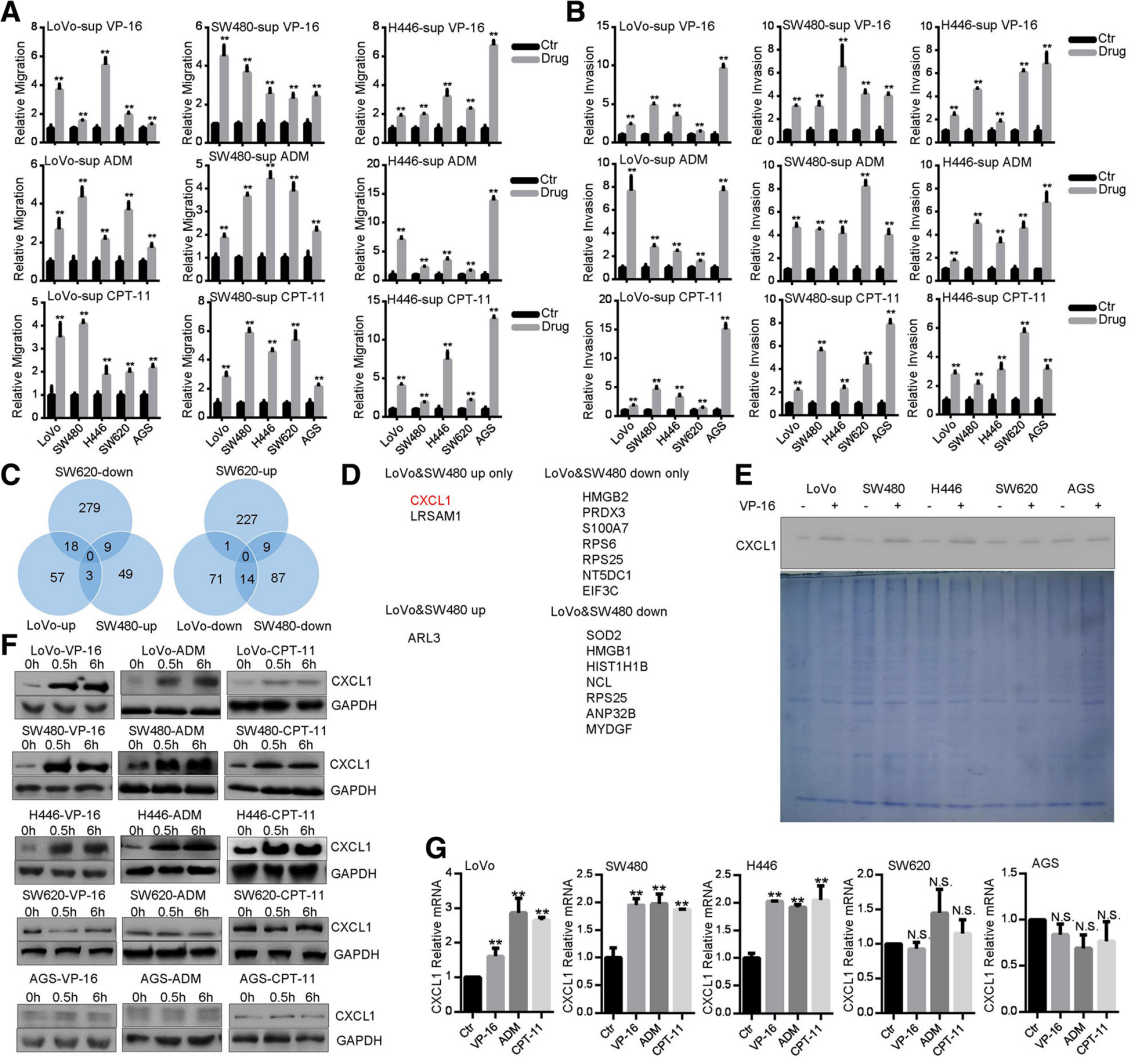
Topoisomerase inhibitors promote CXCL1 expression and secretion. **a**, **b** CM from TI-treated cells promotes migration (**a**) and invasion (**b**). Cells (LoVo, SW480, H446) were treated with VP-16 (20 μM), CPT-11 (80 μg/ml), or ADM (0.2 μg/ml) for 0.5 h in complete medium. After removing medium and washing with PBS, cells were cultured in serum-free culture medium for 4 h. CM was collected and used for transwell assays for indicated cells. **c** Wynn diagram of differential protein expression profiles in CM of LoVo, SW480 and SW620 cells. **d** The list of differentially expressed proteins. **e** Western blot analysis of CXCL1 in the CM. Indicated cells were treated with VP-16 (20 μM) for 0.5 h in complete medium, and CM was collected as in (**a**, **b**). Part of CM was resolved by SDS-PAGE and gel was stained with coomassie blue. **f** Western blot analysis of CXCL1 expression in cells after treatment with VP-16 (20 μM), CPT-11 (80 μg/ml), or ADM (0.2 μg/ml) for 0.5 and 6 h. **g** qRT-PCR analysis of CXCL1 mRNA after treatment with VP-16 (20 μM), CPT-11 (80 μg/ml), or ADM (0.2 μg/ml) for 0.5 h

Unexpectedly, serum-free CM from TI-treated cells enhanced both migration and invasion of LoVo, SW480 and H446 cells (Fig. 2a, b). Albeit direct treatment with TI failed to increase the motility of AGS and SW620 cells (Additional file 1: Figure S2A-B), CM from TI-treated cells did enhance migration and invasion of these two cell lines (Fig. 2a, b). We then speculated that secreted factors form TI-treated cells may stimulate cell motility. To characterize the potential secreting factors, we carried out mass spectrometric analysis of CM from VP-16-treated responsive (LoVo, SW480) and unresponsive (SW620) cells. These three cell lines exhibited distinct profiles of protein up-regulation and down-regulation (Additional file 2: Tables S1-S3), while a subset of overlapping proteins was found (Fig. 2c). Among them, seven proteins (HMGB2, PRDX3, S100A7, RPS6, RPS25, NT5DC1, EIF3C) were down-regulated in LoVo and SW480 cells, meanwhile only two proteins (CXCL1, LRSAM1) were up-regulated in LoVo and SW480 cells, but not in SW620 cells (Fig. 2d). As CXCL1 is closely associated with metastasis [35], we focused on the CXCL1 in the subsequent studies. After treatment with VP-16 for 0.5 h, levels of CXCL1 were increased in the supernatants of LoVo, SW480 and H446 cells, but not in those of AGS or SW620 cells (Fig. 2e), which validated the results of mass spectrometry. Furthermore, cellular levels of CXCL1 protein were increased in LoVo, SW480, H446 cells after treatment with TI (Fig. 2f). Correlated with increased CXCL1 protein expression, levels of CXCL1 mRNA were also up-regulated in LoVo, SW480 and H446 cells (Fig. 2g). To the contrary, TI’s effects on CXCL1 protein and mRNA expression were marginal in SW620 and AGS cells (Fig. 2f, g). Thus, TI could promote expression and secretion of chemotactic factor CXCL1.

### CXCL1 mediates TI-promoted cancer cell motility

**Fig. 3:**
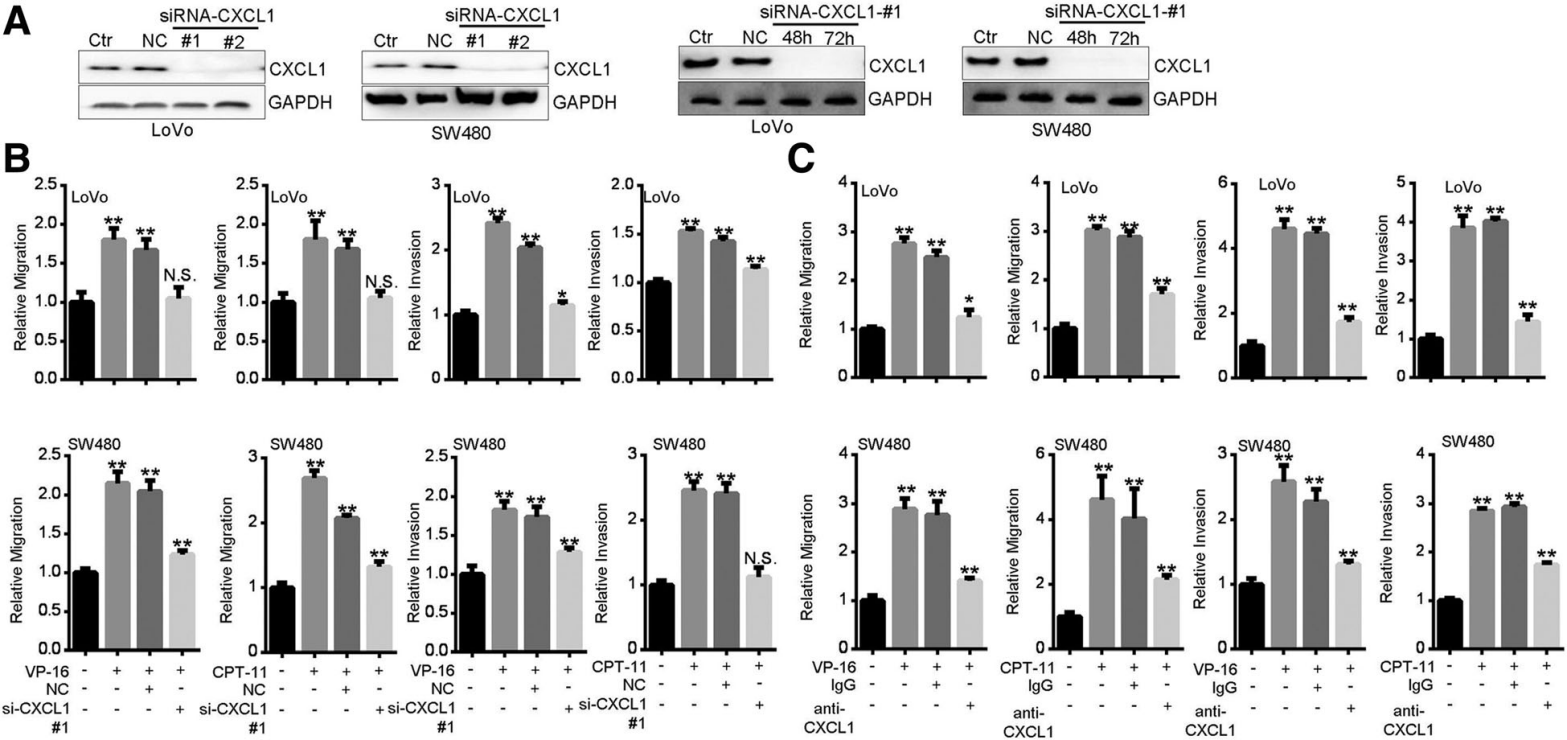
CXCL1 is critical for Topoisomerase inhibitors-promoted cancer cell migration and invasion. **a** Validation of the efficiency of CXCL1 knockdown after transfection with different siRNAs for 48 h (left two panels) or siRNA #1 for 48 and 72 h (right two panels). **b** CXCL1 knockdown prevented TI-promoted motility. After siRNA transfection for 24 h, cells were subjected to migration or invasion assay for 24 h in the presence of VP-16 (20 μM) or CPT-11 (80 μg/ml). **c** Neutralization of CXCL1 prevented TI-promoted motility. Cells were subjected to migration or invasion assay for 24 h in the presence of VP-16 (20 μM) or CPT-11 (80 μg/ml) plus 5 μg/ml of anti-CXCL1 or IgG

To corroborate the role of CXCL1, we used siRNA to silence CXCL1 protein expression (Fig. 3a). After knockdown of CXCL1, VP-16 or CPT-11-promoted migration and invasion were significantly alleviated (Fig. 3b). By using a neutralizing antibody against CXCL1, we also found that VP-16 or CPT-11-promoted cell mobility was greatly prevented (Fig. 3c). These results support the notion that CXCL1 mediates TI-promoted cancer cell mobility.

### JAK2-STAT1 pathway mediates TI-promoted CXCL1 expression and motility

**Fig. 4:**
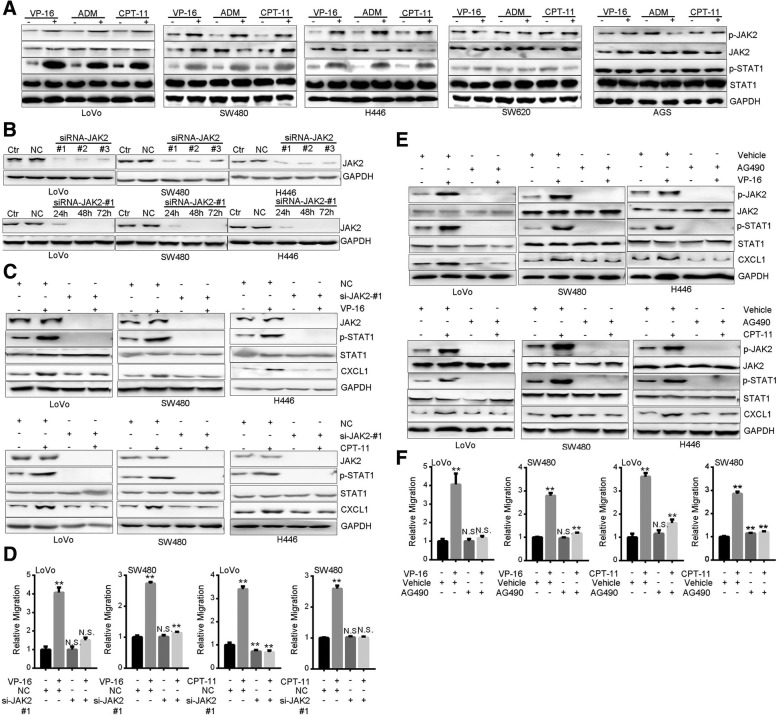
Topoisomerase inhibitors activate JAK2-STAT1 pathway and JAK2 mediates TI-promoted CXCL1 and cell motility. **a** Western blot analysis of JAK2 and STAT1 phosphorylation after treatment with VP-16 (20 μM), CPT-11 (80 μg/ml), or ADM (0.2 μg/ml) for 0.5 h. **b** Validation of the efficiency of JAK2 knockdown after transfection with different siRNAs for 48 h (upper three panels) or siRNA #1 for 24, 48 and 72 h (lower three panels). **c** Western blot analysis of STAT1 phosphorylation and CXCL1 expression in cells transfected with JAK2-specific siRNA for 48 h. Cells were treated with VP-16 (20 μM) or CPT-11 (80 μg/ml) for 0.5 h before harvest. **d** Migration assay of cells transfected with JAK2-specific siRNA. 24 h after transfection, cells were subjected to migration assay for 24 h in the presence of VP-16 (20 μM) or CPT-11 (80 μg/ml). **e** Western blot of JAK2 and STAT1 phosphorylation and CXCL1 expression in cells pretreated with AG490 (40 μM) for 1 h and subsequently treated with VP-16 (20 μM) or CPT-11 (80 μg/ml) for 0.5 h. **f** Migration assay of cells treated with AG490 (40 μM) plus VP-16 (20 μM) or CPT-11 for 24 h

**Fig. 5:**
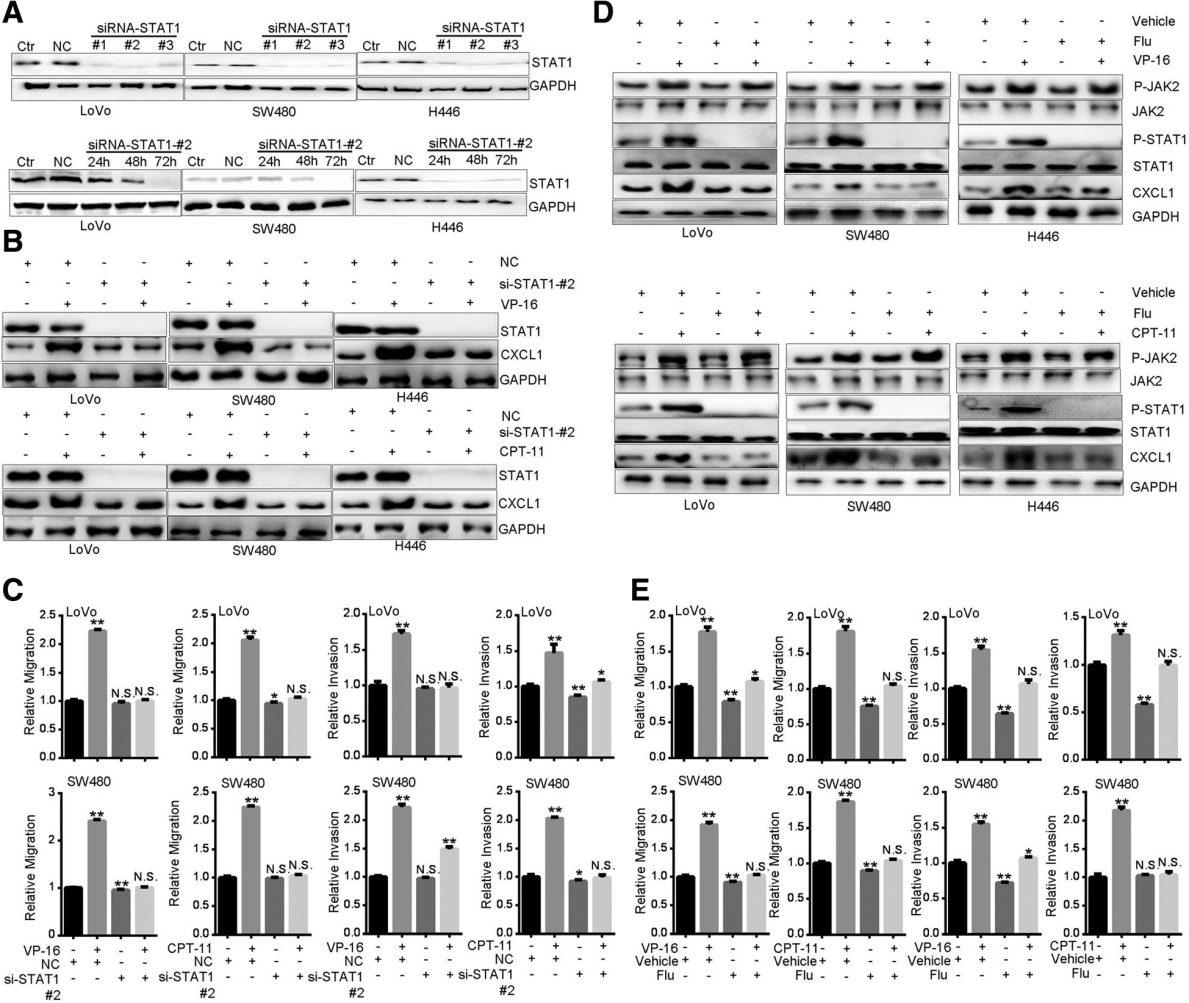
STAT1 mediates Topoisomerase inhibitors-promoted CXCL1 expression and cell motility. **a** Validation of the efficiency of STAT1 knockdown after transfection with different siRNAs for 48 h (upper three panels) or siRNA #2 for 24, 48 and 72 h (lower three panels). **b** Western blot analysis of CXCL1 expression in cells transfected with STAT1-specific siRNA for 48 h. Cells were treated with VP-16 (20 μM) or CPT-11 (80 μg/ml) for 0.5 h before harvest. **c** Migration assay of cells transfected with STAT1-specific siRNA. 24 h after transfection, cells were subjected to migration assay for 24 h in the presence of VP-16 (20 μM) or CPT-11 (80 μg/ml). **d** Western blot of JAK2 and STAT1 phosphorylation and CXCL1 expression in cells pretreated with Fludarabine (100 μM) for 1 h and subsequently treated with VP-16 (20 μM) or CPT-11 (80 μg/ml) for 0.5 h. **e** Migration assay of cells treated with Fludarabine (100 μM) plus VP-16 (20 μM) or CPT-11 (80 μg/ml) for 24 h

Next, we sought to delineate the mechanism underlying TI-induced CXCL1 expression. Now that TI stimulated CXCL1 transcription (Fig. 2g) and JAK2-STAT1 pathway is involved in transactivation of CXCL1 [36], we examined the roles of JAK2 and STAT1. Treatment with TI for 0.5 h strongly increased phosphorylation levels of JAK2 and STAT1 in LoVo, SW480 and H446 cells, but not in SW620 or AGS cells (Fig. 4a). After interfering the expression of JAK2 (Fig. 4b), TI-induced STAT1 phosphorylation and CXCL1 expression were abrogated (Fig. 4c). Meanwhile, TI-promoted migration was inhibited (Fig. 4d). Similarly, by utilizing the JAK2 inhibitor AG490 [37, 38], we found that TI-promoted STAT1 phosphorylation, CXCL1 induction, and cell migration were all blocked (Fig. 4e, f). Additionally, knockdown of STAT1 abolished TI-promoted CXCL1 expression and migration (Fig. 5a-c). In line with these results, pretreatment with Fludarabine (Flu), a specific inhibitor of STAT1 [37, 38], achieved similar effects (Fig. 5d, e). These results suggest that activation of JAK2-STAT1 pathway mediates TI-promoted CXCL1 expression and cell motility.

**Fig. 6:**
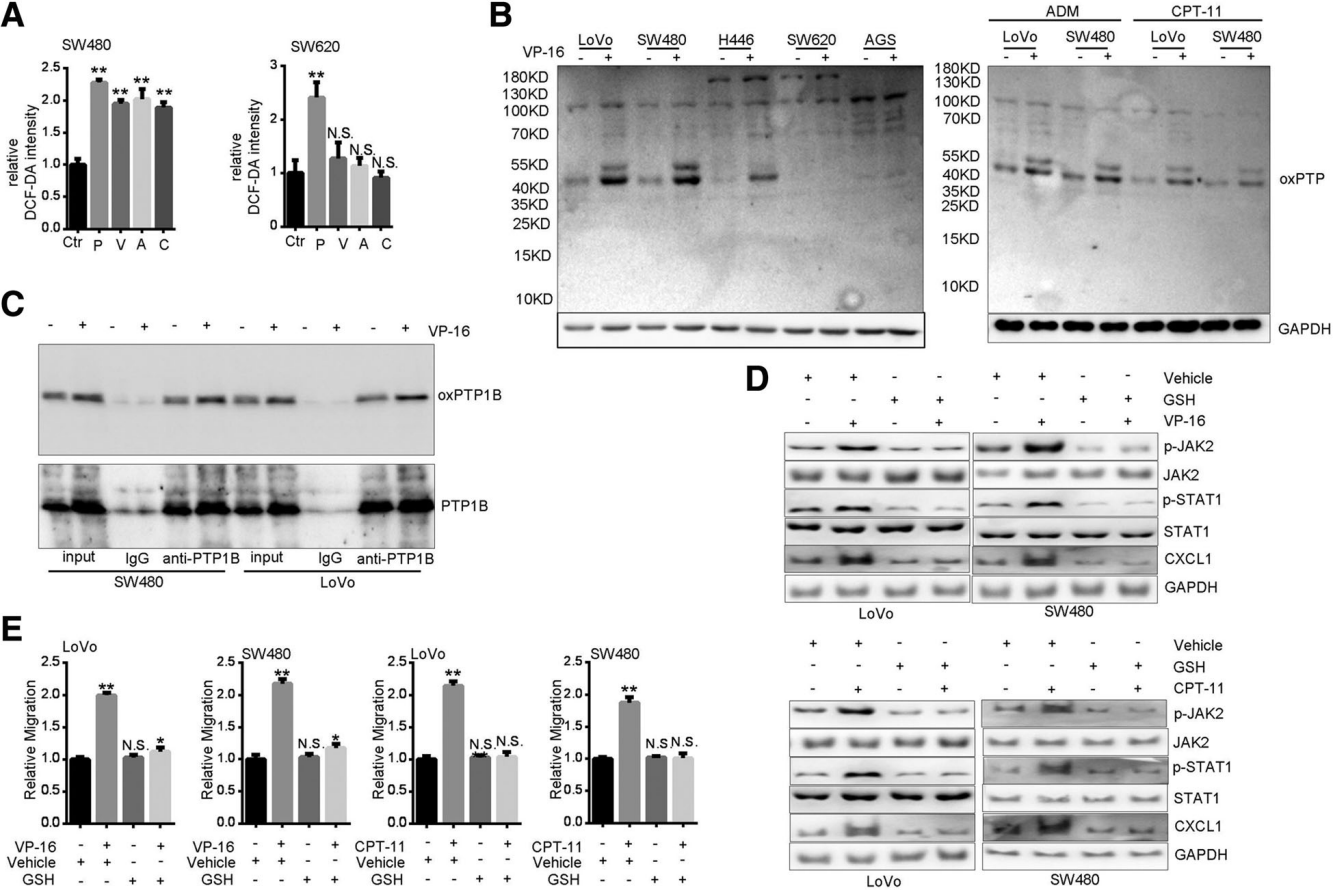
Topoisomerase inhibitors induce JAK2-STAT1-CXCL1 and migration through ROS. **a** Relative DCFH-DA levels in SW480 and SW620 cells treated with VP-16 (V, 20 μM), ADM (A, 0.2 μg/ml), or CPT-11 (C, 80 μg/ml) for 0.5 h. P, a positive control with Rosup H_2_O_2_ (50 μg/ml, 0.5 h). (B) Western blot of oxidized PTPs after treatement with VP-16 (20 μM), ADM (0.2 μg/ml), or CPT-11 (80 μg/ml) for 0.5 h. **c** Confirmation of VP-16-induced PTP1B oxidization. After treatment with VP-16 (20 μM, 0.5 h), cell lysates (200 μg per sample) were immunoprecipitated with 1 μg anti-PTP1B or pre-immune IgG for 12 h. Precipitates and cell lysates (input, 50 μg per sample) were analyzed by Western blot with anti-oxPTP and anti-PTP1B. **d** Western blot of JAK2 and STAT1 phosphorylation and CXCL1 expression in cells pretreated with GSH (10 mM) for 2 h and subsequently treated with VP-16 (20 μM) or CPT-11 (80 μg/ml) for 0.5 h. **e** Migration assay of cells treated with GSH (10 mM) plus VP-16 (20 μM) or CPT-11 for 24 h

### TI activates JAK2-STAT1-CXCL1 pathway through ROS-mediated PTP1B oxidization

Next, we examined the mechanism of TI-promoted JAK2 activation. JAK2 phosphorylation is positively regulated by stimulation with cytokines and growth factors, or negatively regulated by such protein tyrosine phosphatases (PTPs) as PTP1B and TC-PTP [39]. Additionally, JAK2 signaling pathway could be modulated by ROS [40–42]. Interestingly, JAK2 phosphorylation was revealed to be enhanced by ROS-mediated oxidization and inactivation of PTPs [43]. We found that treatment with VP-16, ADM and CPT-11 enhanced the production of ROS in SW480 cells (Fig. 6a), which was consistent with previous findings [44, 45]. However, ROS status in the unresponsive cell line, SW620, was not affected by TI (Fig. 6a). By using an antibody specific for oxidized motif of PTPs (oxPTP) [46], we found that VP-16 markedly increased protein signals at ~ 50 KD only in responsive cell lines (Fig. 6b). Treatment with ADM or CPT-11 also increased oxPTP levels (Fig. 6b). Prompted by the molecular weight of TI-induced oxPTP, we immumoprecipitated PTP1B and performed Western blot with anti-oxPTP. Results confirmed that oxidization of PTP1B was increased by VP-16 (Fig. 6c). With the ROS scavenger GSH, we found that both phosphorylation of JAK2-STAT1 and expression of CXCL1 induced by TI were negated (Fig. 6d). Consistently, TI-promoted cell migration was prevented (Fig. 6e). Collectively, these results indicate that ROS-mediated PTP1B oxidization contributes to TI-stimulated JAK2-STAT1-CXCL1 pathway and motility.

## Discussion

In this study, we demonstrated TI’s capacity in promoting the motility of a subset of cancer cells in vitro and in vivo. This capacity is independent of ATM, NF-κB or cGAS-cGAMP-STING signaling pathways, but relies on TI-boosted expression and secretion of chemokine CXCL1. Importantly, silencing or neutralizing of CXCL1 antagonized TI-promoted motility. We further showed that TI enhanced phosphorylation of JAK2 and STAT1, but inhibition of JAK2 or STAT1 abrogated TI-induced CXCL1 and cell migration. Moreover, TI increased generation of intracellular ROS and promoted oxidation of PTP1B, while GSH reversed TI-induced JAK2-STAT1-CXCL1 signaling and motility.

Although all five cell lines used in this study underwent decreased proliferation upon treatment with TI, their changes in motility were quite distinct. Diminished motility of AGS cells was correlated with its lowered proliferation. LoVo, H446 and SW480 cells exhibited increased motility, whereas motility of SW620 cells was unchanged. In mass spectrometry, the profiles of altered proteins in the CM from VP-16-treated SW620 cells were more complicate (306 down-regulated and 237 up-regulated, as shown in Additional file 2, Table S1). Some up-regulated proteins are known pro-invasive factors, such as Paxillin, Dynein, Talin-1, THBS4, Rab11b, and Transgelin-2. However, certain pro-invasive factors were down-regulated, for example, MMP2, WISP2, CD44, BMP1, Ephrin-A1, and ADAMTS1. Additionally, few anti-invasive factors, e.g. TIMP1 and TIMP2, were also down-regulated. To the contrary, limited regulators of motility were identified in the CM from VP-16-treated SW480 and LoVo cells, and importantly, increased CXCL1 was revealed (Additional file 2: Tables S2-S3). It’s likely that the invasiveness of TI-treated cells was regulated by the counterbalance of pro-invasive and anti-invasive factors, while CXCL1 is a critical determinant. This assumption needs to be further validated by scrutinizing more cancer cell lines as well as clinical samples.

Chemokines have been extensively related to cellular transformation, cancer growth, homing, and metastasis [47]. Chemokines of CXC family were predicted as prognostic biomarkers and possible drug targets in colorectal cancer [48]. CXCL1 was essential for pre-metastatic niche formation and metastasis of colorectal cancer by modulating the microenvironmental pathways [35]. Down-regulation of CXCL1 inhibited liver metastasis of colorectal cancer [49]. Previous studies found that both stroma and cancer cells could secrete CXCL1 in response to chemotherapeutic agents. For example, VP-16 and ADM induced CXCL1 in bone marrow-derived macrophages [50]. In this study, we found enhanced expression and secretion of CXCL1 by a subset of cancer cells treated with TI. The TI-induced CXCL1 secretion from cancer cells may exert its impacts through autocrine signalings to alter the intracellular pathways or through paracrine processes to remold the microenvironmental niches, thereby facilitating cell motility and dissemination. Our results imply that CXCL1 could be a predictor of TI-associated metastasis. CXCL1 could also be a potential target for decreasing the side effects of TI-based treatment, for example, by using the antibody to CXCL1 as a synergistic agent of chemotherapy.

NF-κB [48], MAPK [48], and JAK2-STAT1 [36] signaling pathways were found to regulate CXCL1 expression. JAK-STAT signaling pathway is tightly involved in controlling cell proliferation, differentiation, apoptosis and immune regulation [51]. In some malignant phenotypes, STAT1 could carry out its functions either as an oncoprotein or cancer suppressor in the same cell type, depending on the specific genetic background [51]. Phosphorylation of STAT1 tyrosine moieties by JAK2 kinase was induced rapidly by cytokines and growth factors stimulation, which in turn elevates its activity to transactivate downstream genes [52]. By using gene silencing and specific chemical inhibitors, we showed that TI-induced CXCL1 expression is dependent on JAK2-STAT1 signaling. This regulation is an acute response, since phosphorylation of JAK2-STAT1 and expression of CXCL1 were quickly enhanced after treatment with TI for half an hour. Inhibition of JAK2-STAT1 signaling also counteracted TI-induced cell motility, which supports the possibility of restraining this pathway to prevent TI-induced metastasis. This assumption also needs to be verified in future studies.

Emerging evidence indicates ROS as an additional regulator of JAK2 signaling pathway [40–42]. ROS-mediated JAK2 inhibition by GSH depletion could inhibit cell growth and induce apoptosis in hepatocellular carcinoma cells [40]. Besides, ROS-modulated JAK2 pathway was reported to be associated with gastric cancer progression and apoptosis [41]. Attention had been drawn to PTPs as ROS targets because of the signature motif of this enzyme family [43]. In this study, we found increased ROS after treatment with TI and further showed TI-promoted PTP1B oxidization. Furthermore, ROS chelator GSH abolished TI’s stimulating effects on JAK2-STAT1 phosphorylation, CXCL1 expression as well as the cell motility. Our results support the prospect of ROS intervention as another approach to prevent TI-induced metastasis. A critical issue remained to be addressed is the unaltered ROS status in few cell lines, i.e., SW620 and AGS, which was associated with unaffected JAK2-STAT1-CXCL1 signaling and motility after TI treatment. This could be resolved by comparing the expression profiles of critical enzymes mediating the metabolism of TI.

## Conclusion

We demonstrated that TI promotes the motility of a subset of cancer cells. Up-regulation of JAK2-STAT1-CXCL1 pathway is essential for TI-promoted motility. TI could activate JAK2 through ROS-mediated PTP1B oxidization and inactivation.

## Additional files

Additional file 1: **Figure S1.** Effect of topoisomerase inhibitors on cancer cell proliferation. **Figure S2.** Effect of topoisomerase inhibitors on the motility of SW620 and AGS cells. **Figure S3.** topoisomerase inhibitors-promoted cell migration is independent of ATM, NFκB or cGAS-STING pathway. (DOC 2516 kb)

Additional file 2: **Table S1.** SW620 mass spectra data. **Table S2.** SW480 mass spectra data. **Table S3.** LoVo mass spectra data. (DOC 1293 kb)

## Data Availability

All data generated during this study are included in this published article and its supplementary information files.

## Abbreviations

ADM: Adriamycin
CM: Conditioned medium
CPT-11: Irinotecan
CXCL1: Chemokine (C-X-C motif) ligand 1
DCFH-DA: 2,7-Dichlorodi -hydrofluorescein diacetate
Flu: Fludarabine
GSH: Reduced glutathione
JAK2: Janus kinase 2
PTP1B: Protein Tyrosine Phosphatase 1B
ROS: Reactive oxygen species
SDS-PAGE: Sodium Dodecyl Sulfate–Polyacrylamide Gel Electrophoresis
siRNA: Small interfering RNA
STAT1: Signal transducers and activators of transcription 1
TEAB: Triethylamonium bicarbonate
TI: Topoisomerase inhibitors
TMT: Tandem Mass Tag
VP-16: Etoposide
