# Supplementary material for: Topoisomerase inhibitors promote cancer cell motility via ROS-mediated activation of JAK2-STAT1-CXCL1 pathway
Source: J Exp Clin Cancer Res. 2019 Aug 22;38:370. doi: 10.1186/s13046-019-1353-2 (PMC6704639; doi:10.1186/s13046-019-1353-2)
Supplement: Supplementary file 1 — Figure S1. Effect of topoisomerase inhibitors on cancer cell proliferation. Figure S2. Effect of topoisomerase inhibitors on the motility of SW620 and AGS cells. Figure S3. topoisomerase inhibitors-promoted cell migration is independent of ATM, NFκB or cGAS-STING pathway. (DOC 2516 kb) [file 13046_2019_1353_MOESM1_ESM.doc]

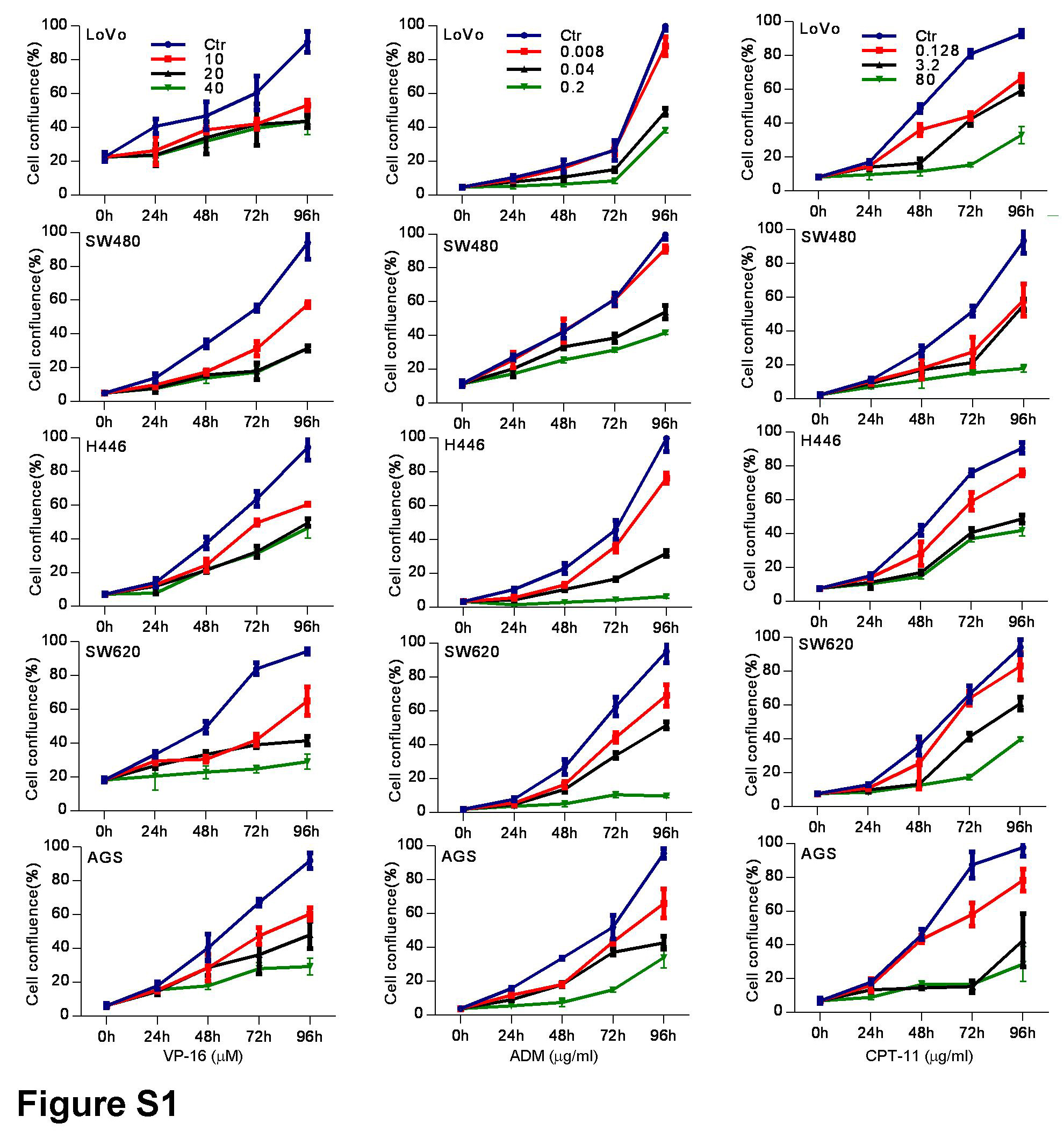


**Supplementary Figure 1.** Effect of topoisomerase inhibitors on cancer cell proliferation.

**
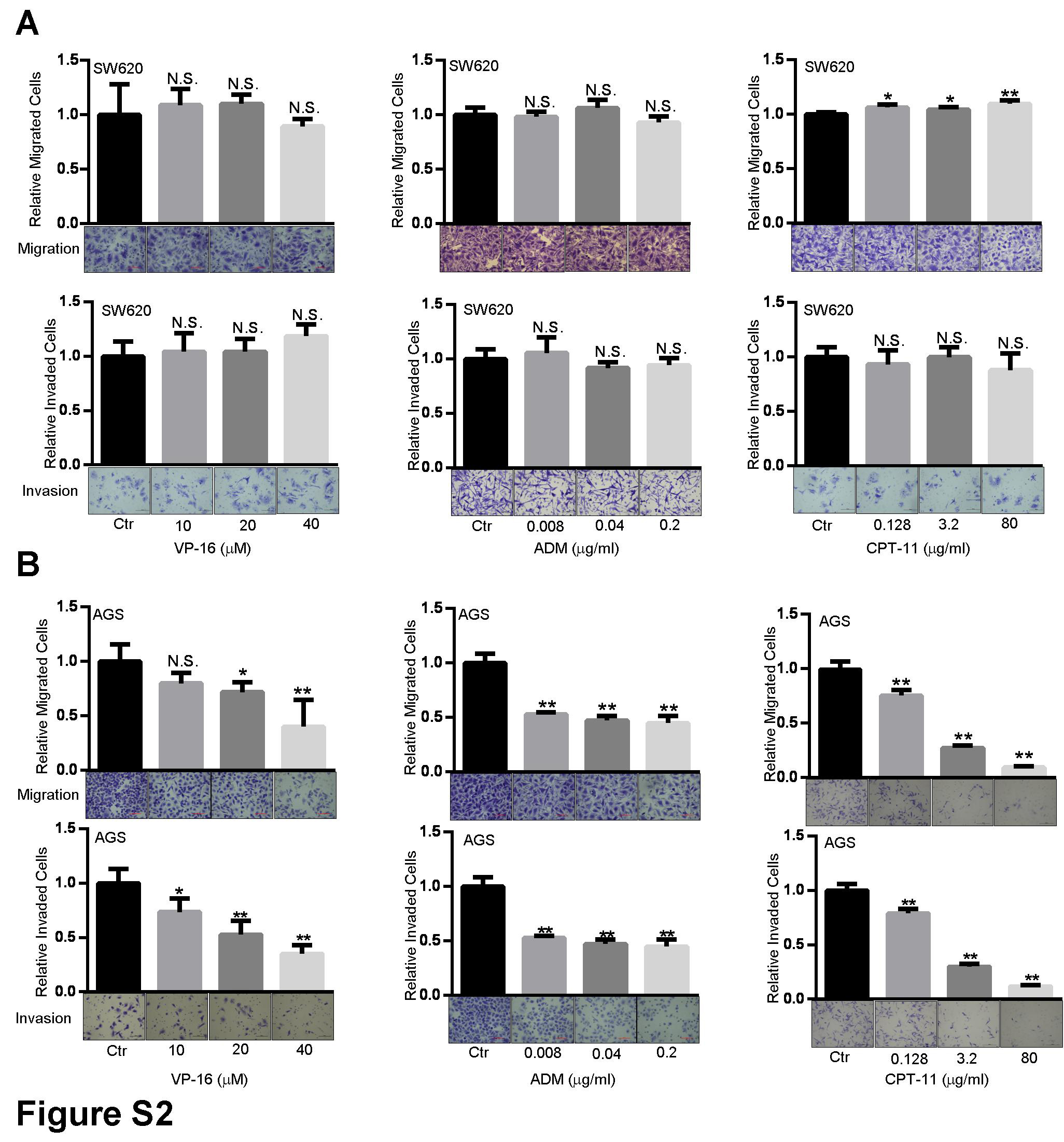
**

**Supplementary Figure 2.** Effect of topoisomerase inhibitors on the motility of SW620 and AGS cells.

SW620 (A) and AGS (B) cells were treated with indicated concentrations of VP-16、ADM or CPT-11 and subjected to migration and invasion assays.

**
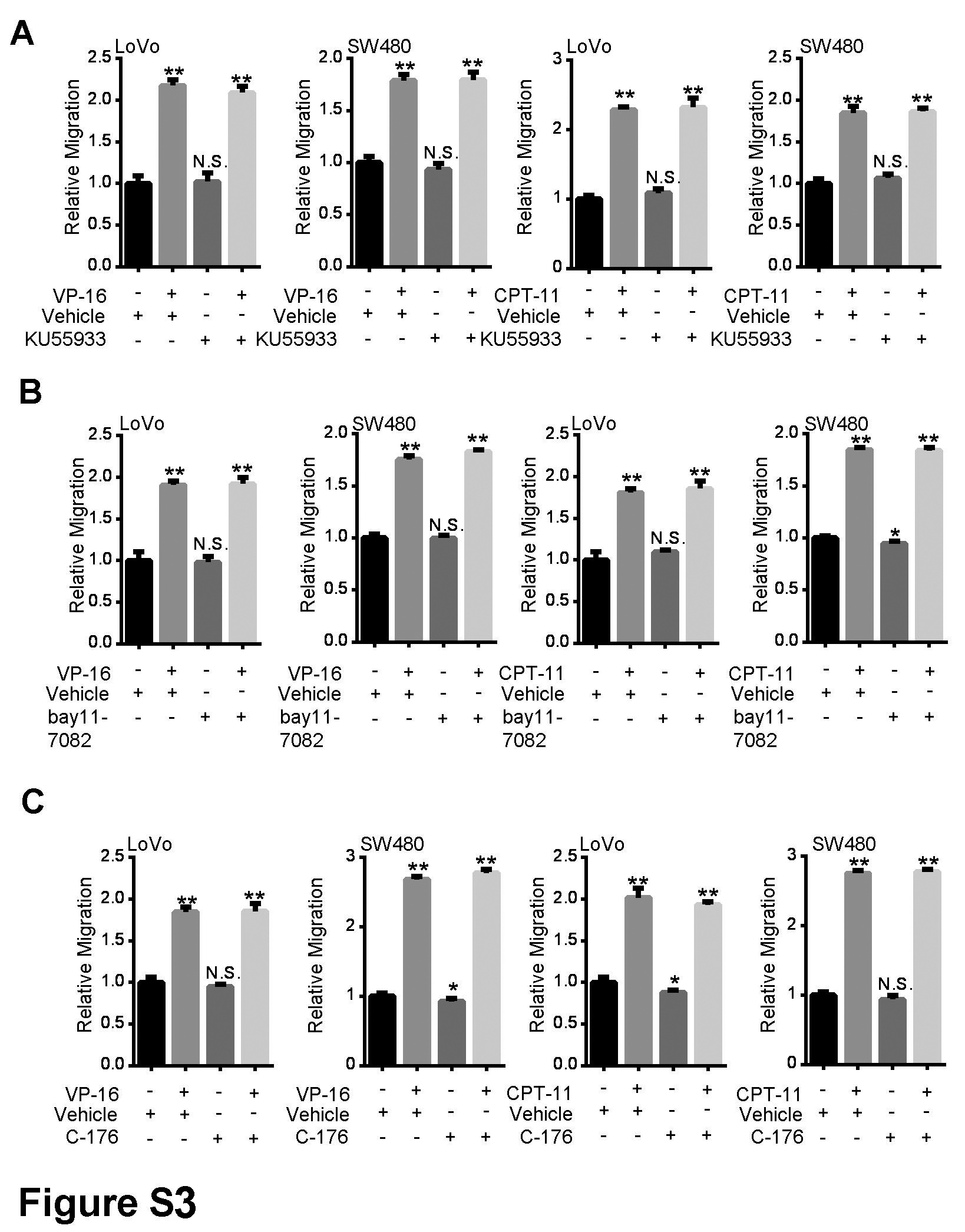
**

**Supplementary Figure 3.** topoisomerase inhibitors-promoted cell migration is independent of ATM, NFκB or cGAS-STING pathway.

(A) Migration of cells treated with ATM inhibitor KU55933 (2 μM) plus VP-16 (20 μM) or CPT-11 (80 μg/ml) for 24 h.

(B) Migration of cells treated with NFκB inhibitore Bay11-7082 (1 μM) plus VP-16 (20 μM) or CPT-11 (80 μg/ml) for 24 h.

(C) Migration of cells treated with STING inhibitor C-176 (0.5 μM) plus VP-16 (20μM) or CPT-11 (80 μg/ml) for 24 h.
