# Supplementary material for: Topoisomerase inhibitors promote cancer cell motility via ROS-mediated activation of JAK2-STAT1-CXCL1 pathway
Source: J Exp Clin Cancer Res. 2019 Aug 22;38:370. doi: 10.1186/s13046-019-1353-2 (PMC6704639; doi:10.1186/s13046-019-1353-2)
Supplement: Supplementary file 2 — Table S1. SW620 mass spectra data. Table S2. SW480 mass spectra data. Table S3. LoVo mass spectra data. (DOC 1293 kb) [file 13046_2019_1353_MOESM2_ESM.doc]

**Table S1. SW620 mass spectra data.**

| **Proten accession** | **Protein description** | **SW620-VP/**  **SW620-C**  **Ratio** | **Regulated**  **Type** | **SW620-VP/**  **SW620-C**  **P value** | **Gene name** |
| --- | --- | --- | --- | --- | --- |
| P26599 | Polypyrimidine tract-binding protein 1 OS=Homo sapiens GN=PTBP1 | 1.795 | Up | 0.000122 | PTBP1 |
| P04114 | Apolipoprotein B-100 OS=Homo sapiens GN=APOB | 1.78 | Up | 0.014037 | APOB |
| P61764 | Syntaxin-binding protein 1 OS=Homo sapiens GN=STXBP1 | 1.775 | Up | 0.044 | STXBP1 |
| Q2M389 | WASH complex subunit SWIP OS=Homo sapiens GN=KIAA1033 | 1.749 | Up | 3.83E-08 | KIAA1033 |
| Q13085 | Acetyl-CoA carboxylase 1 OS=Homo sapiens GN=ACACA | 1.699 | Up | 0.025259 | ACACA |
| Q05639 | Elongation factor 1-alpha 2 OS=Homo sapiens GN=EEF1A2 | 1.697 | Up | 0.008464 | EEF1A2 |
| P48739 | Phosphatidylinositol transfer protein beta isoform OS=Homo sapiens GN=PITPNB | 1.657 | Up | 0.039363 | PITPNB |
| P40227 | T-complex protein 1 subunit zeta OS=Homo sapiens GN=CCT6A | 1.638 | Up | 2.93E-13 | CCT6A |
| P23921 | Ribonucleoside-diphosphate reductase large subunit OS=Homo sapiens GN=RRM1 | 1.6 | Up | 0.000201 | RRM1 |
| O75116 | Rho-associated protein kinase 2 OS=Homo sapiens GN=ROCK2 | 1.59 | Up | 0.000885 | ROCK2 |
| P19623 | Spermidine synthase OS=Homo sapiens GN=SRM | 1.581 | Up | 0.03922 | SRM |
| Q9Y5K5 | Ubiquitin carboxyl-terminal hydrolase isozyme L5 OS=Homo sapiens GN=UCHL5 | 1.575 | Up | 0.000343 | UCHL5 |
| Q92841 | Probable ATP-dependent RNA helicase DDX17 OS=Homo sapiens GN=DDX17 | 1.556 | Up | 1.55E-05 | DDX17 |
| O43488 | Aflatoxin B1 aldehyde reductase member 2 OS=Homo sapiens GN=AKR7A2 | 1.548 | Up | 0.011644 | AKR7A2 |
| P49023 | Paxillin OS=Homo sapiens GN=PXN | 1.544 | Up | 0.035704 | PXN |
| Q9H446 | RWD domain-containing protein 1 OS=Homo sapiens GN=RWDD1 | 1.534 | Up | 0.034916 | RWDD1 |
| P22694 | cAMP-dependent protein kinase catalytic subunit beta OS=Homo sapiens GN=PRKACB | 1.527 | Up | 0.019239 | PRKACB |
| Q96AC1 | Fermitin family homolog 2 OS=Homo sapiens GN=FERMT2 | 1.523 | Up | 0.000238 | FERMT2 |
| Q01433 | AMP deaminase 2 OS=Homo sapiens GN=AMPD2 | 1.509 | Up | 0.001505 | AMPD2 |
| P50991 | T-complex protein 1 subunit delta OS=Homo sapiens GN=CCT4 | 1.509 | Up | 5.91E-05 | CCT4 |
| P46063 | ATP-dependent DNA helicase Q1 OS=Homo sapiens GN=RECQL | 1.504 | Up | 0.018664 | RECQL |
| Q99832 | T-complex protein 1 subunit eta OS=Homo sapiens GN=CCT7 | 1.503 | Up | 0.00024 | CCT7 |
| P11413 | Glucose-6-phosphate 1-dehydrogenase OS=Homo sapiens GN=G6PD | 1.499 | Up | 1.16E-06 | G6PD |
| P20073 | Annexin A7 OS=Homo sapiens GN=ANXA7 | 1.487 | Up | 0.004362 | ANXA7 |
| Q96C19 | EF-hand domain-containing protein D2 OS=Homo sapiens GN=EFHD2 | 1.481 | Up | 0.001544 | EFHD2 |
| Q9C0B1 | Alpha-ketoglutarate-dependent dioxygenase FTO OS=Homo sapiens GN=FTO | 1.479 | Up | 0.002463 | FTO |
| Q9HB71 | Calcyclin-binding protein OS=Homo sapiens GN=CACYBP | 1.478 | Up | 0.000304 | CACYBP |
| Q96TA1 | Niban-like protein 1 OS=Homo sapiens GN=FAM129B | 1.473 | Up | 2.7E-08 | FAM129B |
| P62495 | Eukaryotic peptide chain release factor subunit 1 OS=Homo sapiens GN=ETF1 | 1.472 | Up | 0.000142 | ETF1 |
| Q16539 | Mitogen-activated protein kinase 14 OS=Homo sapiens GN=MAPK14 | 1.47 | Up | 0.043276 | MAPK14 |
| Q14204 | Cytoplasmic dynein 1 heavy chain 1 OS=Homo sapiens GN=DYNC1H1 | 1.467 | Up | 1.9E-12 | DYNC1H1 |
| P26640 | Valine--tRNA ligase OS=Homo sapiens GN=VARS | 1.466 | Up | 0.000603 | VARS |
| Q99536 | Synaptic vesicle membrane protein VAT-1 homolog OS=Homo sapiens GN=VAT1 | 1.463 | Up | 0.011936 | VAT1 |
| P63010 | AP-2 complex subunit beta OS=Homo sapiens GN=AP2B1 | 1.458 | Up | 0.002756 | AP2B1 |
| P22234 | Multifunctional protein ADE2 OS=Homo sapiens GN=PAICS | 1.457 | Up | 0.021284 | PAICS |
| Q9UHD1 | Cysteine and histidine-rich domain-containing protein 1 OS=Homo sapiens GN=CHORDC1 | 1.45 | Up | 0.00758 | CHORDC1 |
| P61088 | Ubiquitin-conjugating enzyme E2 N OS=Homo sapiens GN=UBE2N | 1.447 | Up | 7.78E-08 | UBE2N |
| O14980 | Exportin-1 OS=Homo sapiens GN=XPO1 | 1.447 | Up | 7.57E-08 | XPO1 |
| P31483 | Nucleolysin TIA-1 isoform p40 OS=Homo sapiens GN=TIA1 | 1.443 | Up | 0.009017 | TIA1 |
| Q9Y490 | Talin-1 OS=Homo sapiens GN=TLN1 | 1.439 | Up | 1.62E-12 | TLN1 |
| P49368 | T-complex protein 1 subunit gamma OS=Homo sapiens GN=CCT3 | 1.436 | Up | 6.92E-09 | CCT3 |
| Q15018 | BRISC complex subunit Abro1 OS=Homo sapiens GN=FAM175B | 1.431 | Up | 0.04198 | FAM175B |
| P49327 | Fatty acid synthase OS=Homo sapiens GN=FASN | 1.429 | Up | 4.33E-15 | FASN |
| Q9NTZ6 | RNA-binding protein 12 OS=Homo sapiens GN=RBM12 | 1.428 | Up | 0.020859 | RBM12 |
| P34896 | Serine hydroxymethyltransferase, cytosolic OS=Homo sapiens GN=SHMT1 | 1.425 | Up | 0.001239 | SHMT1 |
| P22392 | Nucleoside diphosphate kinase B OS=Homo sapiens GN=NME2 | 1.424 | Up | 0.005843 | NME2 |
| P22314 | Ubiquitin-like modifier-activating enzyme 1 OS=Homo sapiens GN=UBA1 | 1.423 | Up | 1.63E-12 | UBA1 |
| P04083 | Annexin A1 OS=Homo sapiens GN=ANXA1 | 1.423 | Up | 8.25E-07 | ANXA1 |
| O15144 | Actin-related protein 2/3 complex subunit 2 OS=Homo sapiens GN=ARPC2 | 1.422 | Up | 0.000104 | ARPC2 |
| O15305 | Phosphomannomutase 2 OS=Homo sapiens GN=PMM2 | 1.422 | Up | 0.010284 | PMM2 |
| O15372 | Eukaryotic translation initiation factor 3 subunit H OS=Homo sapiens GN=EIF3H | 1.42 | Up | 0.00574 | EIF3H |
| P08865 | 40S ribosomal protein SA OS=Homo sapiens GN=RPSA | 1.42 | Up | 0.008859 | RPSA |
| Q99829 | Copine-1 OS=Homo sapiens GN=CPNE1 | 1.419 | Up | 0.003058 | CPNE1 |
| P60842 | Eukaryotic initiation factor 4A-I OS=Homo sapiens GN=EIF4A1 | 1.411 | Up | 0.035683 | EIF4A1 |
| P14618 | Pyruvate kinase PKM OS=Homo sapiens GN=PKM | 1.409 | Up | 5.55E-16 | PKM |
| P63000 | Ras-related C3 botulinum toxin substrate 1 OS=Homo sapiens GN=RAC1 | 1.408 | Up | 0.008556 | RAC1 |
| P17987 | T-complex protein 1 subunit alpha OS=Homo sapiens GN=TCP1 | 1.408 | Up | 7.8E-08 | TCP1 |
| Q9BXJ9 | N-alpha-acetyltransferase 15, NatA auxiliary subunit OS=Homo sapiens GN=NAA15 | 1.408 | Up | 0.000157 | NAA15 |
| P49419 | Alpha-aminoadipic semialdehyde dehydrogenase OS=Homo sapiens GN=ALDH7A1 | 1.408 | Up | 0.006381 | ALDH7A1 |
| P62136 | Serine/threonine-protein phosphatase PP1-alpha catalytic subunit OS=Homo sapiens GN=PPP1CA | 1.404 | Up | 0.020398 | PPP1CA |
| P55209 | Nucleosome assembly protein 1-like 1 OS=Homo sapiens GN=NAP1L1 | 1.403 | Up | 0.000122 | NAP1L1 |
| P15170 | Eukaryotic peptide chain release factor GTP-binding subunit ERF3A OS=Homo sapiens GN=GSPT1 | 1.401 | Up | 0.003524 | GSPT1 |
| P35443 | Thrombospondin-4 OS=Homo sapiens GN=THBS4 | 1.399 | Up | 0.004997 | THBS4 |
| P36952 | Serpin B5 OS=Homo sapiens GN=SERPINB5 | 1.399 | Up | 0.033896 | SERPINB5 |
| Q92905 | COP9 signalosome complex subunit 5 OS=Homo sapiens GN=COPS5 | 1.395 | Up | 0.000558 | COPS5 |
| P53396 | ATP-citrate synthase OS=Homo sapiens GN=ACLY | 1.392 | Up | 1.11E-16 | ACLY |
| Q04446 | 1,4-alpha-glucan-branching enzyme OS=Homo sapiens GN=GBE1 | 1.389 | Up | 0.011664 | GBE1 |
| P62829 | 60S ribosomal protein L23 OS=Homo sapiens GN=RPL23 | 1.385 | Up | 0.017545 | RPL23 |
| Q12792 | Twinfilin-1 OS=Homo sapiens GN=TWF1 | 1.381 | Up | 0.016104 | TWF1 |
| Q6PGP7 | Tetratricopeptide repeat protein 37 OS=Homo sapiens GN=TTC37 | 1.379 | Up | 0.000555 | TTC37 |
| Q9Y3A5 | Ribosome maturation protein SBDS OS=Homo sapiens GN=SBDS | 1.377 | Up | 0.00012 | SBDS |
| O15143 | Actin-related protein 2/3 complex subunit 1B OS=Homo sapiens GN=ARPC1B | 1.377 | Up | 0.000319 | ARPC1B |
| P49736 | DNA replication licensing factor MCM2 OS=Homo sapiens GN=MCM2 | 1.369 | Up | 0.003235 | MCM2 |
| O14929 | Histone acetyltransferase type B catalytic subunit OS=Homo sapiens GN=HAT1 | 1.369 | Up | 0.000896 | HAT1 |
| P47756 | F-actin-capping protein subunit beta OS=Homo sapiens GN=CAPZB | 1.367 | Up | 6.15E-05 | CAPZB |
| Q02790 | Peptidyl-prolyl cis-trans isomerase FKBP4 OS=Homo sapiens GN=FKBP4 | 1.363 | Up | 5.84E-12 | FKBP4 |
| Q96RS6 | NudC domain-containing protein 1 OS=Homo sapiens GN=NUDCD1 | 1.362 | Up | 0.008256 | NUDCD1 |
| P23396 | 40S ribosomal protein S3 OS=Homo sapiens GN=RPS3 | 1.362 | Up | 3.62E-07 | RPS3 |
| Q14232 | Translation initiation factor eIF-2B subunit alpha OS=Homo sapiens GN=EIF2B1 | 1.362 | Up | 0.001898 | EIF2B1 |
| Q14566 | DNA replication licensing factor MCM6 OS=Homo sapiens GN=MCM6 | 1.361 | Up | 1.89E-08 | MCM6 |
| Q9NZL4 | Hsp70-binding protein 1 OS=Homo sapiens GN=HSPBP1 | 1.361 | Up | 0.011056 | HSPBP1 |
| Q9H773 | dCTP pyrophosphatase 1 OS=Homo sapiens GN=DCTPP1 | 1.36 | Up | 0.021996 | DCTPP1 |
| O15212 | Prefoldin subunit 6 OS=Homo sapiens GN=PFDN6 | 1.36 | Up | 0.0017 | PFDN6 |
| Q7KZF4 | Staphylococcal nuclease domain-containing protein 1 OS=Homo sapiens GN=SND1 | 1.359 | Up | 1.58E-06 | SND1 |
| P46940 | Ras GTPase-activating-like protein IQGAP1 OS=Homo sapiens GN=IQGAP1 | 1.358 | Up | 2.51E-10 | IQGAP1 |
| O60701 | UDP-glucose 6-dehydrogenase OS=Homo sapiens GN=UGDH | 1.355 | Up | 0.006522 | UGDH |
| Q9H074 | Polyadenylate-binding protein-interacting protein 1 OS=Homo sapiens GN=PAIP1 | 1.355 | Up | 0.002637 | PAIP1 |
| Q9NSD9 | Phenylalanine--tRNA ligase beta subunit OS=Homo sapiens GN=FARSB | 1.354 | Up | 0.001517 | FARSB |
| P36405 | ADP-ribosylation factor-like protein 3 OS=Homo sapiens GN=ARL3 | 1.353 | Up | 0.025578 | ARL3 |
| Q99733 | Nucleosome assembly protein 1-like 4 OS=Homo sapiens GN=NAP1L4 | 1.351 | Up | 0.013824 | NAP1L4 |
| Q15417 | Calponin-3 OS=Homo sapiens GN=CNN3 | 1.351 | Up | 0.014236 | CNN3 |
| Q8WVM8 | Sec1 family domain-containing protein 1 OS=Homo sapiens GN=SCFD1 | 1.348 | Up | 0.009563 | SCFD1 |
| P11940 | Polyadenylate-binding protein 1 OS=Homo sapiens GN=PABPC1 | 1.348 | Up | 0.002296 | PABPC1 |
| Q3LXA3 | Triokinase/FMN cyclase OS=Homo sapiens GN=TKFC | 1.348 | Up | 2.23E-05 | TKFC |
| P54578 | Ubiquitin carboxyl-terminal hydrolase 14 OS=Homo sapiens GN=USP14 | 1.347 | Up | 0.00056 | USP14 |
| Q06124 | Tyrosine-protein phosphatase non-receptor type 11 OS=Homo sapiens GN=PTPN11 | 1.346 | Up | 9.79E-05 | PTPN11 |
| Q13426 | DNA repair protein XRCC4 OS=Homo sapiens GN=XRCC4 | 1.346 | Up | 0.010663 | XRCC4 |
| Q13838 | Spliceosome RNA helicase DDX39B OS=Homo sapiens GN=DDX39B | 1.346 | Up | 0.0041 | DDX39B |
| P13797 | Plastin-3 OS=Homo sapiens GN=PLS3 | 1.345 | Up | 0.001244 | PLS3 |
| Q9Y3C8 | Ubiquitin-fold modifier-conjugating enzyme 1 OS=Homo sapiens GN=UFC1 | 1.343 | Up | 0.004185 | UFC1 |
| Q13126 | S-methyl-5'-thioadenosine phosphorylase OS=Homo sapiens GN=MTAP | 1.342 | Up | 0.019124 | MTAP |
| Q96P70 | Importin-9 OS=Homo sapiens GN=IPO9 | 1.34 | Up | 0.009163 | IPO9 |
| P84077 | ADP-ribosylation factor 1 OS=Homo sapiens GN=ARF1 | 1.339 | Up | 0.049319 | ARF1 |
| Q53GS9 | U4/U6.U5 tri-snRNP-associated protein 2 OS=Homo sapiens GN=USP39 | 1.338 | Up | 0.00342 | USP39 |
| P50990 | T-complex protein 1 subunit theta OS=Homo sapiens GN=CCT8 | 1.337 | Up | 2.39E-10 | CCT8 |
| Q13283 | Ras GTPase-activating protein-binding protein 1 OS=Homo sapiens GN=G3BP1 | 1.336 | Up | 2.16E-07 | G3BP1 |
| Q16851 | UTP--glucose-1-phosphate uridylyltransferase OS=Homo sapiens GN=UGP2 | 1.335 | Up | 0.003044 | UGP2 |
| P41252 | Isoleucine--tRNA ligase, cytoplasmic OS=Homo sapiens GN=IARS | 1.333 | Up | 0.003701 | IARS |
| Q9Y262 | Eukaryotic translation initiation factor 3 subunit L OS=Homo sapiens GN=EIF3L | 1.333 | Up | 2.35E-05 | EIF3L |
| Q9BR76 | Coronin-1B OS=Homo sapiens GN=CORO1B | 1.332 | Up | 0.01724 | CORO1B |
| Q5VYK3 | Proteasome-associated protein ECM29 homolog OS=Homo sapiens GN=ECM29 | 1.331 | Up | 0.001581 | ECM29 |
| Q15181 | Inorganic pyrophosphatase OS=Homo sapiens GN=PPA1 | 1.33 | Up | 1.74E-05 | PPA1 |
| Q7L2H7 | Eukaryotic translation initiation factor 3 subunit M OS=Homo sapiens GN=EIF3M | 1.33 | Up | 0.004584 | EIF3M |
| Q99873 | Protein arginine N-methyltransferase 1 OS=Homo sapiens GN=PRMT1 | 1.33 | Up | 0.002039 | PRMT1 |
| P41091 | Eukaryotic translation initiation factor 2 subunit 3 OS=Homo sapiens GN=EIF2S3 | 1.328 | Up | 0.015183 | EIF2S3 |
| Q15365 | Poly(rC)-binding protein 1 OS=Homo sapiens GN=PCBP1 | 1.328 | Up | 0.028681 | PCBP1 |
| Q15019 | Septin-2 OS=Homo sapiens GN=SEPT2 | 1.324 | Up | 2.35E-06 | SEPT2 |
| Q16222 | UDP-N-acetylhexosamine pyrophosphorylase OS=Homo sapiens GN=UAP1 | 1.322 | Up | 0.025638 | UAP1 |
| O14744 | Protein arginine N-methyltransferase 5 OS=Homo sapiens GN=PRMT5 | 1.321 | Up | 0.001276 | PRMT5 |
| P13639 | Elongation factor 2 OS=Homo sapiens GN=EEF2 | 1.321 | Up | 3.52E-14 | EEF2 |
| P60228 | Eukaryotic translation initiation factor 3 subunit E OS=Homo sapiens GN=EIF3E | 1.317 | Up | 4.24E-05 | EIF3E |
| P37802 | Transgelin-2 OS=Homo sapiens GN=TAGLN2 | 1.314 | Up | 1.78E-05 | TAGLN2 |
| P49407 | Beta-arrestin-1 OS=Homo sapiens GN=ARRB1 | 1.314 | Up | 0.026298 | ARRB1 |
| P55060 | Exportin-2 OS=Homo sapiens GN=CSE1L | 1.314 | Up | 5.84E-05 | CSE1L |
| P55884 | Eukaryotic translation initiation factor 3 subunit B OS=Homo sapiens GN=EIF3B | 1.313 | Up | 0.003438 | EIF3B |
| P18206 | Vinculin OS=Homo sapiens GN=VCL | 1.311 | Up | 1.62E-12 | VCL |
| Q92499 | ATP-dependent RNA helicase DDX1 OS=Homo sapiens GN=DDX1 | 1.309 | Up | 0.000704 | DDX1 |
| O14979 | Heterogeneous nuclear ribonucleoprotein D-like OS=Homo sapiens GN=HNRNPDL | 1.309 | Up | 0.007262 | HNRNPDL |
| P54920 | Alpha-soluble NSF attachment protein OS=Homo sapiens GN=NAPA | 1.307 | Up | 0.036656 | NAPA |
| P59998 | Actin-related protein 2/3 complex subunit 4 OS=Homo sapiens GN=ARPC4 | 1.305 | Up | 0.000636 | ARPC4 |
| P12081 | Histidine--tRNA ligase, cytoplasmic OS=Homo sapiens GN=HARS | 1.303 | Up | 6.97E-09 | HARS |
| Q9UHY1 | Nuclear receptor-binding protein OS=Homo sapiens GN=NRBP1 | 1.303 | Up | 0.030642 | NRBP1 |
| O75534 | Cold shock domain-containing protein E1 OS=Homo sapiens GN=CSDE1 | 1.301 | Up | 0.010381 | CSDE1 |
| Q92616 | eIF-2-alpha kinase activator GCN1 OS=Homo sapiens GN=GCN1 | 1.301 | Up | 0.001421 | GCN1 |
| Q15907 | Ras-related protein Rab-11B OS=Homo sapiens GN=RAB11B | 1.3 | Up | 0.004004 | RAB11B |
| Q8WUM4 | Programmed cell death 6-interacting protein OS=Homo sapiens GN=PDCD6IP | 1.299 | Up | 4.39E-05 | PDCD6IP |
| Q15046 | Lysine--tRNA ligase OS=Homo sapiens GN=KARS | 1.299 | Up | 4.11E-06 | KARS |
| P40121 | Macrophage-capping protein OS=Homo sapiens GN=CAPG | 1.299 | Up | 0.000279 | CAPG |
| A0MZ66 | Shootin-1 OS=Homo sapiens GN=SHTN1 | 1.298 | Up | 0.001821 | SHTN1 |
| P53992 | Protein transport protein Sec24C OS=Homo sapiens GN=SEC24C | 1.298 | Up | 0.000599 | SEC24C |
| Q01518 | Adenylyl cyclase-associated protein 1 OS=Homo sapiens GN=CAP1 | 1.296 | Up | 6.05E-09 | CAP1 |
| Q9BTE3 | Mini-chromosome maintenance complex-binding protein OS=Homo sapiens GN=MCMBP | 1.296 | Up | 0.019037 | MCMBP |
| P27708 | CAD protein OS=Homo sapiens GN=CAD | 1.289 | Up | 2.8E-06 | CAD |
| Q4G0F5 | Vacuolar protein sorting-associated protein 26B OS=Homo sapiens GN=VPS26B | 1.288 | Up | 0.00648 | VPS26B |
| Q9UQE7 | Structural maintenance of chromosomes protein 3 OS=Homo sapiens GN=SMC3 | 1.287 | Up | 0.006982 | SMC3 |
| Q9Y5K6 | CD2-associated protein OS=Homo sapiens GN=CD2AP | 1.285 | Up | 0.0182 | CD2AP |
| P78371 | T-complex protein 1 subunit beta OS=Homo sapiens GN=CCT2 | 1.284 | Up | 1.49E-07 | CCT2 |
| P07437 | Tubulin beta chain OS=Homo sapiens GN=TUBB | 1.283 | Up | 0.011901 | TUBB |
| O60502 | Protein O-GlcNAcase OS=Homo sapiens GN=MGEA5 | 1.282 | Up | 0.01162 | MGEA5 |
| Q8N1G4 | Leucine-rich repeat-containing protein 47 OS=Homo sapiens GN=LRRC47 | 1.282 | Up | 0.001603 | LRRC47 |
| P08238 | Heat shock protein HSP 90-beta OS=Homo sapiens GN=HSP90AB1 | 1.281 | Up | 2.74E-14 | HSP90AB1 |
| P48643 | T-complex protein 1 subunit epsilon OS=Homo sapiens GN=CCT5 | 1.281 | Up | 8.88E-16 | CCT5 |
| Q15436 | Protein transport protein Sec23A OS=Homo sapiens GN=SEC23A | 1.28 | Up | 0.042922 | SEC23A |
| O60664 | Perilipin-3 OS=Homo sapiens GN=PLIN3 | 1.28 | Up | 0.0426 | PLIN3 |
| P04632 | Calpain small subunit 1 OS=Homo sapiens GN=CAPNS1 | 1.28 | Up | 0.003604 | CAPNS1 |
| P05198 | Eukaryotic translation initiation factor 2 subunit 1 OS=Homo sapiens GN=EIF2S1 | 1.278 | Up | 0.00074 | EIF2S1 |
| Q07960 | Rho GTPase-activating protein 1 OS=Homo sapiens GN=ARHGAP1 | 1.277 | Up | 0.047139 | ARHGAP1 |
| P49915 | GMP synthase [glutamine-hydrolyzing] OS=Homo sapiens GN=GMPS | 1.275 | Up | 1.86E-05 | GMPS |
| Q9HC35 | Echinoderm microtubule-associated protein-like 4 OS=Homo sapiens GN=EML4 | 1.275 | Up | 0.002437 | EML4 |
| P62826 | GTP-binding nuclear protein Ran OS=Homo sapiens GN=RAN | 1.274 | Up | 4.1E-08 | RAN |
| Q15437 | Protein transport protein Sec23B OS=Homo sapiens GN=SEC23B | 1.272 | Up | 0.02904 | SEC23B |
| Q7Z6Z7 | E3 ubiquitin-protein ligase HUWE1 OS=Homo sapiens GN=HUWE1 | 1.272 | Up | 0.002981 | HUWE1 |
| P49773 | Histidine triad nucleotide-binding protein 1 OS=Homo sapiens GN=HINT1 | 1.272 | Up | 0.002505 | HINT1 |
| P27694 | Replication protein A 70 kDa DNA-binding subunit OS=Homo sapiens GN=RPA1 | 1.264 | Up | 0.003299 | RPA1 |
| Q16181 | Septin-7 OS=Homo sapiens GN=SEPT7 | 1.263 | Up | 0.005544 | SEPT7 |
| Q8TEA8 | D-tyrosyl-tRNA(Tyr) deacylase 1 OS=Homo sapiens GN=DTD1 | 1.262 | Up | 0.035457 | DTD1 |
| Q92973 | Transportin-1 OS=Homo sapiens GN=TNPO1 | 1.262 | Up | 3.77E-05 | TNPO1 |
| P56192 | Methionine--tRNA ligase, cytoplasmic OS=Homo sapiens GN=MARS | 1.262 | Up | 0.000376 | MARS |
| P49588 | Alanine--tRNA ligase, cytoplasmic OS=Homo sapiens GN=AARS | 1.261 | Up | 0.009381 | AARS |
| P47897 | Glutamine--tRNA ligase OS=Homo sapiens GN=QARS | 1.26 | Up | 0.000179 | QARS |
| Q9Y263 | Phospholipase A-2-activating protein OS=Homo sapiens GN=PLAA | 1.259 | Up | 0.026703 | PLAA |
| P14735 | Insulin-degrading enzyme OS=Homo sapiens GN=IDE | 1.259 | Up | 3.59E-05 | IDE |
| O00232 | 26S proteasome non-ATPase regulatory subunit 12 OS=Homo sapiens GN=PSMD12 | 1.257 | Up | 0.000335 | PSMD12 |
| P26196 | Probable ATP-dependent RNA helicase DDX6 OS=Homo sapiens GN=DDX6 | 1.257 | Up | 0.019923 | DDX6 |
| P14866 | Heterogeneous nuclear ribonucleoprotein L OS=Homo sapiens GN=HNRNPL | 1.257 | Up | 0.002638 | HNRNPL |
| Q9Y265 | RuvB-like 1 OS=Homo sapiens GN=RUVBL1 | 1.255 | Up | 0.000304 | RUVBL1 |
| P62913 | 60S ribosomal protein L11 OS=Homo sapiens GN=RPL11 | 1.254 | Up | 0.000215 | RPL11 |
| Q93009 | Ubiquitin carboxyl-terminal hydrolase 7 OS=Homo sapiens GN=USP7 | 1.253 | Up | 0.000595 | USP7 |
| P33991 | DNA replication licensing factor MCM4 OS=Homo sapiens GN=MCM4 | 1.252 | Up | 0.020119 | MCM4 |
| P09874 | Poly [ADP-ribose] polymerase 1 OS=Homo sapiens GN=PARP1 | 1.251 | Up | 0.00832 | PARP1 |
| O95433 | Activator of 90 kDa heat shock protein ATPase homolog 1 OS=Homo sapiens GN=AHSA1 | 1.25 | Up | 0.000362 | AHSA1 |
| Q9NTK5 | Obg-like ATPase 1 OS=Homo sapiens GN=OLA1 | 1.25 | Up | 0.000136 | OLA1 |
| Q09161 | Nuclear cap-binding protein subunit 1 OS=Homo sapiens GN=NCBP1 | 1.249 | Up | 0.001784 | NCBP1 |
| O43776 | Asparagine--tRNA ligase, cytoplasmic OS=Homo sapiens GN=NARS | 1.248 | Up | 7.31E-07 | NARS |
| O14776 | Transcription elongation regulator 1 OS=Homo sapiens GN=TCERG1 | 1.248 | Up | 0.002101 | TCERG1 |
| Q9BRX5 | DNA replication complex GINS protein PSF3 OS=Homo sapiens GN=GINS3 | 1.246 | Up | 0.004557 | GINS3 |
| P17980 | 26S protease regulatory subunit 6A OS=Homo sapiens GN=PSMC3 | 1.245 | Up | 0.02644 | PSMC3 |
| Q06210 | Glutamine--fructose-6-phosphate aminotransferase [isomerizing] 1 OS=Homo sapiens GN=GFPT1 | 1.244 | Up | 0.041845 | GFPT1 |
| Q96Q11 | CCA tRNA nucleotidyltransferase 1, mitochondrial OS=Homo sapiens GN=TRNT1 | 1.244 | Up | 0.047203 | TRNT1 |
| P12956 | X-ray repair cross-complementing protein 6 OS=Homo sapiens GN=XRCC6 | 1.243 | Up | 1.04E-06 | XRCC6 |
| P51580 | Thiopurine S-methyltransferase OS=Homo sapiens GN=TPMT | 1.243 | Up | 0.032062 | TPMT |
| O43242 | 26S proteasome non-ATPase regulatory subunit 3 OS=Homo sapiens GN=PSMD3 | 1.242 | Up | 0.002915 | PSMD3 |
| P61158 | Actin-related protein 3 OS=Homo sapiens GN=ACTR3 | 1.241 | Up | 3.74E-06 | ACTR3 |
| O00571 | ATP-dependent RNA helicase DDX3X OS=Homo sapiens GN=DDX3X | 1.238 | Up | 0.010541 | DDX3X |
| Q8N806 | Putative E3 ubiquitin-protein ligase UBR7 OS=Homo sapiens GN=UBR7 | 1.237 | Up | 0.032377 | UBR7 |
| Q13347 | Eukaryotic translation initiation factor 3 subunit I OS=Homo sapiens GN=EIF3I | 1.237 | Up | 0.040044 | EIF3I |
| Q16555 | Dihydropyrimidinase-related protein 2 OS=Homo sapiens GN=DPYSL2 | 1.236 | Up | 0.005481 | DPYSL2 |
| P30626 | Sorcin OS=Homo sapiens GN=SRI | 1.236 | Up | 0.004703 | SRI |
| Q9BT78 | COP9 signalosome complex subunit 4 OS=Homo sapiens GN=COPS4 | 1.236 | Up | 0.006637 | COPS4 |
| P35606 | Coatomer subunit beta' OS=Homo sapiens GN=COPB2 | 1.236 | Up | 5.76E-05 | COPB2 |
| P23246 | Splicing factor, proline- and glutamine-rich OS=Homo sapiens GN=SFPQ | 1.236 | Up | 0.003898 | SFPQ |
| O00231 | 26S proteasome non-ATPase regulatory subunit 11 OS=Homo sapiens GN=PSMD11 | 1.233 | Up | 0.007645 | PSMD11 |
| P07900 | Heat shock protein HSP 90-alpha OS=Homo sapiens GN=HSP90AA1 | 1.233 | Up | 8.08E-09 | HSP90AA1 |
| Q8IYE0 | Coiled-coil domain-containing protein 146 OS=Homo sapiens GN=CCDC146 | 1.232 | Up | 0.00776 | CCDC146 |
| P51665 | 26S proteasome non-ATPase regulatory subunit 7 OS=Homo sapiens GN=PSMD7 | 1.232 | Up | 0.023784 | PSMD7 |
| Q9UGI8 | Testin OS=Homo sapiens GN=TES | 1.232 | Up | 0.048724 | TES |
| Q9Y230 | RuvB-like 2 OS=Homo sapiens GN=RUVBL2 | 1.231 | Up | 0.002701 | RUVBL2 |
| O95757 | Heat shock 70 kDa protein 4L OS=Homo sapiens GN=HSPA4L | 1.23 | Up | 0.003161 | HSPA4L |
| Q9Y2Z0 | Protein SGT1 homolog OS=Homo sapiens GN=SUGT1 | 1.23 | Up | 0.003281 | SUGT1 |
| Q9ULV4 | Coronin-1C OS=Homo sapiens GN=CORO1C | 1.229 | Up | 0.000684 | CORO1C |
| Q86VP6 | Cullin-associated NEDD8-dissociated protein 1 OS=Homo sapiens GN=CAND1 | 1.228 | Up | 1.88E-11 | CAND1 |
| P12268 | Inosine-5'-monophosphate dehydrogenase 2 OS=Homo sapiens GN=IMPDH2 | 1.228 | Up | 0.01038 | IMPDH2 |
| P55036 | 26S proteasome non-ATPase regulatory subunit 4 OS=Homo sapiens GN=PSMD4 | 1.227 | Up | 0.000316 | PSMD4 |
| Q9Y2A7 | Nck-associated protein 1 OS=Homo sapiens GN=NCKAP1 | 1.225 | Up | 0.011964 | NCKAP1 |
| Q14203 | Dynactin subunit 1 OS=Homo sapiens GN=DCTN1 | 1.224 | Up | 5.81E-05 | DCTN1 |
| P26641 | Elongation factor 1-gamma OS=Homo sapiens GN=EEF1G | 1.223 | Up | 0.014344 | EEF1G |
| P46777 | 60S ribosomal protein L5 OS=Homo sapiens GN=RPL5 | 1.222 | Up | 0.040718 | RPL5 |
| Q00688 | Peptidyl-prolyl cis-trans isomerase FKBP3 OS=Homo sapiens GN=FKBP3 | 1.221 | Up | 0.000883 | FKBP3 |
| O60506 | Heterogeneous nuclear ribonucleoprotein Q OS=Homo sapiens GN=SYNCRIP | 1.221 | Up | 7.9E-05 | SYNCRIP |
| Q9UK22 | F-box only protein 2 OS=Homo sapiens GN=FBXO2 | 1.218 | Up | 0.002417 | FBXO2 |
| O43143 | Pre-mRNA-splicing factor ATP-dependent RNA helicase DHX15 OS=Homo sapiens GN=DHX15 | 1.217 | Up | 0.013704 | DHX15 |
| Q9BY44 | Eukaryotic translation initiation factor 2A OS=Homo sapiens GN=EIF2A | 1.217 | Up | 0.002803 | EIF2A |
| Q9BQ67 | Glutamate-rich WD repeat-containing protein 1 OS=Homo sapiens GN=GRWD1 | 1.215 | Up | 0.048457 | GRWD1 |
| P53621 | Coatomer subunit alpha OS=Homo sapiens GN=COPA | 1.212 | Up | 0.002844 | COPA |
| P49591 | Serine--tRNA ligase, cytoplasmic OS=Homo sapiens GN=SARS | 1.211 | Up | 0.044102 | SARS |
| P07814 | Bifunctional glutamate/proline--tRNA ligase OS=Homo sapiens GN=EPRS | 1.21 | Up | 2.36E-05 | EPRS |
| Q9UHD8 | Septin-9 OS=Homo sapiens GN=SEPT9 | 1.208 | Up | 0.045885 | SEPT9 |
| P33176 | Kinesin-1 heavy chain OS=Homo sapiens GN=KIF5B | 1.206 | Up | 0.022602 | KIF5B |
| P08133 | Annexin A6 OS=Homo sapiens GN=ANXA6 | 1.205 | Up | 0.003337 | ANXA6 |
| P68104 | Elongation factor 1-alpha 1 OS=Homo sapiens GN=EEF1A1 | 1.205 | Up | 0.034224 | EEF1A1 |
| P43490 | Nicotinamide phosphoribosyltransferase OS=Homo sapiens GN=NAMPT | 1.205 | Up | 0.013702 | NAMPT |
| P61160 | Actin-related protein 2 OS=Homo sapiens GN=ACTR2 | 1.205 | Up | 0.000384 | ACTR2 |
| P48444 | Coatomer subunit delta OS=Homo sapiens GN=ARCN1 | 1.204 | Up | 0.015078 | ARCN1 |
| P53004 | Biliverdin reductase A OS=Homo sapiens GN=BLVRA | 1.202 | Up | 0.005341 | BLVRA |
| Q13263 | Transcription intermediary factor 1-beta OS=Homo sapiens GN=TRIM28 | 1.202 | Up | 0.0008 | TRIM28 |
| Q9BZZ5 | Apoptosis inhibitor 5 OS=Homo sapiens GN=API5 | 1.202 | Up | 0.012117 | API5 |
| O00410 | Importin-5 OS=Homo sapiens GN=IPO5 | 1.201 | Up | 0.000464 | IPO5 |
| Q02388 | Collagen alpha-1(VII) chain OS=Homo sapiens GN=COL7A1 | 0.832 | Down | 0.034576 | COL7A1 |
| P00491 | Purine nucleoside phosphorylase OS=Homo sapiens GN=PNP | 0.832 | Down | 0.002341 | PNP |
| Q92626 | Peroxidasin homolog OS=Homo sapiens GN=PXDN | 0.83 | Down | 0.006241 | PXDN |
| Q9NYJ1 | Cytochrome c oxidase assembly factor 4 homolog, mitochondrial OS=Homo sapiens GN=COA4 | 0.829 | Down | 0.003619 | COA4 |
| P40926 | Malate dehydrogenase, mitochondrial OS=Homo sapiens GN=MDH2 | 0.828 | Down | 0.000204 | MDH2 |
| Q16531 | DNA damage-binding protein 1 OS=Homo sapiens GN=DDB1 | 0.826 | Down | 1.03E-06 | DDB1 |
| P09429 | High mobility group protein B1 OS=Homo sapiens GN=HMGB1 | 0.824 | Down | 0.00014 | HMGB1 |
| P46013 | Proliferation marker protein Ki-67 OS=Homo sapiens GN=MKI67 | 0.823 | Down | 0.020456 | MKI67 |
| P07954 | Fumarate hydratase, mitochondrial OS=Homo sapiens GN=FH | 0.822 | Down | 9.95E-05 | FH |
| P13693 | Translationally-controlled tumor protein OS=Homo sapiens GN=TPT1 | 0.819 | Down | 3.59E-05 | TPT1 |
| P16949 | Stathmin OS=Homo sapiens GN=STMN1 | 0.818 | Down | 0.042063 | STMN1 |
| Q99715 | Collagen alpha-1(XII) chain OS=Homo sapiens GN=COL12A1 | 0.817 | Down | 0.000265 | COL12A1 |
| Q9UHB6 | LIM domain and actin-binding protein 1 OS=Homo sapiens GN=LIMA1 | 0.817 | Down | 0.022222 | LIMA1 |
| P08670 | Vimentin OS=Homo sapiens GN=VIM | 0.816 | Down | 0.024099 | VIM |
| Q8NCW5 | NAD(P)H-hydrate epimerase OS=Homo sapiens GN=NAXE | 0.816 | Down | 0.00402 | NAXE |
| Q9UHY7 | Enolase-phosphatase E1 OS=Homo sapiens GN=ENOPH1 | 0.815 | Down | 0.024423 | ENOPH1 |
| Q9Y383 | Putative RNA-binding protein Luc7-like 2 OS=Homo sapiens GN=LUC7L2 | 0.814 | Down | 0.0313 | LUC7L2 |
| P34096 | Ribonuclease 4 OS=Homo sapiens GN=RNASE4 | 0.812 | Down | 0.041096 | RNASE4 |
| P50402 | Emerin OS=Homo sapiens GN=EMD | 0.812 | Down | 0.033463 | EMD |
| O75390 | Citrate synthase, mitochondrial OS=Homo sapiens GN=CS | 0.81 | Down | 1.86E-05 | CS |
| P42704 | Leucine-rich PPR motif-containing protein, mitochondrial OS=Homo sapiens GN=LRPPRC | 0.808 | Down | 6.71E-07 | LRPPRC |
| P30040 | Endoplasmic reticulum resident protein 29 OS=Homo sapiens GN=ERP29 | 0.808 | Down | 4.21E-05 | ERP29 |
| O00391 | Sulfhydryl oxidase 1 OS=Homo sapiens GN=QSOX1 | 0.805 | Down | 9.76E-05 | QSOX1 |
| P10586 | Receptor-type tyrosine-protein phosphatase F OS=Homo sapiens GN=PTPRF | 0.804 | Down | 0.00044 | PTPRF |
| P67809 | Nuclease-sensitive element-binding protein 1 OS=Homo sapiens GN=YBX1 | 0.803 | Down | 0.024983 | YBX1 |
| Q14517 | Protocadherin Fat 1 OS=Homo sapiens GN=FAT1 | 0.798 | Down | 0.001763 | FAT1 |
| Q99436 | Proteasome subunit beta type-7 OS=Homo sapiens GN=PSMB7 | 0.798 | Down | 1.65E-05 | PSMB7 |
| P30084 | Enoyl-CoA hydratase, mitochondrial OS=Homo sapiens GN=ECHS1 | 0.796 | Down | 0.00024 | ECHS1 |
| Q13332 | Receptor-type tyrosine-protein phosphatase S OS=Homo sapiens GN=PTPRS | 0.794 | Down | 0.008502 | PTPRS |
| Q9UHI8 | A disintegrin and metalloproteinase with thrombospondin motifs 1 OS=Homo sapiens GN=ADAMTS1 | 0.791 | Down | 0.01222 | ADAMTS1 |
| Q9UBX1 | Cathepsin F OS=Homo sapiens GN=CTSF | 0.789 | Down | 0.018543 | CTSF |
| Q9Y5B9 | FACT complex subunit SPT16 OS=Homo sapiens GN=SUPT16H | 0.787 | Down | 0.005639 | SUPT16H |
| Q00839 | Heterogeneous nuclear ribonucleoprotein U OS=Homo sapiens GN=HNRNPU | 0.786 | Down | 0.007896 | HNRNPU |
| Q9Y4L1 | Hypoxia up-regulated protein 1 OS=Homo sapiens GN=HYOU1 | 0.782 | Down | 0.020679 | HYOU1 |
| P62158 | Calmodulin OS=Homo sapiens GN=CALM1 | 0.782 | Down | 1.66E-06 | CALM1 |
| Q92896 | Golgi apparatus protein 1 OS=Homo sapiens GN=GLG1 | 0.776 | Down | 0.032363 | GLG1 |
| P28072 | Proteasome subunit beta type-6 OS=Homo sapiens GN=PSMB6 | 0.776 | Down | 0.017916 | PSMB6 |
| Q969P0 | Immunoglobulin superfamily member 8 OS=Homo sapiens GN=IGSF8 | 0.773 | Down | 0.044941 | IGSF8 |
| Q07021 | Complement component 1 Q subcomponent-binding protein, mitochondrial OS=Homo sapiens GN=C1QBP | 0.769 | Down | 1.8E-07 | C1QBP |
| Q12805 | EGF-containing fibulin-like extracellular matrix protein 1 OS=Homo sapiens GN=EFEMP1 | 0.767 | Down | 0.002283 | EFEMP1 |
| P00367 | Glutamate dehydrogenase 1, mitochondrial OS=Homo sapiens GN=GLUD1 | 0.767 | Down | 0.004123 | GLUD1 |
| P23142 | Fibulin-1 OS=Homo sapiens GN=FBLN1 | 0.765 | Down | 0.000263 | FBLN1 |
| O94760 | N(G),N(G)-dimethylarginine dimethylaminohydrolase 1 OS=Homo sapiens GN=DDAH1 | 0.764 | Down | 0.024083 | DDAH1 |
| P23284 | Peptidyl-prolyl cis-trans isomerase B OS=Homo sapiens GN=PPIB | 0.761 | Down | 2.63E-06 | PPIB |
| Q9NS15 | Latent-transforming growth factor beta-binding protein 3 OS=Homo sapiens GN=LTBP3 | 0.76 | Down | 0.001117 | LTBP3 |
| P01891 | HLA class I histocompatibility antigen, A-68 alpha chain OS=Homo sapiens GN=HLA-A | 0.76 | Down | 0.008165 | HLA-A |
| P16401 | Histone H1.5 OS=Homo sapiens GN=HIST1H1B | 0.759 | Down | 0.005663 | HIST1H1B |
| P20700 | Lamin-B1 OS=Homo sapiens GN=LMNB1 | 0.759 | Down | 3.6E-05 | LMNB1 |
| Q12888 | Tumor suppressor p53-binding protein 1 OS=Homo sapiens GN=TP53BP1 | 0.758 | Down | 0.020457 | TP53BP1 |
| Q9UBI6 | Guanine nucleotide-binding protein G(I)/G(S)/G(O) subunit gamma-12 OS=Homo sapiens GN=GNG12 | 0.756 | Down | 0.000261 | GNG12 |
| Q08345 | Epithelial discoidin domain-containing receptor 1 OS=Homo sapiens GN=DDR1 | 0.755 | Down | 0.000205 | DDR1 |
| P06280 | Alpha-galactosidase A OS=Homo sapiens GN=GLA | 0.753 | Down | 1.02E-06 | GLA |
| Q01105 | Protein SET OS=Homo sapiens GN=SET | 0.753 | Down | 0.003318 | SET |
| Q8NBJ7 | Sulfatase-modifying factor 2 OS=Homo sapiens GN=SUMF2 | 0.752 | Down | 0.010038 | SUMF2 |
| P00742 | Coagulation factor X OS=Homo sapiens GN=F10 | 0.752 | Down | 0.000159 | F10 |
| Q8NBP7 | Proprotein convertase subtilisin/kexin type 9 OS=Homo sapiens GN=PCSK9 | 0.751 | Down | 0.000165 | PCSK9 |
| Q9BY67 | Cell adhesion molecule 1 OS=Homo sapiens GN=CADM1 | 0.749 | Down | 0.006003 | CADM1 |
| P17936 | Insulin-like growth factor-binding protein 3 OS=Homo sapiens GN=IGFBP3 | 0.747 | Down | 2.32E-05 | IGFBP3 |
| P38571 | Lysosomal acid lipase/cholesteryl ester hydrolase OS=Homo sapiens GN=LIPA | 0.746 | Down | 0.008798 | LIPA |
| P42126 | Enoyl-CoA delta isomerase 1, mitochondrial OS=Homo sapiens GN=ECI1 | 0.745 | Down | 0.045579 | ECI1 |
| Q969H8 | Myeloid-derived growth factor OS=Homo sapiens GN=MYDGF | 0.744 | Down | 0.00276 | MYDGF |
| O60568 | Procollagen-lysine,2-oxoglutarate 5-dioxygenase 3 OS=Homo sapiens GN=PLOD3 | 0.743 | Down | 9.45E-07 | PLOD3 |
| P50213 | Isocitrate dehydrogenase [NAD] subunit alpha, mitochondrial OS=Homo sapiens GN=IDH3A | 0.742 | Down | 0.04122 | IDH3A |
| Q9H0H5 | Rac GTPase-activating protein 1 OS=Homo sapiens GN=RACGAP1 | 0.741 | Down | 0.009463 | RACGAP1 |
| Q9UNW1 | Multiple inositol polyphosphate phosphatase 1 OS=Homo sapiens GN=MINPP1 | 0.737 | Down | 0.002141 | MINPP1 |
| P06748 | Nucleophosmin OS=Homo sapiens GN=NPM1 | 0.735 | Down | 1.6E-05 | NPM1 |
| P24593 | Insulin-like growth factor-binding protein 5 OS=Homo sapiens GN=IGFBP5 | 0.734 | Down | 0.004963 | IGFBP5 |
| P55268 | Laminin subunit beta-2 OS=Homo sapiens GN=LAMB2 | 0.733 | Down | 8.7E-07 | LAMB2 |
| O95302 | Peptidyl-prolyl cis-trans isomerase FKBP9 OS=Homo sapiens GN=FKBP9 | 0.732 | Down | 0.001957 | FKBP9 |
| Q9UKV3 | Apoptotic chromatin condensation inducer in the nucleus OS=Homo sapiens GN=ACIN1 | 0.731 | Down | 0.004522 | ACIN1 |
| Q9BWS9 | Chitinase domain-containing protein 1 OS=Homo sapiens GN=CHID1 | 0.731 | Down | 4.29E-05 | CHID1 |
| Q9BRK5 | 45 kDa calcium-binding protein OS=Homo sapiens GN=SDF4 | 0.731 | Down | 0.000862 | SDF4 |
| P39687 | Acidic leucine-rich nuclear phosphoprotein 32 family member A OS=Homo sapiens GN=ANP32A | 0.731 | Down | 0.0005 | ANP32A |
| P23434 | Glycine cleavage system H protein, mitochondrial OS=Homo sapiens GN=GCSH | 0.73 | Down | 0.007984 | GCSH |
| P19338 | Nucleolin OS=Homo sapiens GN=NCL | 0.728 | Down | 2.78E-12 | NCL |
| Q9Y2B0 | Protein canopy homolog 2 OS=Homo sapiens GN=CNPY2 | 0.727 | Down | 0.004363 | CNPY2 |
| P10809 | 60 kDa heat shock protein, mitochondrial OS=Homo sapiens GN=HSPD1 | 0.726 | Down | 4.51E-07 | HSPD1 |
| P61604 | 10 kDa heat shock protein, mitochondrial OS=Homo sapiens GN=HSPE1 | 0.725 | Down | 1.26E-08 | HSPE1 |
| P55145 | Mesencephalic astrocyte-derived neurotrophic factor OS=Homo sapiens GN=MANF | 0.721 | Down | 6.27E-09 | MANF |
| Q15155 | Nodal modulator 1 OS=Homo sapiens GN=NOMO1 | 0.721 | Down | 0.016565 | NOMO1 |
| P09622 | Dihydrolipoyl dehydrogenase, mitochondrial OS=Homo sapiens GN=DLD | 0.721 | Down | 0.000804 | DLD |
| P06576 | ATP synthase subunit beta, mitochondrial OS=Homo sapiens GN=ATP5B | 0.718 | Down | 4.28E-06 | ATP5B |
| Q15582 | Transforming growth factor-beta-induced protein ig-h3 OS=Homo sapiens GN=TGFBI | 0.718 | Down | 3.14E-08 | TGFBI |
| P00505 | Aspartate aminotransferase, mitochondrial OS=Homo sapiens GN=GOT2 | 0.717 | Down | 3.24E-11 | GOT2 |
| Q92688 | Acidic leucine-rich nuclear phosphoprotein 32 family member B OS=Homo sapiens GN=ANP32B | 0.716 | Down | 4.77E-06 | ANP32B |
| P00751 | Complement factor B OS=Homo sapiens GN=CFB | 0.716 | Down | 0.005401 | CFB |
| P11362 | Fibroblast growth factor receptor 1 OS=Homo sapiens GN=FGFR1 | 0.715 | Down | 0.037398 | FGFR1 |
| Q9Y2W1 | Thyroid hormone receptor-associated protein 3 OS=Homo sapiens GN=THRAP3 | 0.713 | Down | 0.005965 | THRAP3 |
| P46087 | Probable 28S rRNA (cytosine(4447)-C(5))-methyltransferase OS=Homo sapiens GN=NOP2 | 0.712 | Down | 0.030179 | NOP2 |
| Q13275 | Semaphorin-3F OS=Homo sapiens GN=SEMA3F | 0.712 | Down | 0.019881 | SEMA3F |
| P06454 | Prothymosin alpha OS=Homo sapiens GN=PTMA | 0.711 | Down | 0.042261 | PTMA |
| Q9GZL7 | Ribosome biogenesis protein WDR12 OS=Homo sapiens GN=WDR12 | 0.71 | Down | 0.025302 | WDR12 |
| Q15007 | Pre-mRNA-splicing regulator WTAP OS=Homo sapiens GN=WTAP | 0.708 | Down | 0.033078 | WTAP |
| Q13214 | Semaphorin-3B OS=Homo sapiens GN=SEMA3B | 0.708 | Down | 0.000882 | SEMA3B |
| P24821 | Tenascin OS=Homo sapiens GN=TNC | 0.703 | Down | 0.002222 | TNC |
| P50895 | Basal cell adhesion molecule OS=Homo sapiens GN=BCAM | 0.702 | Down | 2.56E-06 | BCAM |
| P07339 | Cathepsin D OS=Homo sapiens GN=CTSD | 0.702 | Down | 1.17E-08 | CTSD |
| Q16629 | Serine/arginine-rich splicing factor 7 OS=Homo sapiens GN=SRSF7 | 0.702 | Down | 0.003419 | SRSF7 |
| P19021 | Peptidyl-glycine alpha-amidating monooxygenase OS=Homo sapiens GN=PAM | 0.702 | Down | 0.020384 | PAM |
| Q15262 | Receptor-type tyrosine-protein phosphatase kappa OS=Homo sapiens GN=PTPRK | 0.702 | Down | 0.013339 | PTPRK |
| Q9BUD6 | Spondin-2 OS=Homo sapiens GN=SPON2 | 0.7 | Down | 0.004545 | SPON2 |
| P35555 | Fibrillin-1 OS=Homo sapiens GN=FBN1 | 0.7 | Down | 3E-09 | FBN1 |
| P10909 | Clusterin OS=Homo sapiens GN=CLU | 0.698 | Down | 9.72E-07 | CLU |
| Q6UVK1 | Chondroitin sulfate proteoglycan 4 OS=Homo sapiens GN=CSPG4 | 0.697 | Down | 3.99E-08 | CSPG4 |
| P48509 | CD151 antigen OS=Homo sapiens GN=CD151 | 0.696 | Down | 0.028236 | CD151 |
| P08253 | 72 kDa type IV collagenase OS=Homo sapiens GN=MMP2 | 0.694 | Down | 4.02E-05 | MMP2 |
| Q9GZP8 | Immortalization up-regulated protein OS=Homo sapiens GN=IMUP | 0.694 | Down | 0.032779 | IMUP |
| P98160 | Basement membrane-specific heparan sulfate proteoglycan core protein OS=Homo sapiens GN=HSPG2 | 0.693 | Down | 1.62E-12 | HSPG2 |
| Q9Y6N7 | Roundabout homolog 1 OS=Homo sapiens GN=ROBO1 | 0.693 | Down | 0.003319 | ROBO1 |
| P55809 | Succinyl-CoA:3-ketoacid coenzyme A transferase 1, mitochondrial OS=Homo sapiens GN=OXCT1 | 0.691 | Down | 6.47E-09 | OXCT1 |
| Q8NBS9 | Thioredoxin domain-containing protein 5 OS=Homo sapiens GN=TXNDC5 | 0.691 | Down | 8.06E-08 | TXNDC5 |
| Q4LDE5 | Sushi, von Willebrand factor type A, EGF and pentraxin domain-containing protein 1 OS=Homo sapiens GN=SVEP1 | 0.69 | Down | 0.000221 | SVEP1 |
| O43278 | Kunitz-type protease inhibitor 1 OS=Homo sapiens GN=SPINT1 | 0.689 | Down | 0.004545 | SPINT1 |
| P00736 | Complement C1r subcomponent OS=Homo sapiens GN=C1R | 0.686 | Down | 0.000382 | C1R |
| P29120 | Neuroendocrine convertase 1 OS=Homo sapiens GN=PCSK1 | 0.685 | Down | 1.87E-05 | PCSK1 |
| P01130 | Low-density lipoprotein receptor OS=Homo sapiens GN=LDLR | 0.684 | Down | 0.001443 | LDLR |
| Q13428 | Treacle protein OS=Homo sapiens GN=TCOF1 | 0.683 | Down | 0.000739 | TCOF1 |
| Q8NFH4 | Nucleoporin Nup37 OS=Homo sapiens GN=NUP37 | 0.683 | Down | 0.034977 | NUP37 |
| Q14563 | Semaphorin-3A OS=Homo sapiens GN=SEMA3A | 0.681 | Down | 0.000221 | SEMA3A |
| Q86SR1 | Polypeptide N-acetylgalactosaminyltransferase 10 OS=Homo sapiens GN=GALNT10 | 0.68 | Down | 0.023038 | GALNT10 |
| Q9NPR2 | Semaphorin-4B OS=Homo sapiens GN=SEMA4B | 0.679 | Down | 5.81E-05 | SEMA4B |
| P04062 | Glucosylceramidase OS=Homo sapiens GN=GBA | 0.677 | Down | 0.000578 | GBA |
| Q03252 | Lamin-B2 OS=Homo sapiens GN=LMNB2 | 0.676 | Down | 0.000464 | LMNB2 |
| Q15113 | Procollagen C-endopeptidase enhancer 1 OS=Homo sapiens GN=PCOLCE | 0.675 | Down | 2.81E-12 | PCOLCE |
| Q07955 | Serine/arginine-rich splicing factor 1 OS=Homo sapiens GN=SRSF1 | 0.673 | Down | 7.42E-09 | SRSF1 |
| O76076 | WNT1-inducible-signaling pathway protein 2 OS=Homo sapiens GN=WISP2 | 0.671 | Down | 0.002143 | WISP2 |
| P54760 | Ephrin type-B receptor 4 OS=Homo sapiens GN=EPHB4 | 0.67 | Down | 0.010298 | EPHB4 |
| P28799 | Granulins OS=Homo sapiens GN=GRN | 0.669 | Down | 0.000179 | GRN |
| Q10472 | Polypeptide N-acetylgalactosaminyltransferase 1 OS=Homo sapiens GN=GALNT1 | 0.669 | Down | 9.68E-05 | GALNT1 |
| P07858 | Cathepsin B OS=Homo sapiens GN=CTSB | 0.668 | Down | 0.010383 | CTSB |
| Q07954 | Prolow-density lipoprotein receptor-related protein 1 OS=Homo sapiens GN=LRP1 | 0.668 | Down | 1.51E-09 | LRP1 |
| P05187 | Alkaline phosphatase, placental type OS=Homo sapiens GN=ALPP | 0.668 | Down | 0.003705 | ALPP |
| Q9UM22 | Mammalian ependymin-related protein 1 OS=Homo sapiens GN=EPDR1 | 0.667 | Down | 0.023556 | EPDR1 |
| Q99574 | Neuroserpin OS=Homo sapiens GN=SERPINI1 | 0.666 | Down | 3.69E-10 | SERPINI1 |
| P53999 | Activated RNA polymerase II transcriptional coactivator p15 OS=Homo sapiens GN=SUB1 | 0.665 | Down | 3.83E-08 | SUB1 |
| P62906 | 60S ribosomal protein L10a OS=Homo sapiens GN=RPL10A | 0.664 | Down | 5.69E-05 | RPL10A |
| Q15293 | Reticulocalbin-1 OS=Homo sapiens GN=RCN1 | 0.663 | Down | 3.97E-14 | RCN1 |
| P14543 | Nidogen-1 OS=Homo sapiens GN=NID1 | 0.662 | Down | 0.000224 | NID1 |
| O95881 | Thioredoxin domain-containing protein 12 OS=Homo sapiens GN=TXNDC12 | 0.662 | Down | 0.001221 | TXNDC12 |
| P13667 | Protein disulfide-isomerase A4 OS=Homo sapiens GN=PDIA4 | 0.661 | Down | 1E-32 | PDIA4 |
| O95084 | Serine protease 23 OS=Homo sapiens GN=PRSS23 | 0.659 | Down | 0.003041 | PRSS23 |
| Q15904 | V-type proton ATPase subunit S1 OS=Homo sapiens GN=ATP6AP1 | 0.656 | Down | 0.011901 | ATP6AP1 |
| Q6WRI0 | Immunoglobulin superfamily member 10 OS=Homo sapiens GN=IGSF10 | 0.654 | Down | 0.007356 | IGSF10 |
| P13284 | Gamma-interferon-inducible lysosomal thiol reductase OS=Homo sapiens GN=IFI30 | 0.653 | Down | 3.09E-06 | IFI30 |
| P17900 | Ganglioside GM2 activator OS=Homo sapiens GN=GM2A | 0.652 | Down | 1.3E-07 | GM2A |
| P10646 | Tissue factor pathway inhibitor OS=Homo sapiens GN=TFPI | 0.648 | Down | 0.008997 | TFPI |
| Q8N2S1 | Latent-transforming growth factor beta-binding protein 4 OS=Homo sapiens GN=LTBP4 | 0.648 | Down | 8.18E-09 | LTBP4 |
| P30101 | Protein disulfide-isomerase A3 OS=Homo sapiens GN=PDIA3 | 0.648 | Down | 1.62E-12 | PDIA3 |
| Q14112 | Nidogen-2 OS=Homo sapiens GN=NID2 | 0.647 | Down | 0.014464 | NID2 |
| P42785 | Lysosomal Pro-X carboxypeptidase OS=Homo sapiens GN=PRCP | 0.645 | Down | 0.001762 | PRCP |
| P39060 | Collagen alpha-1(XVIII) chain OS=Homo sapiens GN=COL18A1 | 0.643 | Down | 0.04398 | COL18A1 |
| Q13361 | Microfibrillar-associated protein 5 OS=Homo sapiens GN=MFAP5 | 0.643 | Down | 5.94E-08 | MFAP5 |
| P15289 | Arylsulfatase A OS=Homo sapiens GN=ARSA | 0.642 | Down | 0.038457 | ARSA |
| P07237 | Protein disulfide-isomerase OS=Homo sapiens GN=P4HB | 0.642 | Down | 1E-32 | P4HB |
| Q02809 | Procollagen-lysine,2-oxoglutarate 5-dioxygenase 1 OS=Homo sapiens GN=PLOD1 | 0.642 | Down | 1.64E-12 | PLOD1 |
| P20851 | C4b-binding protein beta chain OS=Homo sapiens GN=C4BPB | 0.64 | Down | 0.018556 | C4BPB |
| P02786 | Transferrin receptor protein 1 OS=Homo sapiens GN=TFRC | 0.64 | Down | 6.43E-13 | TFRC |
| P05556 | Integrin beta-1 OS=Homo sapiens GN=ITGB1 | 0.636 | Down | 0.00036 | ITGB1 |
| Q16610 | Extracellular matrix protein 1 OS=Homo sapiens GN=ECM1 | 0.636 | Down | 3.07E-08 | ECM1 |
| O76061 | Stanniocalcin-2 OS=Homo sapiens GN=STC2 | 0.635 | Down | 0.003796 | STC2 |
| P10619 | Lysosomal protective protein OS=Homo sapiens GN=CTSA | 0.635 | Down | 2.37E-05 | CTSA |
| P98095 | Fibulin-2 OS=Homo sapiens GN=FBLN2 | 0.634 | Down | 1.85E-05 | FBLN2 |
| P01024 | Complement C3 OS=Homo sapiens GN=C3 | 0.634 | Down | 1E-32 | C3 |
| P11717 | Cation-independent mannose-6-phosphate receptor OS=Homo sapiens GN=IGF2R | 0.632 | Down | 4.69E-10 | IGF2R |
| Q06481 | Amyloid-like protein 2 OS=Homo sapiens GN=APLP2 | 0.631 | Down | 6.53E-08 | APLP2 |
| Q71DI3 | Histone H3.2 OS=Homo sapiens GN=HIST2H3A | 0.63 | Down | 0.001364 | HIST2H3A |
| P25391 | Laminin subunit alpha-1 OS=Homo sapiens GN=LAMA1 | 0.627 | Down | 4.11E-05 | LAMA1 |
| Q9UBQ6 | Exostosin-like 2 OS=Homo sapiens GN=EXTL2 | 0.627 | Down | 0.0001 | EXTL2 |
| Q99985 | Semaphorin-3C OS=Homo sapiens GN=SEMA3C | 0.626 | Down | 0.003958 | SEMA3C |
| P01034 | Cystatin-C OS=Homo sapiens GN=CST3 | 0.624 | Down | 2.56E-13 | CST3 |
| P35556 | Fibrillin-2 OS=Homo sapiens GN=FBN2 | 0.623 | Down | 1.62E-12 | FBN2 |
| O43505 | Beta-1,4-glucuronyltransferase 1 OS=Homo sapiens GN=B4GAT1 | 0.623 | Down | 0.019581 | B4GAT1 |
| Q92520 | Protein FAM3C OS=Homo sapiens GN=FAM3C | 0.622 | Down | 5.46E-09 | FAM3C |
| P11021 | 78 kDa glucose-regulated protein OS=Homo sapiens GN=HSPA5 | 0.621 | Down | 1E-32 | HSPA5 |
| P21810 | Biglycan OS=Homo sapiens GN=BGN | 0.621 | Down | 0.001184 | BGN |
| P08572 | Collagen alpha-2(IV) chain OS=Homo sapiens GN=COL4A2 | 0.621 | Down | 0.0009 | COL4A2 |
| Q9UHL4 | Dipeptidyl peptidase 2 OS=Homo sapiens GN=DPP7 | 0.619 | Down | 0.005683 | DPP7 |
| Q16706 | Alpha-mannosidase 2 OS=Homo sapiens GN=MAN2A1 | 0.617 | Down | 0.000419 | MAN2A1 |
| Q14978 | Nucleolar and coiled-body phosphoprotein 1 OS=Homo sapiens GN=NOLC1 | 0.617 | Down | 0.026562 | NOLC1 |
| P11047 | Laminin subunit gamma-1 OS=Homo sapiens GN=LAMC1 | 0.615 | Down | 1E-32 | LAMC1 |
| P04179 | Superoxide dismutase [Mn], mitochondrial OS=Homo sapiens GN=SOD2 | 0.614 | Down | 1.79E-06 | SOD2 |
| O00468 | Agrin OS=Homo sapiens GN=AGRN | 0.613 | Down | 1.62E-12 | AGRN |
| P03950 | Angiogenin OS=Homo sapiens GN=ANG | 0.613 | Down | 0.015958 | ANG |
| Q13247 | Serine/arginine-rich splicing factor 6 OS=Homo sapiens GN=SRSF6 | 0.612 | Down | 0.029957 | SRSF6 |
| Q14126 | Desmoglein-2 OS=Homo sapiens GN=DSG2 | 0.611 | Down | 9.87E-09 | DSG2 |
| P10124 | Serglycin OS=Homo sapiens GN=SRGN | 0.61 | Down | 0.007875 | SRGN |
| O15230 | Laminin subunit alpha-5 OS=Homo sapiens GN=LAMA5 | 0.609 | Down | 1.62E-12 | LAMA5 |
| P55001 | Microfibrillar-associated protein 2 OS=Homo sapiens GN=MFAP2 | 0.609 | Down | 0.008395 | MFAP2 |
| P15529 | Membrane cofactor protein OS=Homo sapiens GN=CD46 | 0.609 | Down | 0.002578 | CD46 |
| Q86X29 | Lipolysis-stimulated lipoprotein receptor OS=Homo sapiens GN=LSR | 0.608 | Down | 0.02624 | LSR |
| Q9H3T3 | Semaphorin-6B OS=Homo sapiens GN=SEMA6B | 0.608 | Down | 0.002662 | SEMA6B |
| P51884 | Lumican OS=Homo sapiens GN=LUM | 0.604 | Down | 4.36E-05 | LUM |
| P07602 | Prosaposin OS=Homo sapiens GN=PSAP | 0.604 | Down | 1E-32 | PSAP |
| P08581 | Hepatocyte growth factor receptor OS=Homo sapiens GN=MET | 0.603 | Down | 3.27E-08 | MET |
| P35052 | Glypican-1 OS=Homo sapiens GN=GPC1 | 0.601 | Down | 8.51E-10 | GPC1 |
| P16035 | Metalloproteinase inhibitor 2 OS=Homo sapiens GN=TIMP2 | 0.6 | Down | 9.62E-13 | TIMP2 |
| Q9NZ08 | Endoplasmic reticulum aminopeptidase 1 OS=Homo sapiens GN=ERAP1 | 0.6 | Down | 0.002383 | ERAP1 |
| P14314 | Glucosidase 2 subunit beta OS=Homo sapiens GN=PRKCSH | 0.597 | Down | 1.36E-10 | PRKCSH |
| O94985 | Calsyntenin-1 OS=Homo sapiens GN=CLSTN1 | 0.595 | Down | 1E-32 | CLSTN1 |
| Q13162 | Peroxiredoxin-4 OS=Homo sapiens GN=PRDX4 | 0.593 | Down | 0.000161 | PRDX4 |
| P51572 | B-cell receptor-associated protein 31 OS=Homo sapiens GN=BCAP31 | 0.591 | Down | 0.000743 | BCAP31 |
| P55290 | Cadherin-13 OS=Homo sapiens GN=CDH13 | 0.59 | Down | 0.001762 | CDH13 |
| Q15818 | Neuronal pentraxin-1 OS=Homo sapiens GN=NPTX1 | 0.589 | Down | 1.5E-05 | NPTX1 |
| Q14766 | Latent-transforming growth factor beta-binding protein 1 OS=Homo sapiens GN=LTBP1 | 0.588 | Down | 2.37E-10 | LTBP1 |
| P00533 | Epidermal growth factor receptor OS=Homo sapiens GN=EGFR | 0.586 | Down | 0.000183 | EGFR |
| O75509 | Tumor necrosis factor receptor superfamily member 21 OS=Homo sapiens GN=TNFRSF21 | 0.586 | Down | 0.048022 | TNFRSF21 |
| P22692 | Insulin-like growth factor-binding protein 4 OS=Homo sapiens GN=IGFBP4 | 0.586 | Down | 0.001042 | IGFBP4 |
| O15031 | Plexin-B2 OS=Homo sapiens GN=PLXNB2 | 0.585 | Down | 0.004943 | PLXNB2 |
| Q9UKM7 | Endoplasmic reticulum mannosyl-oligosaccharide 1,2-alpha-mannosidase OS=Homo sapiens GN=MAN1B1 | 0.584 | Down | 0.013923 | MAN1B1 |
| Q9H4F8 | SPARC-related modular calcium-binding protein 1 OS=Homo sapiens GN=SMOC1 | 0.583 | Down | 5.2E-09 | SMOC1 |
| Q5T1V6 | Probable ATP-dependent RNA helicase DDX59 OS=Homo sapiens GN=DDX59 | 0.582 | Down | 0.000155 | DDX59 |
| Q10471 | Polypeptide N-acetylgalactosaminyltransferase 2 OS=Homo sapiens GN=GALNT2 | 0.581 | Down | 4.28E-06 | GALNT2 |
| P07585 | Decorin OS=Homo sapiens GN=DCN | 0.58 | Down | 4.26E-09 | DCN |
| O75973 | C1q-related factor OS=Homo sapiens GN=C1QL1 | 0.579 | Down | 0.017 | C1QL1 |
| Q7Z304 | MAM domain-containing protein 2 OS=Homo sapiens GN=MAMDC2 | 0.578 | Down | 2.92E-06 | MAMDC2 |
| O75326 | Semaphorin-7A OS=Homo sapiens GN=SEMA7A | 0.577 | Down | 0.0008 | SEMA7A |
| Q08629 | Testican-1 OS=Homo sapiens GN=SPOCK1 | 0.576 | Down | 7.66E-05 | SPOCK1 |
| Q9GZX9 | Twisted gastrulation protein homolog 1 OS=Homo sapiens GN=TWSG1 | 0.576 | Down | 0.000136 | TWSG1 |
| P48745 | Protein NOV homolog OS=Homo sapiens GN=NOV | 0.576 | Down | 0.000135 | NOV |
| Q13421 | Mesothelin OS=Homo sapiens GN=MSLN | 0.572 | Down | 3.41E-10 | MSLN |
| P27797 | Calreticulin OS=Homo sapiens GN=CALR | 0.572 | Down | 1.67E-12 | CALR |
| Q9Y4K0 | Lysyl oxidase homolog 2 OS=Homo sapiens GN=LOXL2 | 0.572 | Down | 2.41E-11 | LOXL2 |
| P25705 | ATP synthase subunit alpha, mitochondrial OS=Homo sapiens GN=ATP5A1 | 0.571 | Down | 0.018204 | ATP5A1 |
| P09603 | Macrophage colony-stimulating factor 1 OS=Homo sapiens GN=CSF1 | 0.57 | Down | 0.028138 | CSF1 |
| P03973 | Antileukoproteinase OS=Homo sapiens GN=SLPI | 0.569 | Down | 4.95E-09 | SLPI |
| Q9BZM5 | NKG2D ligand 2 OS=Homo sapiens GN=ULBP2 | 0.569 | Down | 0.01522 | ULBP2 |
| P04040 | Catalase OS=Homo sapiens GN=CAT | 0.567 | Down | 0.00014 | CAT |
| P07942 | Laminin subunit beta-1 OS=Homo sapiens GN=LAMB1 | 0.567 | Down | 1E-32 | LAMB1 |
| O00592 | Podocalyxin OS=Homo sapiens GN=PODXL | 0.566 | Down | 0.007421 | PODXL |
| P02795 | Metallothionein-2 OS=Homo sapiens GN=MT2A | 0.565 | Down | 0.040001 | MT2A |
| P05997 | Collagen alpha-2(V) chain OS=Homo sapiens GN=COL5A2 | 0.563 | Down | 2.63E-07 | COL5A2 |
| P08195 | 4F2 cell-surface antigen heavy chain OS=Homo sapiens GN=SLC3A2 | 0.56 | Down | 4.05E-07 | SLC3A2 |
| P20908 | Collagen alpha-1(V) chain OS=Homo sapiens GN=COL5A1 | 0.56 | Down | 6.26E-10 | COL5A1 |
| Q9NZ53 | Podocalyxin-like protein 2 OS=Homo sapiens GN=PODXL2 | 0.558 | Down | 0.008564 | PODXL2 |
| Q6YHK3 | CD109 antigen OS=Homo sapiens GN=CD109 | 0.557 | Down | 0.000158 | CD109 |
| P43251 | Biotinidase OS=Homo sapiens GN=BTD | 0.556 | Down | 0.026781 | BTD |
| P29279 | Connective tissue growth factor OS=Homo sapiens GN=CTGF | 0.556 | Down | 6.83E-10 | CTGF |
| P01033 | Metalloproteinase inhibitor 1 OS=Homo sapiens GN=TIMP1 | 0.555 | Down | 1.02E-08 | TIMP1 |
| Q12907 | Vesicular integral-membrane protein VIP36 OS=Homo sapiens GN=LMAN2 | 0.554 | Down | 0.0011 | LMAN2 |
| P19022 | Cadherin-2 OS=Homo sapiens GN=CDH2 | 0.554 | Down | 0.00042 | CDH2 |
| O14672 | Disintegrin and metalloproteinase domain-containing protein 10 OS=Homo sapiens GN=ADAM10 | 0.55 | Down | 0.000438 | ADAM10 |
| P05067 | Amyloid beta A4 protein OS=Homo sapiens GN=APP | 0.548 | Down | 2.57E-10 | APP |
| P17050 | Alpha-N-acetylgalactosaminidase OS=Homo sapiens GN=NAGA | 0.546 | Down | 6.06E-05 | NAGA |
| P02461 | Collagen alpha-1(III) chain OS=Homo sapiens GN=COL3A1 | 0.545 | Down | 2.42E-05 | COL3A1 |
| Q02818 | Nucleobindin-1 OS=Homo sapiens GN=NUCB1 | 0.544 | Down | 2.07E-05 | NUCB1 |
| Q99538 | Legumain OS=Homo sapiens GN=LGMN | 0.544 | Down | 1.74E-06 | LGMN |
| Q13740 | CD166 antigen OS=Homo sapiens GN=ALCAM | 0.544 | Down | 6.37E-13 | ALCAM |
| P04156 | Major prion protein OS=Homo sapiens GN=PRNP | 0.541 | Down | 0.000497 | PRNP |
| Q14118 | Dystroglycan OS=Homo sapiens GN=DAG1 | 0.541 | Down | 2.17E-12 | DAG1 |
| Q9UMX5 | Neudesin OS=Homo sapiens GN=NENF | 0.54 | Down | 0.029718 | NENF |
| Q8WVQ1 | Soluble calcium-activated nucleotidase 1 OS=Homo sapiens GN=CANT1 | 0.535 | Down | 0.001462 | CANT1 |
| O43493 | Trans-Golgi network integral membrane protein 2 OS=Homo sapiens GN=TGOLN2 | 0.534 | Down | 0.005219 | TGOLN2 |
| O00461 | Golgi integral membrane protein 4 OS=Homo sapiens GN=GOLIM4 | 0.534 | Down | 0.022382 | GOLIM4 |
| P09871 | Complement C1s subcomponent OS=Homo sapiens GN=C1S | 0.533 | Down | 0.000645 | C1S |
| P15328 | Folate receptor alpha OS=Homo sapiens GN=FOLR1 | 0.532 | Down | 7.85E-05 | FOLR1 |
| Q8NBJ4 | Golgi membrane protein 1 OS=Homo sapiens GN=GOLM1 | 0.531 | Down | 1.36E-06 | GOLM1 |
| O00115 | Deoxyribonuclease-2-alpha OS=Homo sapiens GN=DNASE2 | 0.529 | Down | 0.000297 | DNASE2 |
| P05362 | Intercellular adhesion molecule 1 OS=Homo sapiens GN=ICAM1 | 0.529 | Down | 0.031876 | ICAM1 |
| O00584 | Ribonuclease T2 OS=Homo sapiens GN=RNASET2 | 0.527 | Down | 1.67E-05 | RNASET2 |
| O00462 | Beta-mannosidase OS=Homo sapiens GN=MANBA | 0.526 | Down | 0.000158 | MANBA |
| P16070 | CD44 antigen OS=Homo sapiens GN=CD44 | 0.522 | Down | 0.00852 | CD44 |
| O95633 | Follistatin-related protein 3 OS=Homo sapiens GN=FSTL3 | 0.521 | Down | 0.00038 | FSTL3 |
| P01023 | Alpha-2-macroglobulin OS=Homo sapiens GN=A2M | 0.521 | Down | 1.62E-12 | A2M |
| Q7Z7M0 | Multiple epidermal growth factor-like domains protein 8 OS=Homo sapiens GN=MEGF8 | 0.515 | Down | 0.008799 | MEGF8 |
| Q99784 | Noelin OS=Homo sapiens GN=OLFM1 | 0.514 | Down | 1.52E-05 | OLFM1 |
| P32004 | Neural cell adhesion molecule L1 OS=Homo sapiens GN=L1CAM | 0.511 | Down | 1.65E-12 | L1CAM |
| P43121 | Cell surface glycoprotein MUC18 OS=Homo sapiens GN=MCAM | 0.51 | Down | 3.99E-05 | MCAM |
| Q9NZV1 | Cysteine-rich motor neuron 1 protein OS=Homo sapiens GN=CRIM1 | 0.51 | Down | 0.00066 | CRIM1 |
| Q92820 | Gamma-glutamyl hydrolase OS=Homo sapiens GN=GGH | 0.509 | Down | 3.13E-11 | GGH |
| P10253 | Lysosomal alpha-glucosidase OS=Homo sapiens GN=GAA | 0.507 | Down | 2.01E-07 | GAA |
| Q12841 | Follistatin-related protein 1 OS=Homo sapiens GN=FSTL1 | 0.507 | Down | 9.99E-16 | FSTL1 |
| O75882 | Attractin OS=Homo sapiens GN=ATRN | 0.506 | Down | 9.7E-05 | ATRN |
| Q01459 | Di-N-acetylchitobiase OS=Homo sapiens GN=CTBS | 0.503 | Down | 0.000101 | CTBS |
| P15586 | N-acetylglucosamine-6-sulfatase OS=Homo sapiens GN=GNS | 0.503 | Down | 1.92E-07 | GNS |
| P09486 | SPARC OS=Homo sapiens GN=SPARC | 0.503 | Down | 1.27E-10 | SPARC |
| Q9H6X2 | Anthrax toxin receptor 1 OS=Homo sapiens GN=ANTXR1 | 0.502 | Down | 0.034301 | ANTXR1 |
| P53634 | Dipeptidyl peptidase 1 OS=Homo sapiens GN=CTSC | 0.5 | Down | 0.000145 | CTSC |
| P61916 | Epididymal secretory protein E1 OS=Homo sapiens GN=NPC2 | 0.499 | Down | 8.77E-15 | NPC2 |
| Q8NHP8 | Putative phospholipase B-like 2 OS=Homo sapiens GN=PLBD2 | 0.497 | Down | 0.000722 | PLBD2 |
| P07686 | Beta-hexosaminidase subunit beta OS=Homo sapiens GN=HEXB | 0.495 | Down | 5.31E-13 | HEXB |
| Q04721 | Neurogenic locus notch homolog protein 2 OS=Homo sapiens GN=NOTCH2 | 0.492 | Down | 0.000223 | NOTCH2 |
| O95274 | Ly6/PLAUR domain-containing protein 3 OS=Homo sapiens GN=LYPD3 | 0.492 | Down | 0.000557 | LYPD3 |
| P07711 | Cathepsin L1 OS=Homo sapiens GN=CTSL | 0.49 | Down | 2E-05 | CTSL |
| P08174 | Complement decay-accelerating factor OS=Homo sapiens GN=CD55 | 0.489 | Down | 3.28E-07 | CD55 |
| Q14114 | Low-density lipoprotein receptor-related protein 8 OS=Homo sapiens GN=LRP8 | 0.486 | Down | 0.000124 | LRP8 |
| Q9HAT2 | Sialate O-acetylesterase OS=Homo sapiens GN=SIAE | 0.481 | Down | 0.000139 | SIAE |
| P13497 | Bone morphogenetic protein 1 OS=Homo sapiens GN=BMP1 | 0.481 | Down | 4.47E-13 | BMP1 |
| Q9UBR2 | Cathepsin Z OS=Homo sapiens GN=CTSZ | 0.478 | Down | 4.22E-05 | CTSZ |
| P61769 | Beta-2-microglobulin OS=Homo sapiens GN=B2M | 0.476 | Down | 3.12E-09 | B2M |
| O94907 | Dickkopf-related protein 1 OS=Homo sapiens GN=DKK1 | 0.473 | Down | 4.41E-07 | DKK1 |
| P30508 | HLA class I histocompatibility antigen, Cw-12 alpha chain OS=Homo sapiens GN=HLA-C | 0.47 | Down | 0.006841 | HLA-C |
| Q9UI42 | Carboxypeptidase A4 OS=Homo sapiens GN=CPA4 | 0.462 | Down | 3.27E-06 | CPA4 |
| P06865 | Beta-hexosaminidase subunit alpha OS=Homo sapiens GN=HEXA | 0.46 | Down | 3.21E-10 | HEXA |
| P20827 | Ephrin-A1 OS=Homo sapiens GN=EFNA1 | 0.457 | Down | 0.00446 | EFNA1 |
| P24592 | Insulin-like growth factor-binding protein 6 OS=Homo sapiens GN=IGFBP6 | 0.456 | Down | 2.14E-07 | IGFBP6 |
| Q16270 | Insulin-like growth factor-binding protein 7 OS=Homo sapiens GN=IGFBP7 | 0.452 | Down | 2.89E-15 | IGFBP7 |
| Q6UWN8 | Serine protease inhibitor Kazal-type 6 OS=Homo sapiens GN=SPINK6 | 0.45 | Down | 0.000162 | SPINK6 |
| Q92484 | Acid sphingomyelinase-like phosphodiesterase 3a OS=Homo sapiens GN=SMPDL3A | 0.444 | Down | 0.003396 | SMPDL3A |
| Q08380 | Galectin-3-binding protein OS=Homo sapiens GN=LGALS3BP | 0.443 | Down | 1.62E-12 | LGALS3BP |
| P13987 | CD59 glycoprotein OS=Homo sapiens GN=CD59 | 0.429 | Down | 0.000738 | CD59 |
| P15151 | Poliovirus receptor OS=Homo sapiens GN=PVR | 0.421 | Down | 0.018465 | PVR |
| Q8N114 | Protein shisa-5 OS=Homo sapiens GN=SHISA5 | 0.421 | Down | 0.001898 | SHISA5 |
| O43897 | Tolloid-like protein 1 OS=Homo sapiens GN=TLL1 | 0.42 | Down | 3.78E-05 | TLL1 |
| O00622 | Protein CYR61 OS=Homo sapiens GN=CYR61 | 0.416 | Down | 1E-32 | CYR61 |
| Q9NP84 | Tumor necrosis factor receptor superfamily member 12A OS=Homo sapiens GN=TNFRSF12A | 0.398 | Down | 0.000881 | TNFRSF12A |
| Q9BTY2 | Plasma alpha-L-fucosidase OS=Homo sapiens GN=FUCA2 | 0.376 | Down | 2.35E-09 | FUCA2 |

**Table S2. SW480 mass spectra data.**

| **Protein accession** | **Protein description** | **SW480-VP/**  **SW480-C Ratio** | **Regulated**  **Type** | **SW480-VP/**  **SW480-C**  **P value** | **Gene**  **name** |
| --- | --- | --- | --- | --- | --- |
| P31431 | Syndecan-4 OS=Homo sapiens GN=SDC4 | 1.562 | Up | 0.000719 | SDC4 |
| P09603 | Macrophage colony-stimulating factor 1 OS=Homo sapiens GN=CSF1 | 1.541 | Up | 0.002539 | CSF1 |
| Q9H8S9 | MOB kinase activator 1A OS=Homo sapiens GN=MOB1A | 1.523 | Up | 0.010942 | MOB1A |
| P46783 | 40S ribosomal protein S10 OS=Homo sapiens GN=RPS10 | 1.516 | Up | 0.008419 | RPS10 |
| Q15637 | Splicing factor 1 OS=Homo sapiens GN=SF1 | 1.468 | Up | 0.040404 | SF1 |
| O14737 | Programmed cell death protein 5 OS=Homo sapiens GN=PDCD5 | 1.449 | Up | 0.00918 | PDCD5 |
| Q99988 | Growth/differentiation factor 15 OS=Homo sapiens GN=GDF15 | 1.445 | Up | 0.000802 | GDF15 |
| P62633 | Cellular nucleic acid-binding protein OS=Homo sapiens GN=CNBP | 1.429 | Up | 0.000376 | CNBP |
| P06454 | Prothymosin alpha OS=Homo sapiens GN=PTMA | 1.424 | Up | 0.020322 | PTMA |
| P09341 | Growth-regulated alpha protein OS=Homo sapiens GN=CXCL1 | 1.393 | Up | 0.001616 | CXCL1 |
| Q9H3T3 | Semaphorin-6B OS=Homo sapiens GN=SEMA6B | 1.368 | Up | 0.007442 | SEMA6B |
| P16949 | Stathmin OS=Homo sapiens GN=STMN1 | 1.367 | Up | 0.0056 | STMN1 |
| P39019 | 40S ribosomal protein S19 OS=Homo sapiens GN=RPS19 | 1.363 | Up | 0.020821 | RPS19 |
| Q9NYJ1 | Cytochrome c oxidase assembly factor 4 homolog, mitochondrial OS=Homo sapiens GN=COA4 | 1.359 | Up | 0.003936 | COA4 |
| Q9P1F3 | Costars family protein ABRACL OS=Homo sapiens GN=ABRACL | 1.356 | Up | 0.013324 | ABRACL |
| Q13907 | Isopentenyl-diphosphate Delta-isomerase 1 OS=Homo sapiens GN=IDI1 | 1.354 | Up | 0.039578 | IDI1 |
| P02786 | Transferrin receptor protein 1 OS=Homo sapiens GN=TFRC | 1.354 | Up | 1.97E-05 | TFRC |
| P47813 | Eukaryotic translation initiation factor 1A, X-chromosomal OS=Homo sapiens GN=EIF1AX | 1.351 | Up | 0.031476 | EIF1AX |
| Q9NRG1 | Phosphoribosyltransferase domain-containing protein 1 OS=Homo sapiens GN=PRTFDC1 | 1.35 | Up | 0.014882 | PRTFDC1 |
| Q12907 | Vesicular integral-membrane protein VIP36 OS=Homo sapiens GN=LMAN2 | 1.346 | Up | 0.006723 | LMAN2 |
| P78406 | mRNA export factor OS=Homo sapiens GN=RAE1 | 1.323 | Up | 0.019923 | RAE1 |
| P63279 | SUMO-conjugating enzyme UBC9 OS=Homo sapiens GN=UBE2I | 1.316 | Up | 0.000416 | UBE2I |
| P52209 | 6-phosphogluconate dehydrogenase, decarboxylating OS=Homo sapiens GN=PGD | 1.315 | Up | 2.31E-06 | PGD |
| Q15843 | NEDD8 OS=Homo sapiens GN=NEDD8 | 1.31 | Up | 0.007319 | NEDD8 |
| P49006 | MARCKS-related protein OS=Homo sapiens GN=MARCKSL1 | 1.298 | Up | 0.007739 | MARCKSL1 |
| P49720 | Proteasome subunit beta type-3 OS=Homo sapiens GN=PSMB3 | 1.293 | Up | 0.031579 | PSMB3 |
| P62837 | Ubiquitin-conjugating enzyme E2 D2 OS=Homo sapiens GN=UBE2D2 | 1.281 | Up | 0.039662 | UBE2D2 |
| P07195 | L-lactate dehydrogenase B chain OS=Homo sapiens GN=LDHB | 1.278 | Up | 2.18E-05 | LDHB |
| P05114 | Non-histone chromosomal protein HMG-14 OS=Homo sapiens GN=HMGN1 | 1.273 | Up | 0.048605 | HMGN1 |
| P00441 | Superoxide dismutase [Cu-Zn] OS=Homo sapiens GN=SOD1 | 1.271 | Up | 0.006962 | SOD1 |
| P63241 | Eukaryotic translation initiation factor 5A-1 OS=Homo sapiens GN=EIF5A | 1.269 | Up | 0.008916 | EIF5A |
| Q14566 | DNA replication licensing factor MCM6 OS=Homo sapiens GN=MCM6 | 1.267 | Up | 0.004661 | MCM6 |
| Q14847 | LIM and SH3 domain protein 1 OS=Homo sapiens GN=LASP1 | 1.265 | Up | 0.01338 | LASP1 |
| O43148 | mRNA cap guanine-N7 methyltransferase OS=Homo sapiens GN=RNMT | 1.262 | Up | 0.023423 | RNMT |
| O14979 | Heterogeneous nuclear ribonucleoprotein D-like OS=Homo sapiens GN=HNRNPDL | 1.257 | Up | 0.018236 | HNRNPDL |
| P54619 | 5'-AMP-activated protein kinase subunit gamma-1 OS=Homo sapiens GN=PRKAG1 | 1.255 | Up | 0.017964 | PRKAG1 |
| Q8N114 | Protein shisa-5 OS=Homo sapiens GN=SHISA5 | 1.254 | Up | 0.036862 | SHISA5 |
| Q09028 | Histone-binding protein RBBP4 OS=Homo sapiens GN=RBBP4 | 1.254 | Up | 0.007142 | RBBP4 |
| P11940 | Polyadenylate-binding protein 1 OS=Homo sapiens GN=PABPC1 | 1.254 | Up | 0.001003 | PABPC1 |
| Q9Y617 | Phosphoserine aminotransferase OS=Homo sapiens GN=PSAT1 | 1.252 | Up | 0.007162 | PSAT1 |
| Q13283 | Ras GTPase-activating protein-binding protein 1 OS=Homo sapiens GN=G3BP1 | 1.247 | Up | 0.000897 | G3BP1 |
| Q6UWE0 | E3 ubiquitin-protein ligase LRSAM1 OS=Homo sapiens GN=LRSAM1 | 1.244 | Up | 0.049777 | LRSAM1 |
| P53041 | Serine/threonine-protein phosphatase 5 OS=Homo sapiens GN=PPP5C | 1.243 | Up | 4.13E-05 | PPP5C |
| Q15370 | Transcription elongation factor B polypeptide 2 OS=Homo sapiens GN=TCEB2 | 1.243 | Up | 0.012876 | TCEB2 |
| Q14126 | Desmoglein-2 OS=Homo sapiens GN=DSG2 | 1.24 | Up | 0.030602 | DSG2 |
| Q01844 | RNA-binding protein EWS OS=Homo sapiens GN=EWSR1 | 1.235 | Up | 0.018921 | EWSR1 |
| P53611 | Geranylgeranyl transferase type-2 subunit beta OS=Homo sapiens GN=RABGGTB | 1.235 | Up | 0.013184 | RABGGTB |
| Q9NWY4 | Histone PARylation factor 1 OS=Homo sapiens GN=HPF1 | 1.232 | Up | 0.036282 | HPF1 |
| Q9H0D6 | 5'-3' exoribonuclease 2 OS=Homo sapiens GN=XRN2 | 1.231 | Up | 0.027682 | XRN2 |
| Q14444 | Caprin-1 OS=Homo sapiens GN=CAPRIN1 | 1.225 | Up | 0.000422 | CAPRIN1 |
| Q07866 | Kinesin light chain 1 OS=Homo sapiens GN=KLC1 | 1.223 | Up | 0.039102 | KLC1 |
| Q96C90 | Protein phosphatase 1 regulatory subunit 14B OS=Homo sapiens GN=PPP1R14B | 1.222 | Up | 0.047256 | PPP1R14B |
| Q92598 | Heat shock protein 105 kDa OS=Homo sapiens GN=HSPH1 | 1.221 | Up | 0.001496 | HSPH1 |
| Q5T6V5 | UPF0553 protein C9orf64 OS=Homo sapiens GN=C9orf64 | 1.213 | Up | 0.003481 | C9orf64 |
| Q9HC38 | Glyoxalase domain-containing protein 4 OS=Homo sapiens GN=GLOD4 | 1.213 | Up | 0.002022 | GLOD4 |
| Q99615 | DnaJ homolog subfamily C member 7 OS=Homo sapiens GN=DNAJC7 | 1.213 | Up | 0.044121 | DNAJC7 |
| P33991 | DNA replication licensing factor MCM4 OS=Homo sapiens GN=MCM4 | 1.209 | Up | 0.022924 | MCM4 |
| Q9UMX0 | Ubiquilin-1 OS=Homo sapiens GN=UBQLN1 | 1.207 | Up | 0.029399 | UBQLN1 |
| O60506 | Heterogeneous nuclear ribonucleoprotein Q OS=Homo sapiens GN=SYNCRIP | 1.207 | Up | 0.031579 | SYNCRIP |
| P36405 | ADP-ribosylation factor-like protein 3 OS=Homo sapiens GN=ARL3 | 1.204 | Up | 0.03404 | ARL3 |
| P12004 | Proliferating cell nuclear antigen OS=Homo sapiens GN=PCNA | 1.204 | Up | 0.024644 | PCNA |
| P26583 | High mobility group protein B2 OS=Homo sapiens GN=HMGB2 | 0.833 | Down | 0.048638 | HMGB2 |
| Q09666 | Neuroblast differentiation-associated protein AHNAK OS=Homo sapiens GN=AHNAK | 0.831 | Down | 0.0035 | AHNAK |
| Q99715 | Collagen alpha-1(XII) chain OS=Homo sapiens GN=COL12A1 | 0.83 | Down | 0.047959 | COL12A1 |
| O94885 | SAM and SH3 domain-containing protein 1 OS=Homo sapiens GN=SASH1 | 0.829 | Down | 0.001004 | SASH1 |
| P04179 | Superoxide dismutase [Mn], mitochondrial OS=Homo sapiens GN=SOD2 | 0.829 | Down | 0.026597 | SOD2 |
| Q15185 | Prostaglandin E synthase 3 OS=Homo sapiens GN=PTGES3 | 0.829 | Down | 0.001483 | PTGES3 |
| P55268 | Laminin subunit beta-2 OS=Homo sapiens GN=LAMB2 | 0.816 | Down | 0.000656 | LAMB2 |
| Q15029 | 116 kDa U5 small nuclear ribonucleoprotein component OS=Homo sapiens GN=EFTUD2 | 0.813 | Down | 0.020705 | EFTUD2 |
| P41252 | Isoleucine--tRNA ligase, cytoplasmic OS=Homo sapiens GN=IARS | 0.812 | Down | 0.04684 | IARS |
| Q92688 | Acidic leucine-rich nuclear phosphoprotein 32 family member B OS=Homo sapiens GN=ANP32B | 0.812 | Down | 0.013761 | ANP32B |
| P23396 | 40S ribosomal protein S3 OS=Homo sapiens GN=RPS3 | 0.809 | Down | 0.001197 | RPS3 |
| Q9NPH3 | Interleukin-1 receptor accessory protein OS=Homo sapiens GN=IL1RAP | 0.809 | Down | 0.004684 | IL1RAP |
| Q7Z6Z7 | E3 ubiquitin-protein ligase HUWE1 OS=Homo sapiens GN=HUWE1 | 0.803 | Down | 0.006718 | HUWE1 |
| P61224 | Ras-related protein Rap-1b OS=Homo sapiens GN=RAP1B | 0.799 | Down | 0.014597 | RAP1B |
| P00533 | Epidermal growth factor receptor OS=Homo sapiens GN=EGFR | 0.791 | Down | 0.000423 | EGFR |
| P08195 | 4F2 cell-surface antigen heavy chain OS=Homo sapiens GN=SLC3A2 | 0.788 | Down | 0.048522 | SLC3A2 |
| Q07954 | Prolow-density lipoprotein receptor-related protein 1 OS=Homo sapiens GN=LRP1 | 0.788 | Down | 0.000178 | LRP1 |
| P09429 | High mobility group protein B1 OS=Homo sapiens GN=HMGB1 | 0.787 | Down | 5.92E-07 | HMGB1 |
| Q8IX12 | Cell division cycle and apoptosis regulator protein 1 OS=Homo sapiens GN=CCAR1 | 0.778 | Down | 0.000185 | CCAR1 |
| Q15493 | Regucalcin OS=Homo sapiens GN=RGN | 0.777 | Down | 0.024116 | RGN |
| P02751 | Fibronectin OS=Homo sapiens GN=FN1 | 0.776 | Down | 0.011138 | FN1 |
| Q969H8 | Myeloid-derived growth factor OS=Homo sapiens GN=MYDGF | 0.772 | Down | 0.010362 | MYDGF |
| P15880 | 40S ribosomal protein S2 OS=Homo sapiens GN=RPS2 | 0.772 | Down | 0.013659 | RPS2 |
| P00742 | Coagulation factor X OS=Homo sapiens GN=F10 | 0.772 | Down | 0.004541 | F10 |
| P06748 | Nucleophosmin OS=Homo sapiens GN=NPM1 | 0.768 | Down | 0.037479 | NPM1 |
| P62913 | 60S ribosomal protein L11 OS=Homo sapiens GN=RPL11 | 0.768 | Down | 0.000157 | RPL11 |
| Q9UBG0 | C-type mannose receptor 2 OS=Homo sapiens GN=MRC2 | 0.767 | Down | 0.010963 | MRC2 |
| Q9UDY2 | Tight junction protein ZO-2 OS=Homo sapiens GN=TJP2 | 0.763 | Down | 0.002641 | TJP2 |
| P63261 | Actin, cytoplasmic 2 OS=Homo sapiens GN=ACTG1 | 0.761 | Down | 8.18E-07 | ACTG1 |
| P07996 | Thrombospondin-1 OS=Homo sapiens GN=THBS1 | 0.754 | Down | 0.000357 | THBS1 |
| P22105 | Tenascin-X OS=Homo sapiens GN=TNXB | 0.752 | Down | 0.02792 | TNXB |
| Q9Y5B9 | FACT complex subunit SPT16 OS=Homo sapiens GN=SUPT16H | 0.75 | Down | 0.0025 | SUPT16H |
| Q13011 | Delta(3,5)-Delta(2,4)-dienoyl-CoA isomerase, mitochondrial OS=Homo sapiens GN=ECH1 | 0.749 | Down | 3.76E-06 | ECH1 |
| Q13418 | Integrin-linked protein kinase OS=Homo sapiens GN=ILK | 0.746 | Down | 0.011381 | ILK |
| P31040 | Succinate dehydrogenase [ubiquinone] flavoprotein subunit, mitochondrial OS=Homo sapiens GN=SDHA | 0.743 | Down | 0.038396 | SDHA |
| P36578 | 60S ribosomal protein L4 OS=Homo sapiens GN=RPL4 | 0.74 | Down | 0.000705 | RPL4 |
| Q12860 | Contactin-1 OS=Homo sapiens GN=CNTN1 | 0.74 | Down | 0.001298 | CNTN1 |
| P61457 | Pterin-4-alpha-carbinolamine dehydratase OS=Homo sapiens GN=PCBD1 | 0.74 | Down | 0.010778 | PCBD1 |
| Q15018 | BRISC complex subunit Abro1 OS=Homo sapiens GN=FAM175B | 0.739 | Down | 0.021817 | FAM175B |
| P03956 | Interstitial collagenase OS=Homo sapiens GN=MMP1 | 0.734 | Down | 0.041121 | MMP1 |
| P01024 | Complement C3 OS=Homo sapiens GN=C3 | 0.733 | Down | 0.005165 | C3 |
| P15328 | Folate receptor alpha OS=Homo sapiens GN=FOLR1 | 0.732 | Down | 0.044483 | FOLR1 |
| P27707 | Deoxycytidine kinase OS=Homo sapiens GN=DCK | 0.73 | Down | 0.032462 | DCK |
| Q4KWH8 | 1-phosphatidylinositol 4,5-bisphosphate phosphodiesterase eta-1 OS=Homo sapiens GN=PLCH1 | 0.729 | Down | 0.003542 | PLCH1 |
| P81605 | Dermcidin OS=Homo sapiens GN=DCD | 0.724 | Down | 0.000317 | DCD |
| P04040 | Catalase OS=Homo sapiens GN=CAT | 0.72 | Down | 0.009116 | CAT |
| Q6P2Q9 | Pre-mRNA-processing-splicing factor 8 OS=Homo sapiens GN=PRPF8 | 0.717 | Down | 0.041177 | PRPF8 |
| P20073 | Annexin A7 OS=Homo sapiens GN=ANXA7 | 0.717 | Down | 0.003923 | ANXA7 |
| P13591 | Neural cell adhesion molecule 1 OS=Homo sapiens GN=NCAM1 | 0.712 | Down | 0.008978 | NCAM1 |
| Q5T4S7 | E3 ubiquitin-protein ligase UBR4 OS=Homo sapiens GN=UBR4 | 0.709 | Down | 0.005077 | UBR4 |
| P08133 | Annexin A6 OS=Homo sapiens GN=ANXA6 | 0.705 | Down | 3.33E-06 | ANXA6 |
| Q99574 | Neuroserpin OS=Homo sapiens GN=SERPINI1 | 0.703 | Down | 0.022441 | SERPINI1 |
| O00622 | Protein CYR61 OS=Homo sapiens GN=CYR61 | 0.701 | Down | 0.007821 | CYR61 |
| P62906 | 60S ribosomal protein L10a OS=Homo sapiens GN=RPL10A | 0.698 | Down | 0.006005 | RPL10A |
| P02452 | Collagen alpha-1(I) chain OS=Homo sapiens GN=COL1A1 | 0.696 | Down | 2.25E-05 | COL1A1 |
| P30048 | Thioredoxin-dependent peroxide reductase, mitochondrial OS=Homo sapiens GN=PRDX3 | 0.696 | Down | 0.017602 | PRDX3 |
| O95428 | Papilin OS=Homo sapiens GN=PAPLN | 0.695 | Down | 0.048343 | PAPLN |
| P12111 | Collagen alpha-3(VI) chain OS=Homo sapiens GN=COL6A3 | 0.695 | Down | 0.036402 | COL6A3 |
| P31327 | Carbamoyl-phosphate synthase [ammonia], mitochondrial OS=Homo sapiens GN=CPS1 | 0.695 | Down | 0.021681 | CPS1 |
| Q92896 | Golgi apparatus protein 1 OS=Homo sapiens GN=GLG1 | 0.693 | Down | 5.76E-05 | GLG1 |
| P35556 | Fibrillin-2 OS=Homo sapiens GN=FBN2 | 0.691 | Down | 0.00338 | FBN2 |
| P06576 | ATP synthase subunit beta, mitochondrial OS=Homo sapiens GN=ATP5B | 0.689 | Down | 6.32E-09 | ATP5B |
| Q16629 | Serine/arginine-rich splicing factor 7 OS=Homo sapiens GN=SRSF7 | 0.685 | Down | 9.63E-05 | SRSF7 |
| Q15582 | Transforming growth factor-beta-induced protein ig-h3 OS=Homo sapiens GN=TGFBI | 0.684 | Down | 0.022303 | TGFBI |
| P07711 | Cathepsin L1 OS=Homo sapiens GN=CTSL | 0.683 | Down | 0.045702 | CTSL |
| Q92783 | Signal transducing adapter molecule 1 OS=Homo sapiens GN=STAM | 0.673 | Down | 0.016804 | STAM |
| P62753 | 40S ribosomal protein S6 OS=Homo sapiens GN=RPS6 | 0.671 | Down | 0.014482 | RPS6 |
| P05121 | Plasminogen activator inhibitor 1 OS=Homo sapiens GN=SERPINE1 | 0.669 | Down | 0.011365 | SERPINE1 |
| O15355 | Protein phosphatase 1G OS=Homo sapiens GN=PPM1G | 0.669 | Down | 0.000301 | PPM1G |
| P50995 | Annexin A11 OS=Homo sapiens GN=ANXA11 | 0.653 | Down | 0.000101 | ANXA11 |
| Q02878 | 60S ribosomal protein L6 OS=Homo sapiens GN=RPL6 | 0.652 | Down | 0.001096 | RPL6 |
| O95497 | Pantetheinase OS=Homo sapiens GN=VNN1 | 0.651 | Down | 0.047276 | VNN1 |
| P35443 | Thrombospondin-4 OS=Homo sapiens GN=THBS4 | 0.647 | Down | 0.006059 | THBS4 |
| P61247 | 40S ribosomal protein S3a OS=Homo sapiens GN=RPS3A | 0.642 | Down | 0.00028 | RPS3A |
| P46782 | 40S ribosomal protein S5 OS=Homo sapiens GN=RPS5 | 0.642 | Down | 0.003016 | RPS5 |
| Q13642 | Four and a half LIM domains protein 1 OS=Homo sapiens GN=FHL1 | 0.636 | Down | 0.049038 | FHL1 |
| Q99613 | Eukaryotic translation initiation factor 3 subunit C OS=Homo sapiens GN=EIF3C | 0.634 | Down | 1.73E-06 | EIF3C |
| B9A064 | Immunoglobulin lambda-like polypeptide 5 OS=Homo sapiens GN=IGLL5 | 0.631 | Down | 0.036145 | IGLL5 |
| Q12888 | Tumor suppressor p53-binding protein 1 OS=Homo sapiens GN=TP53BP1 | 0.622 | Down | 0.002296 | TP53BP1 |
| P62701 | 40S ribosomal protein S4, X isoform OS=Homo sapiens GN=RPS4X | 0.619 | Down | 0.003741 | RPS4X |
| P80723 | Brain acid soluble protein 1 OS=Homo sapiens GN=BASP1 | 0.616 | Down | 0.022383 | BASP1 |
| P62241 | 40S ribosomal protein S8 OS=Homo sapiens GN=RPS8 | 0.613 | Down | 0.001819 | RPS8 |
| P19338 | Nucleolin OS=Homo sapiens GN=NCL | 0.611 | Down | 1E-32 | NCL |
| P20908 | Collagen alpha-1(V) chain OS=Homo sapiens GN=COL5A1 | 0.61 | Down | 0.047803 | COL5A1 |
| P46781 | 40S ribosomal protein S9 OS=Homo sapiens GN=RPS9 | 0.609 | Down | 2.35E-05 | RPS9 |
| P35659 | Protein DEK OS=Homo sapiens GN=DEK | 0.608 | Down | 0.016561 | DEK |
| Q5TFE4 | 5'-nucleotidase domain-containing protein 1 OS=Homo sapiens GN=NT5DC1 | 0.604 | Down | 0.001463 | NT5DC1 |
| Q9Y6N7 | Roundabout homolog 1 OS=Homo sapiens GN=ROBO1 | 0.604 | Down | 0.002985 | ROBO1 |
| Q07955 | Serine/arginine-rich splicing factor 1 OS=Homo sapiens GN=SRSF1 | 0.604 | Down | 1.98E-06 | SRSF1 |
| P25705 | ATP synthase subunit alpha, mitochondrial OS=Homo sapiens GN=ATP5A1 | 0.6 | Down | 0.002642 | ATP5A1 |
| P53999 | Activated RNA polymerase II transcriptional coactivator p15 OS=Homo sapiens GN=SUB1 | 0.599 | Down | 0.001636 | SUB1 |
| Q9Y6D9 | Mitotic spindle assembly checkpoint protein MAD1 OS=Homo sapiens GN=MAD1L1 | 0.599 | Down | 0.006536 | MAD1L1 |
| Q07020 | 60S ribosomal protein L18 OS=Homo sapiens GN=RPL18 | 0.594 | Down | 0.045777 | RPL18 |
| P61769 | Beta-2-microglobulin OS=Homo sapiens GN=B2M | 0.591 | Down | 0.026503 | B2M |
| Q9UI42 | Carboxypeptidase A4 OS=Homo sapiens GN=CPA4 | 0.577 | Down | 0.006716 | CPA4 |
| P16401 | Histone H1.5 OS=Homo sapiens GN=HIST1H1B | 0.575 | Down | 0.022264 | HIST1H1B |
| P49207 | 60S ribosomal protein L34 OS=Homo sapiens GN=RPL34 | 0.574 | Down | 0.00246 | RPL34 |
| O75106 | Retina-specific copper amine oxidase OS=Homo sapiens GN=AOC2 | 0.574 | Down | 0.031279 | AOC2 |
| P07585 | Decorin OS=Homo sapiens GN=DCN | 0.571 | Down | 0.000297 | DCN |
| P11388 | DNA topoisomerase 2-alpha OS=Homo sapiens GN=TOP2A | 0.564 | Down | 0.011056 | TOP2A |
| P09525 | Annexin A4 OS=Homo sapiens GN=ANXA4 | 0.513 | Down | 0.000497 | ANXA4 |
| P21741 | Midkine OS=Homo sapiens GN=MDK | 0.511 | Down | 1.73E-07 | MDK |
| Q13247 | Serine/arginine-rich splicing factor 6 OS=Homo sapiens GN=SRSF6 | 0.47 | Down | 0.0063 | SRSF6 |
| Q9UNX3 | 60S ribosomal protein L26-like 1 OS=Homo sapiens GN=RPL26L1 | 0.461 | Down | 0.0179 | RPL26L1 |
| P30508 | HLA class I histocompatibility antigen, Cw-12 alpha chain OS=Homo sapiens GN=HLA-C | 0.422 | Down | 0.017836 | HLA-C |
| P62851 | 40S ribosomal protein S25 OS=Homo sapiens GN=RPS25 | 0.416 | Down | 8.08E-05 | RPS25 |
| Q16270 | Insulin-like growth factor-binding protein 7 OS=Homo sapiens GN=IGFBP7 | 0.286 | Down | 0.005842 | IGFBP7 |
| Q2M389 | WASH complex subunit SWIP OS=Homo sapiens GN=KIAA1033 | 0.268 | Down | 8.33E-09 | KIAA1033 |
| P31151 | Protein S100-A7 OS=Homo sapiens GN=S100A7 | 0.201 | Down | 0.001777 | S100A7 |
| Q6UWN8 | Serine protease inhibitor Kazal-type 6 OS=Homo sapiens GN=SPINK6 | 0.192 | Down | 0.019976 | SPINK6 |

**Table S3. LoVo mass spectra data.**

| **Protein accession** | **Protein description** | **LoVo-VP/**  **LoVo-C Ratio** | **Regulated Type** | **LoVo-VP/**  **LoVo-C**  **P value** | **Gene name** |
| --- | --- | --- | --- | --- | --- |
| P55287 | Cadherin-11 OS=Homo sapiens GN=CDH11 | 1.996 | Up | 0.036076 | CDH11 |
| P60953 | Cell division control protein 42 homolog OS=Homo sapiens GN=CDC42 | 1.864 | Up | 0.022021 | CDC42 |
| P49411 | Elongation factor Tu, mitochondrial OS=Homo sapiens GN=TUFM | 1.861 | Up | 0.047841 | TUFM |
| Q15818 | Neuronal pentraxin-1 OS=Homo sapiens GN=NPTX1 | 1.828 | Up | 0.016563 | NPTX1 |
| Q7Z7M0 | Multiple epidermal growth factor-like domains protein 8 OS=Homo sapiens GN=MEGF8 | 1.773 | Up | 0.045858 | MEGF8 |
| P15289 | Arylsulfatase A OS=Homo sapiens GN=ARSA | 1.761 | Up | 0.010162 | ARSA |
| Q96D15 | Reticulocalbin-3 OS=Homo sapiens GN=RCN3 | 1.733 | Up | 0.00036 | RCN3 |
| P02458 | Collagen alpha-1(II) chain OS=Homo sapiens GN=COL2A1 | 1.705 | Up | 0.001204 | COL2A1 |
| P36405 | ADP-ribosylation factor-like protein 3 OS=Homo sapiens GN=ARL3 | 1.702 | Up | 0.007716 | ARL3 |
| P35443 | Thrombospondin-4 OS=Homo sapiens GN=THBS4 | 1.688 | Up | 0.000857 | THBS4 |
| P22105 | Tenascin-X OS=Homo sapiens GN=TNXB | 1.685 | Up | 0.01264 | TNXB |
| P84077 | ADP-ribosylation factor 1 OS=Homo sapiens GN=ARF1 | 1.683 | Up | 0.018138 | ARF1 |
| O00571 | ATP-dependent RNA helicase DDX3X OS=Homo sapiens GN=DDX3X | 1.683 | Up | 0.015662 | DDX3X |
| Q9NPH3 | Interleukin-1 receptor accessory protein OS=Homo sapiens GN=IL1RAP | 1.645 | Up | 0.000144 | IL1RAP |
| P35556 | Fibrillin-2 OS=Homo sapiens GN=FBN2 | 1.628 | Up | 0.045723 | FBN2 |
| O00468 | Agrin OS=Homo sapiens GN=AGRN | 1.627 | Up | 0.000377 | AGRN |
| P12111 | Collagen alpha-3(VI) chain OS=Homo sapiens GN=COL6A3 | 1.618 | Up | 0.000416 | COL6A3 |
| B9A064 | Immunoglobulin lambda-like polypeptide 5 OS=Homo sapiens GN=IGLL5 | 1.597 | Up | 0.002617 | IGLL5 |
| Q6UWE0 | E3 ubiquitin-protein ligase LRSAM1 OS=Homo sapiens GN=LRSAM1 | 1.581 | Up | 0.002221 | LRSAM1 |
| Q15493 | Regucalcin OS=Homo sapiens GN=RGN | 1.568 | Up | 0.040917 | RGN |
| P03952 | Plasma kallikrein OS=Homo sapiens GN=KLKB1 | 1.559 | Up | 0.049015 | KLKB1 |
| O95497 | Pantetheinase OS=Homo sapiens GN=VNN1 | 1.555 | Up | 0.002603 | VNN1 |
| P03951 | Coagulation factor XI OS=Homo sapiens GN=F11 | 1.551 | Up | 0.042922 | F11 |
| P48960 | CD97 antigen OS=Homo sapiens GN=CD97 | 1.551 | Up | 0.037122 | CD97 |
| P08123 | Collagen alpha-2(I) chain OS=Homo sapiens GN=COL1A2 | 1.518 | Up | 0.006164 | COL1A2 |
| Q02809 | Procollagen-lysine,2-oxoglutarate 5-dioxygenase 1 OS=Homo sapiens GN=PLOD1 | 1.514 | Up | 0.041179 | PLOD1 |
| Q5RKV6 | Exosome complex component MTR3 OS=Homo sapiens GN=EXOSC6 | 1.511 | Up | 0.016784 | EXOSC6 |
| Q15365 | Poly(rC)-binding protein 1 OS=Homo sapiens GN=PCBP1 | 1.495 | Up | 0.012036 | PCBP1 |
| P98160 | Basement membrane-specific heparan sulfate proteoglycan core protein OS=Homo sapiens GN=HSPG2 | 1.492 | Up | 0.004877 | HSPG2 |
| P02751 | Fibronectin OS=Homo sapiens GN=FN1 | 1.489 | Up | 7.91E-05 | FN1 |
| Q8WUJ3 | Cell migration-inducing and hyaluronan-binding protein OS=Homo sapiens GN=CEMIP | 1.487 | Up | 0.008601 | CEMIP |
| P04114 | Apolipoprotein B-100 OS=Homo sapiens GN=APOB | 1.472 | Up | 0.038856 | APOB |
| P01034 | Cystatin-C OS=Homo sapiens GN=CST3 | 1.468 | Up | 0.013564 | CST3 |
| O00391 | Sulfhydryl oxidase 1 OS=Homo sapiens GN=QSOX1 | 1.455 | Up | 0.00734 | QSOX1 |
| Q13444 | Disintegrin and metalloproteinase domain-containing protein 15 OS=Homo sapiens GN=ADAM15 | 1.455 | Up | 0.021218 | ADAM15 |
| P17844 | Probable ATP-dependent RNA helicase DDX5 OS=Homo sapiens GN=DDX5 | 1.445 | Up | 0.046356 | DDX5 |
| O43175 | D-3-phosphoglycerate dehydrogenase OS=Homo sapiens GN=PHGDH | 1.443 | Up | 0.043579 | PHGDH |
| O94885 | SAM and SH3 domain-containing protein 1 OS=Homo sapiens GN=SASH1 | 1.443 | Up | 0.004661 | SASH1 |
| Q08345 | Epithelial discoidin domain-containing receptor 1 OS=Homo sapiens GN=DDR1 | 1.434 | Up | 0.000555 | DDR1 |
| O15160 | DNA-directed RNA polymerases I and III subunit RPAC1 OS=Homo sapiens GN=POLR1C | 1.433 | Up | 0.025895 | POLR1C |
| Q9Y490 | Talin-1 OS=Homo sapiens GN=TLN1 | 1.43 | Up | 1.92E-11 | TLN1 |
| P02452 | Collagen alpha-1(I) chain OS=Homo sapiens GN=COL1A1 | 1.428 | Up | 0.000361 | COL1A1 |
| Q93070 | Ecto-ADP-ribosyltransferase 4 OS=Homo sapiens GN=ART4 | 1.427 | Up | 0.004919 | ART4 |
| Q07954 | Prolow-density lipoprotein receptor-related protein 1 OS=Homo sapiens GN=LRP1 | 1.426 | Up | 0.00146 | LRP1 |
| Q8WWM7 | Ataxin-2-like protein OS=Homo sapiens GN=ATXN2L | 1.421 | Up | 0.041177 | ATXN2L |
| Q13361 | Microfibrillar-associated protein 5 OS=Homo sapiens GN=MFAP5 | 1.418 | Up | 0.011482 | MFAP5 |
| Q15582 | Transforming growth factor-beta-induced protein ig-h3 OS=Homo sapiens GN=TGFBI | 1.417 | Up | 0.010678 | TGFBI |
| Q99536 | Synaptic vesicle membrane protein VAT-1 homolog OS=Homo sapiens GN=VAT1 | 1.411 | Up | 0.020659 | VAT1 |
| P35221 | Catenin alpha-1 OS=Homo sapiens GN=CTNNA1 | 1.41 | Up | 0.016478 | CTNNA1 |
| P13591 | Neural cell adhesion molecule 1 OS=Homo sapiens GN=NCAM1 | 1.402 | Up | 0.002758 | NCAM1 |
| P28482 | Mitogen-activated protein kinase 1 OS=Homo sapiens GN=MAPK1 | 1.401 | Up | 0.029921 | MAPK1 |
| P98082 | Disabled homolog 2 OS=Homo sapiens GN=DAB2 | 1.395 | Up | 0.027363 | DAB2 |
| Q9H0B6 | Kinesin light chain 2 OS=Homo sapiens GN=KLC2 | 1.382 | Up | 0.034564 | KLC2 |
| Q9H7C9 | Mth938 domain-containing protein OS=Homo sapiens GN=AAMDC | 1.375 | Up | 0.03292 | AAMDC |
| P25205 | DNA replication licensing factor MCM3 OS=Homo sapiens GN=MCM3 | 1.361 | Up | 0.046619 | MCM3 |
| P09341 | Growth-regulated alpha protein OS=Homo sapiens GN=CXCL1 | 1.36 | Up | 0.000163 | CXCL1 |
| Q9Y6N7 | Roundabout homolog 1 OS=Homo sapiens GN=ROBO1 | 1.349 | Up | 0.002799 | ROBO1 |
| Q09666 | Neuroblast differentiation-associated protein AHNAK OS=Homo sapiens GN=AHNAK | 1.341 | Up | 0.002517 | AHNAK |
| P07814 | Bifunctional glutamate/proline--tRNA ligase OS=Homo sapiens GN=EPRS | 1.335 | Up | 0.003981 | EPRS |
| O60568 | Procollagen-lysine,2-oxoglutarate 5-dioxygenase 3 OS=Homo sapiens GN=PLOD3 | 1.332 | Up | 0.002676 | PLOD3 |
| P38571 | Lysosomal acid lipase/cholesteryl ester hydrolase OS=Homo sapiens GN=LIPA | 1.326 | Up | 0.02734 | LIPA |
| P48634 | Protein PRRC2A OS=Homo sapiens GN=PRRC2A | 1.317 | Up | 0.037304 | PRRC2A |
| O43405 | Cochlin OS=Homo sapiens GN=COCH | 1.313 | Up | 0.03128 | COCH |
| O00170 | AH receptor-interacting protein OS=Homo sapiens GN=AIP | 1.303 | Up | 0.046176 | AIP |
| P63162 | Small nuclear ribonucleoprotein-associated protein N OS=Homo sapiens GN=SNRPN | 1.297 | Up | 0.047023 | SNRPN |
| Q99715 | Collagen alpha-1(XII) chain OS=Homo sapiens GN=COL12A1 | 1.296 | Up | 0.004055 | COL12A1 |
| O00754 | Lysosomal alpha-mannosidase OS=Homo sapiens GN=MAN2B1 | 1.29 | Up | 0.001701 | MAN2B1 |
| O60911 | Cathepsin L2 OS=Homo sapiens GN=CTSV | 1.289 | Up | 0.005659 | CTSV |
| Q96HC4 | PDZ and LIM domain protein 5 OS=Homo sapiens GN=PDLIM5 | 1.288 | Up | 0.018224 | PDLIM5 |
| Q2M389 | WASH complex subunit SWIP OS=Homo sapiens GN=KIAA1033 | 1.284 | Up | 0.018318 | KIAA1033 |
| Q9HC35 | Echinoderm microtubule-associated protein-like 4 OS=Homo sapiens GN=EML4 | 1.279 | Up | 0.011603 | EML4 |
| Q9HCB6 | Spondin-1 OS=Homo sapiens GN=SPON1 | 1.261 | Up | 0.024824 | SPON1 |
| Q15149 | Plectin OS=Homo sapiens GN=PLEC | 1.261 | Up | 0.010237 | PLEC |
| Q9NSD9 | Phenylalanine--tRNA ligase beta subunit OS=Homo sapiens GN=FARSB | 1.257 | Up | 0.006036 | FARSB |
| Q12860 | Contactin-1 OS=Homo sapiens GN=CNTN1 | 1.249 | Up | 0.042324 | CNTN1 |
| Q13418 | Integrin-linked protein kinase OS=Homo sapiens GN=ILK | 1.221 | Up | 0.048379 | ILK |
| P68104 | Elongation factor 1-alpha 1 OS=Homo sapiens GN=EEF1A1 | 1.205 | Up | 0.039157 | EEF1A1 |
| P20700 | Lamin-B1 OS=Homo sapiens GN=LMNB1 | 1.204 | Up | 0.025101 | LMNB1 |
| Q96RS6 | NudC domain-containing protein 1 OS=Homo sapiens GN=NUDCD1 | 0.831 | Down | 0.029782 | NUDCD1 |
| O75312 | Zinc finger protein ZPR1 OS=Homo sapiens GN=ZPR1 | 0.828 | Down | 0.022601 | ZPR1 |
| P07237 | Protein disulfide-isomerase OS=Homo sapiens GN=P4HB | 0.826 | Down | 0.017783 | P4HB |
| P26639 | Threonine--tRNA ligase, cytoplasmic OS=Homo sapiens GN=TARS | 0.826 | Down | 0.000379 | TARS |
| Q00610 | Clathrin heavy chain 1 OS=Homo sapiens GN=CLTC | 0.822 | Down | 0.016718 | CLTC |
| P35573 | Glycogen debranching enzyme OS=Homo sapiens GN=AGL | 0.821 | Down | 0.013898 | AGL |
| P11142 | Heat shock cognate 71 kDa protein OS=Homo sapiens GN=HSPA8 | 0.81 | Down | 6.22E-05 | HSPA8 |
| P13693 | Translationally-controlled tumor protein OS=Homo sapiens GN=TPT1 | 0.808 | Down | 0.003576 | TPT1 |
| Q96CN7 | Isochorismatase domain-containing protein 1 OS=Homo sapiens GN=ISOC1 | 0.803 | Down | 0.022299 | ISOC1 |
| P05386 | 60S acidic ribosomal protein P1 OS=Homo sapiens GN=RPLP1 | 0.803 | Down | 0.031998 | RPLP1 |
| P63279 | SUMO-conjugating enzyme UBC9 OS=Homo sapiens GN=UBE2I | 0.802 | Down | 0.001565 | UBE2I |
| P15311 | Ezrin OS=Homo sapiens GN=EZR | 0.801 | Down | 2.73E-06 | EZR |
| P17174 | Aspartate aminotransferase, cytoplasmic OS=Homo sapiens GN=GOT1 | 0.8 | Down | 0.047042 | GOT1 |
| P40925 | Malate dehydrogenase, cytoplasmic OS=Homo sapiens GN=MDH1 | 0.799 | Down | 0.006823 | MDH1 |
| P00505 | Aspartate aminotransferase, mitochondrial OS=Homo sapiens GN=GOT2 | 0.798 | Down | 0.000178 | GOT2 |
| P09960 | Leukotriene A-4 hydrolase OS=Homo sapiens GN=LTA4H | 0.796 | Down | 0.011338 | LTA4H |
| Q9UKD2 | mRNA turnover protein 4 homolog OS=Homo sapiens GN=MRTO4 | 0.795 | Down | 0.046879 | MRTO4 |
| P15531 | Nucleoside diphosphate kinase A OS=Homo sapiens GN=NME1 | 0.795 | Down | 0.006001 | NME1 |
| P61604 | 10 kDa heat shock protein, mitochondrial OS=Homo sapiens GN=HSPE1 | 0.794 | Down | 8.32E-05 | HSPE1 |
| P40926 | Malate dehydrogenase, mitochondrial OS=Homo sapiens GN=MDH2 | 0.79 | Down | 0.000261 | MDH2 |
| P34932 | Heat shock 70 kDa protein 4 OS=Homo sapiens GN=HSPA4 | 0.79 | Down | 5.18E-07 | HSPA4 |
| P09429 | High mobility group protein B1 OS=Homo sapiens GN=HMGB1 | 0.789 | Down | 0.000598 | HMGB1 |
| P30101 | Protein disulfide-isomerase A3 OS=Homo sapiens GN=PDIA3 | 0.788 | Down | 0.003503 | PDIA3 |
| Q969H8 | Myeloid-derived growth factor OS=Homo sapiens GN=MYDGF | 0.787 | Down | 0.006782 | MYDGF |
| Q96G03 | Phosphoglucomutase-2 OS=Homo sapiens GN=PGM2 | 0.787 | Down | 0.01452 | PGM2 |
| P28066 | Proteasome subunit alpha type-5 OS=Homo sapiens GN=PSMA5 | 0.784 | Down | 0.047781 | PSMA5 |
| Q99613 | Eukaryotic translation initiation factor 3 subunit C OS=Homo sapiens GN=EIF3C | 0.783 | Down | 0.049241 | EIF3C |
| P63241 | Eukaryotic translation initiation factor 5A-1 OS=Homo sapiens GN=EIF5A | 0.781 | Down | 7.75E-05 | EIF5A |
| P13489 | Ribonuclease inhibitor OS=Homo sapiens GN=RNH1 | 0.78 | Down | 0.039144 | RNH1 |
| P06733 | Alpha-enolase OS=Homo sapiens GN=ENO1 | 0.779 | Down | 0.003158 | ENO1 |
| Q5TFE4 | 5'-nucleotidase domain-containing protein 1 OS=Homo sapiens GN=NT5DC1 | 0.777 | Down | 0.031858 | NT5DC1 |
| Q9HC38 | Glyoxalase domain-containing protein 4 OS=Homo sapiens GN=GLOD4 | 0.775 | Down | 0.039404 | GLOD4 |
| P10599 | Thioredoxin OS=Homo sapiens GN=TXN | 0.774 | Down | 0.036862 | TXN |
| P05060 | Secretogranin-1 OS=Homo sapiens GN=CHGB | 0.769 | Down | 0.006082 | CHGB |
| P37837 | Transaldolase OS=Homo sapiens GN=TALDO1 | 0.767 | Down | 0.002601 | TALDO1 |
| Q9UHY7 | Enolase-phosphatase E1 OS=Homo sapiens GN=ENOPH1 | 0.767 | Down | 0.004459 | ENOPH1 |
| Q99436 | Proteasome subunit beta type-7 OS=Homo sapiens GN=PSMB7 | 0.767 | Down | 0.002017 | PSMB7 |
| Q9ULA0 | Aspartyl aminopeptidase OS=Homo sapiens GN=DNPEP | 0.765 | Down | 0.004378 | DNPEP |
| P00918 | Carbonic anhydrase 2 OS=Homo sapiens GN=CA2 | 0.763 | Down | 0.008501 | CA2 |
| O95831 | Apoptosis-inducing factor 1, mitochondrial OS=Homo sapiens GN=AIFM1 | 0.76 | Down | 0.009941 | AIFM1 |
| P53611 | Geranylgeranyl transferase type-2 subunit beta OS=Homo sapiens GN=RABGGTB | 0.759 | Down | 0.049541 | RABGGTB |
| P62805 | Histone H4 OS=Homo sapiens GN=HIST1H4A | 0.755 | Down | 0.009102 | HIST1H4A |
| P42704 | Leucine-rich PPR motif-containing protein, mitochondrial OS=Homo sapiens GN=LRPPRC | 0.754 | Down | 1.67E-08 | LRPPRC |
| P08243 | Asparagine synthetase [glutamine-hydrolyzing] OS=Homo sapiens GN=ASNS | 0.754 | Down | 0.001124 | ASNS |
| P38159 | RNA-binding motif protein, X chromosome OS=Homo sapiens GN=RBMX | 0.75 | Down | 9.2E-07 | RBMX |
| Q07955 | Serine/arginine-rich splicing factor 1 OS=Homo sapiens GN=SRSF1 | 0.746 | Down | 2.2E-05 | SRSF1 |
| P00441 | Superoxide dismutase [Cu-Zn] OS=Homo sapiens GN=SOD1 | 0.737 | Down | 0.020282 | SOD1 |
| P49721 | Proteasome subunit beta type-2 OS=Homo sapiens GN=PSMB2 | 0.735 | Down | 0.001824 | PSMB2 |
| Q13685 | Angio-associated migratory cell protein OS=Homo sapiens GN=AAMP | 0.732 | Down | 0.015458 | AAMP |
| Q5T6V5 | UPF0553 protein C9orf64 OS=Homo sapiens GN=C9orf64 | 0.727 | Down | 0.006561 | C9orf64 |
| Q9BTT0 | Acidic leucine-rich nuclear phosphoprotein 32 family member E OS=Homo sapiens GN=ANP32E | 0.726 | Down | 0.018637 | ANP32E |
| Q92688 | Acidic leucine-rich nuclear phosphoprotein 32 family member B OS=Homo sapiens GN=ANP32B | 0.725 | Down | 6.12E-05 | ANP32B |
| P49458 | Signal recognition particle 9 kDa protein OS=Homo sapiens GN=SRP9 | 0.724 | Down | 0.022075 | SRP9 |
| P10809 | 60 kDa heat shock protein, mitochondrial OS=Homo sapiens GN=HSPD1 | 0.723 | Down | 0.000103 | HSPD1 |
| P11021 | 78 kDa glucose-regulated protein OS=Homo sapiens GN=HSPA5 | 0.722 | Down | 1.75E-10 | HSPA5 |
| Q9UK76 | Hematological and neurological expressed 1 protein OS=Homo sapiens GN=HN1 | 0.717 | Down | 0.029342 | HN1 |
| Q99798 | Aconitate hydratase, mitochondrial OS=Homo sapiens GN=ACO2 | 0.716 | Down | 0.028983 | ACO2 |
| Q8TDQ7 | Glucosamine-6-phosphate isomerase 2 OS=Homo sapiens GN=GNPDA2 | 0.711 | Down | 0.031545 | GNPDA2 |
| P31949 | Protein S100-A11 OS=Homo sapiens GN=S100A11 | 0.71 | Down | 0.040439 | S100A11 |
| P24534 | Elongation factor 1-beta OS=Homo sapiens GN=EEF1B2 | 0.694 | Down | 0.019856 | EEF1B2 |
| P19338 | Nucleolin OS=Homo sapiens GN=NCL | 0.693 | Down | 1.07E-09 | NCL |
| O75390 | Citrate synthase, mitochondrial OS=Homo sapiens GN=CS | 0.689 | Down | 8.27E-09 | CS |
| P62753 | 40S ribosomal protein S6 OS=Homo sapiens GN=RPS6 | 0.689 | Down | 0.046637 | RPS6 |
| P09622 | Dihydrolipoyl dehydrogenase, mitochondrial OS=Homo sapiens GN=DLD | 0.687 | Down | 0.000141 | DLD |
| P27797 | Calreticulin OS=Homo sapiens GN=CALR | 0.686 | Down | 0.000735 | CALR |
| P13667 | Protein disulfide-isomerase A4 OS=Homo sapiens GN=PDIA4 | 0.685 | Down | 6.17E-07 | PDIA4 |
| P84090 | Enhancer of rudimentary homolog OS=Homo sapiens GN=ERH | 0.683 | Down | 8.37E-05 | ERH |
| P07195 | L-lactate dehydrogenase B chain OS=Homo sapiens GN=LDHB | 0.683 | Down | 1.52E-12 | LDHB |
| P25786 | Proteasome subunit alpha type-1 OS=Homo sapiens GN=PSMA1 | 0.682 | Down | 0.012639 | PSMA1 |
| Q01105 | Protein SET OS=Homo sapiens GN=SET | 0.677 | Down | 0.020063 | SET |
| Q9Y5Z4 | Heme-binding protein 2 OS=Homo sapiens GN=HEBP2 | 0.676 | Down | 0.0019 | HEBP2 |
| Q07021 | Complement component 1 Q subcomponent-binding protein, mitochondrial OS=Homo sapiens GN=C1QBP | 0.673 | Down | 2.35E-09 | C1QBP |
| Q9GZL7 | Ribosome biogenesis protein WDR12 OS=Homo sapiens GN=WDR12 | 0.67 | Down | 0.026442 | WDR12 |
| O95881 | Thioredoxin domain-containing protein 12 OS=Homo sapiens GN=TXNDC12 | 0.664 | Down | 0.009576 | TXNDC12 |
| P62851 | 40S ribosomal protein S25 OS=Homo sapiens GN=RPS25 | 0.653 | Down | 5.54E-05 | RPS25 |
| Q71DI3 | Histone H3.2 OS=Homo sapiens GN=HIST2H3A | 0.644 | Down | 0.00398 | HIST2H3A |
| P26583 | High mobility group protein B2 OS=Homo sapiens GN=HMGB2 | 0.643 | Down | 0.005122 | HMGB2 |
| Q09028 | Histone-binding protein RBBP4 OS=Homo sapiens GN=RBBP4 | 0.639 | Down | 0.006238 | RBBP4 |
| P04179 | Superoxide dismutase [Mn], mitochondrial OS=Homo sapiens GN=SOD2 | 0.635 | Down | 0.009039 | SOD2 |
| P30048 | Thioredoxin-dependent peroxide reductase, mitochondrial OS=Homo sapiens GN=PRDX3 | 0.631 | Down | 0.013177 | PRDX3 |
| P25789 | Proteasome subunit alpha type-4 OS=Homo sapiens GN=PSMA4 | 0.616 | Down | 2.45E-05 | PSMA4 |
| P16401 | Histone H1.5 OS=Homo sapiens GN=HIST1H1B | 0.613 | Down | 0.001237 | HIST1H1B |
| P31151 | Protein S100-A7 OS=Homo sapiens GN=S100A7 | 0.601 | Down | 0.04302 | S100A7 |
| P06454 | Prothymosin alpha OS=Homo sapiens GN=PTMA | 0.589 | Down | 0.000179 | PTMA |
| P17096 | High mobility group protein HMG-I/HMG-Y OS=Homo sapiens GN=HMGA1 | 0.565 | Down | 0.041399 | HMGA1 |
| P05114 | Non-histone chromosomal protein HMG-14 OS=Homo sapiens GN=HMGN1 | 0.523 | Down | 0.017176 | HMGN1 |
